# Diverse Cyanopeptides follow distinct temporal succession patterns in freshwater harmful algal blooms

**DOI:** 10.1093/ismejo/wrag026

**Published:** 2026-02-19

**Authors:** Lauren N Hart, Reagan M Errera, Casey Godwin, Keith A Loftin, Zachary R Laughrey, Leon R Katona, Emma C Johnson, Rose M Cory, E Anders Kiledal, Paul Den Uyl, Jenan J Kharbush, David H Sherman, Gregory J Dick

**Affiliations:** Program in Chemical Biology, University of Michigan, Ann Arbor, MI 48109, United States; Life Science Institute, University of Michigan, Ann Arbor, MI 48109, United States; National Oceanic and Atmospheric Administration Great Lakes Environmental Research Laboratory, Ann Arbor, MI 48108, United States; Cooperative Institute for Great Lakes Research, School for Environment and Sustainability, University of Michigan, Ann Arbor, MI 48109, United States; U.S. Geological Survey, Central Plains Water Science Center, Algal and other Environmental Toxins Laboratory, Lawrence, KS 66049, United States; U.S. Geological Survey, Central Plains Water Science Center, Algal and other Environmental Toxins Laboratory, Lawrence, KS 66049, United States; U.S. Geological Survey, Upper Midwest Water Science Center, Lansing, MI 48911, United States; Department of Earth and Environmental Sciences, University of Michigan, Ann Arbor, MI 48109, United States; Department of Earth and Environmental Sciences, University of Michigan, Ann Arbor, MI 48109, United States; Department of Earth and Environmental Sciences, University of Michigan, Ann Arbor, MI 48109, United States; Cooperative Institute for Great Lakes Research, School for Environment and Sustainability, University of Michigan, Ann Arbor, MI 48109, United States; Department of Earth and Environmental Sciences, University of Michigan, Ann Arbor, MI 48109, United States; Life Science Institute, University of Michigan, Ann Arbor, MI 48109, United States; Departments of Medicinal Chemistry, Chemistry, Microbiology & Immunology, University of Michigan, Ann Arbor, MI 48109, United States; Cooperative Institute for Great Lakes Research, School for Environment and Sustainability, University of Michigan, Ann Arbor, MI 48109, United States; Department of Earth and Environmental Sciences, University of Michigan, Ann Arbor, MI 48109, United States

**Keywords:** metagenomics, metabolomics, harmful algal blooms, cyanobacteria, *Microcystis*, *Dolichospermum*, biosynthetic gene clusters, secondary metabolites, cyanotoxins

## Abstract

Toxic cyanobacterial harmful algal blooms (cyanoHABs) threaten freshwater resources globally and are intensifying with increasing eutrophication. Bloom toxicity is strongly influenced by intraspecific variation in the biosynthetic repertoires of toxic cyanobacteria, yet few studies examine the diversity of cyanobacterial cyanopeptides beyond hepatotoxic microcystins*.* To understand the dynamics and drivers of cyanopeptide diversity in cyanoHABs, we analyzed temporal patterns of cyanobacteria, metabolites, and their biosynthetic gene clusters (BGCs) in western Lake Erie using a 7-year time series (2016–2022) of metagenomic and metabolomic data. Our findings demonstrate that shifts from *Microcystis* to *Dolichospermum* occur later in the bloom season, coinciding with lower temperatures. Modules of co-varying BGCs (biosynthesis modules) from these genera were identified with hierarchical clustering, with uncharacterized BGCs among the most abundant. Biosynthesis modules rich in nonribosomal peptide synthetases (NRPS) peaked in early August, coinciding with elevated levels of inorganic nitrogen, warmer temperatures, and high *Microcystis* abundance. In contrast, modules rich in polyketide synthases (PKS) and ribosomally synthesized and post-translationally modified peptides (RiPPs) peaked following the *Microcystis* maximum in mid-August. Metabolomic analyses confirmed that metabolites followed shared seasonal patterns with their associated biosynthesis modules, forming three phases characterized by (i) microcystins, (ii) anabaenopeptins and aeruginosins, and (iii) aerucyclamides. These phases co-varied with bottom-up and top-down pressures, with later phases coinciding with increased microbially processed organic nitrogen and reduced detection of grazers. This study demonstrates consistent seasonal patterns of cyanobacterial metabolite succession and co-occurrence beyond microcystins, suggesting tradeoffs between biosynthetic resource demands and ecological controls.

## Graphical Abstract

**Graphical Abstract:**
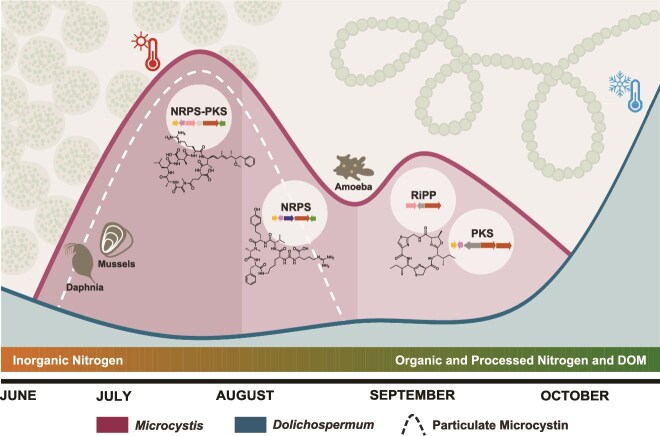

## Introduction

Cyanobacterial harmful algal blooms (cyanoHABs) degrade ecosystems and drinking water globally through the production of toxins that disrupt food webs and harm humans and wildlife [1, 2]. In temperate and sub-tropical lakes and reservoirs, two genera of bloom-forming cyanobacteria, *Microcystis* and *Dolichospermum* (previously *Anabaena*), often dominate and co-occur within cyanoHABs [3–5]. These blooms often follow a seasonal succession in which *Dolichospermum*, a diazotrophic (N₂-fixing) genus, proliferates in spring during periods of elevated nutrient loading, followed by dominance of the non-diazotrophic genus *Microcystis* under warmer, more nutrient-limited summer conditions, and a subsequent return to diazotroph dominance later in the season [3, 5]. *Microcystis* can scavenge scarce nutrients throughout shallow and low-turbulence water columns, contributing to its ability to outcompete other phytoplankton during nutrient-depleted conditions [6–8]. Conversely, *Dolichospermum* is better adapted to cooler temperatures and can persist under nitrogen (N) limitation, though it requires high phosphorus (P) availability to outcompete non-diazotrophic competitors [9, 10]. The ecological success of these two genera is also driven by a suite of adaptive traits beyond nutrient acquisition and temperature adaptation, including the production of toxic, N-rich metabolites that can deter grazing by organisms such as mussels or *Daphnia* and promote selective feeding on other phytoplankton, enhancing their competitive advantage [11].

Cyanobacteria utilize biosynthetic pathways, encoded by biosynthetic gene clusters (BGCs), to synthesize diverse bioactive secondary metabolites, primarily via nonribosomal peptide synthetase (NRPS), polyketide synthase (PKS), and ribosomally synthesized and post-translationally modified peptide (RiPPs) pathways [12]. In *Microcystis,* these BGCs encode numerous, often cryptic, metabolites, with most research focusing on the enzyme-inhibiting cyanopeptides (peptidic molecules produced by cyanobacteria) such as anabaenopeptins (NRPS), cyanopeptolins (NRPS), aeruginosins (NRPS-PKS), microcystins (NRPS-PKS), microginins (NRPS-PKS), cyanobactins (e.g. aerucyclamides, piricyclamides) (RiPPs), and microviridins (RiPPs) [13]. Although hepatotoxic microcystins dominate 90% of the freshwater cyanoHAB literature due to their potency and high concentrations within blooms [13], other cyanopeptides can reach even higher concentrations than microcystins and comprise over 80% of characterized cyanobacterial secondary metabolites, including compounds equally or more toxic than some MC congeners [14–16]. Members of the *Anabaena, Dolichospermum*, and *Aphanizomenon* (ADA) clade can produce an even greater diversity of toxins, including the neurotoxins anatoxin-a, guanitoxin, and saxitoxin (PKS-like) in addition to microcystins [17]. These metabolites affect ecosystems via lethal and behavioral effects on mammals and aquatic organisms such as zooplankton, microcrustaceans, and mussels [18–20].

Biosynthetic potential is strain-specific in bloom-forming cyanobacteria, yet studies on intraspecific variability, particularly in *Microcystis,* have largely relied on culture- and qPCR-based approaches targeting the *mcy* operon responsible for microcystin synthesis [17, 21–23]. For example, “toxigenic” (*mcy*-encoding) strains of *Microcystis* commonly occur earlier in temperate blooms, coinciding with higher temperatures and elevated inorganic N concentrations [24, 25]. Although this pattern is well documented, the ecological strategies and toxigenic potential of strains in eutrophic systems dominated by recycled organic N remain understudied [26].

Although cyanopeptide profiles of *Microcystis* isolates and single cells have been examined [27, 28], these approaches have not resolved in situ temporal dynamics or their environmental drivers from an integrated genomics–metabolomics perspective. Recent advances in metagenomics and metabolomics now enable higher-resolution analyses of these dynamics [29–31]. Despite these advances, temporal patterns of cyanobacterial secondary metabolism remain poorly understood due to limited long-term datasets, underscoring the need for sustained sampling paired with physicochemical measurements and robust phenological modeling to identify drivers of cyanopeptide synthesis beyond microcystins.

The patterns and drivers of succession between *Microcystis* and *Dolichospermum* are well documented [3, 5, 25, 32] However, the corresponding biosynthetic patterns—temporal patterns of BGCs and their products—remain understudied, leaving their dynamics in natural settings largely unexplored. Because cyanobacterial secondary metabolites are highly diverse and are produced using specialized pathways with different resource needs, we hypothesized that metabolites produced via different biosynthesis pathways will have distinct temporal patterns throughout western Lake Erie bloom progression. To characterize the dynamics of these unexplored cyanobacterial secondary metabolites, we analyzed the temporal patterns of *Microcystis* and *Dolichospermum* dominance, biosynthetic potential, and cyanopeptide abundance in 7 years (2016–2022) of metagenomics and metabolomics data from the western basin of Lake Erie. Western Lake Erie serves as a natural laboratory and model for temperate eutrophic basins experiencing cyanoHABs because it is well-monitored for its prolific seasonal blooms, and it has spatiotemporal gradients of nutrient availability from two major river (Maumee and Detroit Rivers) inputs. Results demonstrated genus-specific seasonal and interannual patterns in BGCs and that distinct cyanopeptides followed different temporal patterns throughout the bloom, co-varying with changes in nutrient pools and grazer abundance. These findings demonstrate how temporally variant ecological pressures such as nutrient availability and grazing shape the chemical diversity and functional dynamics of cyanoHABs, with implications for bloom ecology and toxin risk exposure.

## Materials and methods

### Sample collection

**Figure 1:**
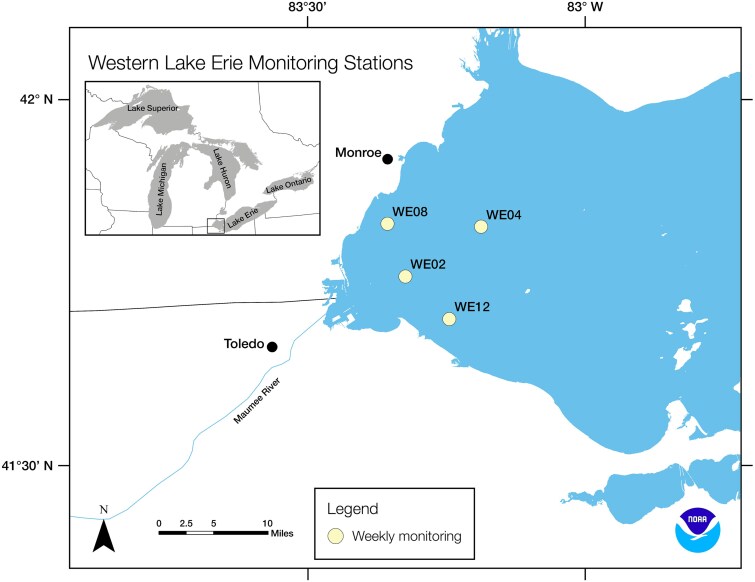
Map of western Lake Erie showing NOAA long-term monitoring stations evaluated in this study and nearby rivers and cities. Samples were collected from each site on a monthly basis between the months of May through October in 2016 through 2022. Near-shore sites WE2 and WE12 border the Ohio coastline, WE8 lies off the Michigan shoreline, and WE4 represents an offshore station. Points on the map indicate weekly monitoring stations used by NOAA GLERL.

Samples (n = 144) for DNA, metabolites, and physicochemical parameters (SI Fig. 1) were collected monthly from May through October from 2016 through 2022 at four National Oceanic and Atmospheric Administration (NOAA) Great Lakes Environmental Research Laboratory (GLERL) sampling stations (Fig. 1) in western Lake Erie [33]. Physicochemical parameters used in this study were described in Boegehold et al. (2023) [34]. Characterization of the five major fluorescence components of dissolved organic matter (FDOM) (C1–C5) was conducted as previously described (SI Table 1) [35]. Lake water was collected from one meter below the surface, filtered through a 3-μm Isopore polycarbonate membrane filter (MilliporeSigma, Burlington, MA) for both DNA and metabolite samples, and frozen at −80°C. Water was filtered until the filter clogged, which was between 5 and 450 ml of lake water.

### DNA extraction and sequencing

DNA was extracted from filters using a DNeasy Blood and Tissue Kit (Qiagen, Hilden, Germany) per the manufacturer’s instructions. The quality and quantity of DNA were assessed using a NanoDrop Life Spectrophotometer (Thermo Fisher Scientific Inc., Waltham, MA) and stored at −20°C. DNA libraries were prepared and sequenced at the University of Michigan Advanced Genomics Core using the NovaSeq System (Illumina, San Diego, CA) with a 300 cycle S4 flowcell to generate ~50 million 150 base pair paired-end reads per sample.

### Metabolite extraction and data collection

To analyze the intracellular metabolites in a polar fraction, filters were submerged in 1.5 ml of water, vortexed for 10 s, and subjected to three freeze–thaw cycles (−80°C for 20 min, 26°C water bath for 8 min). QuikLyse solution A (100 μL; Gold Standard Diagnostics, Horsham, PA) was added, followed by a 2-min shake and 8-min rest at room temperature [36]. This step was repeated using 10 μL of QuikLyse solution B. Extracts were transferred to polypropylene microcentrifuge tubes, centrifuged at 5.0 × g for 10 min at 25°C, and filtered through a 0.7 μm glass fiber filter into LC/MS vials. Samples were stored at −20°C until liquid chromatography/high resolution mass spectrometry (LC/HRMS).

Analytes were separated using the solvent gradient in SI Table 2 on a Thermo Scientific Vanquish Bioinert HPLC with a Waters Atlantis T3 column (3 μm, 3.0 × 150 mm). Mass spectrometry was performed on a Thermo Q Exactive Orbitrap with HESI-II source in positive ion mode. All samples were run in duplicate. Full HPLC and LC/HRMS protocols are described in **SI Note 1**. Data are available in Lopez et al. 2025 [37] and in the MassIVE database [38].

### Metagenomic data processing and analysis

Metagenomic reads were processed, assembled and binned using the Great Lakes Atlas of Multi-Omics Research (GLAMR) pipeline as described previously [39] (https://github.com/Geo-omics/GLAMR_omics_pipelines;  **SI Note 2**). A set of metagenome-assembled genomes (MAGs) identified as Bacteria or Archaea via GTDBtk (v 2.4.1 and database release 202 [40]) with >30% completeness and <50% contamination via CheckM [41] was dereplicated at 95% average nucleotide identity (ANI) via dRep v3.2.0 [42]. GLAMR quality-controlled (QC) reads were mapped to dereplicated MAGs using CoverM v0.6.1 [43] (MiniMap2 v.2.24) to calculate MAG relative abundance in the sequenced microbial community, accounting for genome size differences and variable sequencing effort per sample by considering the proportion of mean genome coverage depth rather than the proportion of reads [42–44]. Samples were categorized into three bloom stages based on *Microcystis* and *Dolichospermum* relative abundance differences, where Stage 1 indicates *Microcystis* dominance (>3% of all MAGs), Stage 2 indicated relative equal abundance of *Microcystis* and *Dolichospermum* (±3%)*,* and Stage 3 indicates *Dolichospermum* predominance (>3%). A 3% relative abundance difference threshold was selected based on a natural break in the distribution of *Microcystis* to *Dolichospermum* differences (SI Fig. 5E), as increasing the cutoff to 5% reclassified ~35% of samples as co-dominant and reduced sensitivity to dominance transitions.

Non-metric multidimensional scaling (NMDS) was performed on a Bray–Curtis dissimilarity matrix derived from the cyanobacterial composition of samples at the genus level with environmental variables fit as vectors. A random forest model (*randomForest* R package [45]) using 1000 trees and six-variable splits assessed variable importance in classifying bloom stage.

To assess grazer abundance, QC reads were taxonomically classified using the easy taxonomy workflow (mode 3) in MMseqs2 v2.24 against the UniRef100 database (v2022_04) [46, 47]. Relative abundance of *Daphnia* and *Dreissena* (mussels) was calculated via total sum scaling (sensitivity 4). Due to their benthic habitat, *Dreissena* detection likely indicates gametes or veligers present in the water column.

### BGC detection from metagenomes

A non-redundant, BGC database was developed to analyze temporal BGC patterns using MIBiG 3.0 and *Microcystis* BGCs previously described [23] (SI Table 3), with files and code available on GitHub, **SI Note 3,** and SI Fig. 2. QC reads were mapped to the database using MiniMap2 v2.24 (>80% coverage and identity kept) [44]. Only core BGC genes were considered due to their essential nature for biosynthesis [48]. BGCs were considered present if 90% of the core genes were covered. Most observations (n = 3135) contained 100% of core genes. We evaluated thresholds of 50%, 70%, 80%, and 90%, which resulted in modest decreases in BGC observations (3531; 3230; 3143; and 3135, respectively). A 90% threshold was selected to ensure high confidence BGC detection. Increasing the cutoff from 80% to 90% removed only seven observations, indicating minimal impact on low-abundance BGCs. Average fragments per kilobase million (FPKM) values were calculated for each BGC in each sample using Equation 1 [49].


(1) \begin{align*}& Average\ FPKM\nonumber \\ &\quad= \frac{\sum_{i=1}^{no. of\ core\ gene s\ in\ BGC}\left(\frac{{reads\ mapped\ to\ gene}_i}{\left(\frac{{gene\ length}_i}{\mathrm{1,000}}\ x\ total\ reads\ per\ sample\right)}x\ \mathrm{1,000,000}\right)}{no.\ of\ core\ gene s\ in\ BGC} \end{align*}

### Biosynthesis module analysis

To prevent skewing of distance calculations and clustering by high-frequency features, BGCs were grouped based on taxonomic origin and occurrence frequency. *Anabaena, Dolichospermum, and Aphanizomenon* (ADA) BGCs and infrequent *Microcystis* BGCs (undetectable in two or more years) were grouped, whereas consistently detected *Microcystis* BGCs were grouped. BGC abundance was hierarchically clustered (average linkage, Bray–Curtis dissimilarity), with the optimal number of clusters (k = 10) determined by silhouette scores (> 0.5). Cluster stability was assessed via 10 000 bootstraps, and clusters were defined using *cutree* (R *stats* package) [50]. Trees were visualized in the Interactive Tree of Life (iTOL) [51]. Permutation tests (n = 10 000) based on silhouette scores assessed cluster quality, with *P <* .05 and α = 0.05 indicating cohesive clusters.

### Hierarchical general additive models

Independent models for *Microcystis*, *Dolichospermum*, and the summed relative abundance (FPKM) of BGCs from individual biosynthesis modules were fit to a smoothed spline of day of year (DOY) in a generalized additive model (GAM) using 170 observations. Because these data are structured by site and year, each model was fit as a hierarchical GAM (HGAM). Response variables were natural log-transformed using ln(x + 1) to ensure model adequacy, resulting in residuals that were approximately normally distributed and centered near zero. Akaike Information Criterion (AIC) values were assessed during model selection to confirm that each term improved fit. The HGAM for *Microcystis* and *Dolichospermum* included a smooth on DOY with a cyclic cubic regression basis function, site-specific DOY smooths, a random smooth of year within site, and separate random smooths for site and year. These models assumed a Tweedie distribution, which is suitable for continuous data with many zeros such as relative abundance. The HGAM for biosynthesis modules included a smooth on DOY with a cyclic cubic regression basis function, a tensor interaction allowing DOY patterns to vary by site, a random smooth of year within site, and a separate random smooth for site. These models assumed a Gaussian distribution, appropriate for continuous data that were approximately symmetric and homoscedastic after transformation. Models were fit using the *mgcv* R package [52].

### *Microcystis* isolate analysis

BGCs from the non-redundant database were identified in *Microcystis* genomes available in the NCBI database (SI Table 4) using blastn [53]. BGC hits were considered if 50% of core genes and 50% of all genes within a BGC were present (≥70% identity and coverage). Genomes were used to build a maximum likelihood phylogenomic tree based on the prepackaged single-copy gene-set for cyanobacteria (251 target genes) using FastTree2 v2.1.11 via GToTree v.1.7.05 [51, 54, 55]. The relative abundance of western Lake Erie Culture Collection (WLECC) *Microcystis* isolates (NCBI BioProject: PRJNA903891) was calculated according to a previously described method [49] and is explained in **SI Note 2** [56].

### Metabolomics analysis

Raw data files (.d) were converted to .mzML format using MSConvert in Proteowizard v3 [57]. A suite of *in silico* tools were used to identify and annotate MS/MS features (13 428 total), an ion signal of a specific mass-to-charge ratio (*m/z)* detected at a specific retention time (RT) [31]. First, MZmine v4.3.0, using standard Orbitrap parameters (SI Table 5), was utilized for feature extraction across samples [58]. Feature intensities across samples correspond to peak areas in extracted ion-chromatograms (EICs). The feature-based molecular networking (FBMN) workflow with standard parameters in GNPS2 was run (https://gnps2.org/status?task=8c4014d3f0c547b0b4e9fdcaf6aed61c) (accessed January 21^st^, 2026) [31]. Library matches, level three as outlined in Schymanski et al. (2014), to cyanopeptides were kept and checked for characteristic fragment ions (SI Fig. 3) [59, 60]. The normalized abundance of the cyanopeptides across samples were calculated after blank removal (20% threshold, removed 4077 features) and imputation in the Functional Metabolomics Lab FBMN-STATS guide [61] using Equation 2 where AUC indicates peak area and volume filtered indicates the volume of water filtered for the corresponding sample.


(2) \begin{align*} & Normalized\ Metabolite\ Abundance \nonumber \\&\quad = \frac{AUC\ast 0.00161\ \mathrm{\mu} L}{Volume\ filtered\ \left(\mathrm{\mu} L\right)}\ast Chlorophyll\ A\ \left(\frac{\mathrm{\mu} g}{L}\right) \end{align*}

The normalized abundance of metabolite classes (congeners summed by metabolite class) and their associated BGC were Z-scored to compare relative abundance. The maxima and minima of metabolite abundance, physicochemical parameters, and grazer detection were estimated by fitting GAMs using DOY as a smooth term with a cyclic cubic regression basis, followed by 10 000 bootstrap iterations to generate 95% confidence intervals. Combinations of the five FDOM components are proxy estimates of fractions of the DOM pool: terrestrial humic DOM (Component 3 (C3)), microbially derived DOM calculated as the ratio of microbially derived to terrestrial humic DOM ((C1 + C4 + C5)/C3), and portion of aromatic amino acids within the total FDOM pool ((C4 + C5)/C1 + C2 + C3 + C4 + C5). To assess how nitrogen and DOM variables relate to metabolite abundance independent of seasonal trends, we fit GAMs to log-transformed metabolite abundances using DOY as a smooth term with a cyclic cubic regression basis. Residuals from each GAM, representing variation not explained by seasonal progression, were then extracted and plotted against individual abiotic variables in pairwise scatterplots. Linear regressions were performed on each comparison, and the resulting *R*^2^ and *P* values are reported. Figures were generated in R on R Studio using the packages *ggplot2* and *gratia* [55–57]. BGC gene schematics were generated in antiSMASH v7 [29].

## Results

### Succession of *Microcystis* to *Dolichospermum* is coincident with changes in temperature and nutrient availability

**Figure 2:**
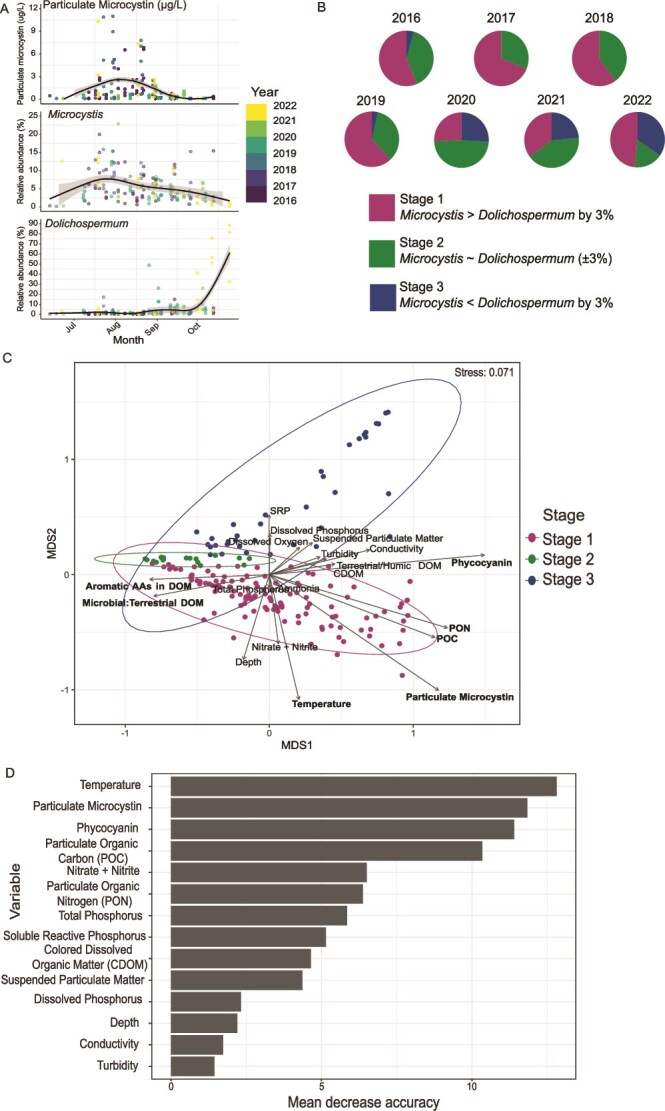
(A) Temporal patterns of particulate microcystin (μg/L) and the relative abundance of *Microcystis* and *Dolichospermum.* All individual measurements are shown as points colored by year. Particulate microcystin concentrations are shown as a black smoothed line fitted using locally weighted regression (loess). *Microcystis* and *Dolichospermum* abundance are also black smoothed lines fit using generalized additive models (GAMs) with a smooth on DOY with a cubic regression function. (B) Samples were categorized based on their relative abundance differences between *Microcystis* and *Dolichospermum.* Pie charts show the distribution of samples in each stage for each year. Sample sizes (n) in stages 1, 2, and 3, are 85, 61, and 24, respectively. (C) Nonmetric MultiDimensional scaling (NMDS) ordination of samples based on cyanobacterial community (genus level), with points colored by stage. Ellipses represent 95% confidence intervals around samples grouped by stages 1, 2, and 3. Arrows indicate the direction and strength of environmental variables, with longer arrows representing strong correlations with NMDS axes, and bolded variables indicating key drivers of ordination structure *(P <* .05). Abbreviations: Soluble reactive phosphorus (SRP), particulate organic nitrogen (PON), particulate organic carbon (POC), Colored dissolved organic matter (CDOM). (D) Random forest abiotic environmental variable importance based on mean decrease in accuracy for predicting stage, where bars represent the relative importance of each abiotic variable in classifying samples into stages.

To assess the interannual variability, seasonal patterns, and putative environmental drivers of *Microcystis* and *Dolichospermum* succession in western Lake Erie, we analyzed their relative abundance and associated variables across the dataset. *Microcystis* peaked concurrently with particulate microcystin in late July through early August (Pearson’s correlation, r = 0.42, SI Fig. 4D), whereas *Dolichospermum* increased as microcystin levels declined in late August, reaching its peak in October (Fig. 2A). Interannual variation explained 27.8% and 36.3% of deviance in hierarchical general additive models (HGAMs) for *Microcystis* and *Dolichospermum*, respectively, with site effects only contributing between 1 and 8% (SI Fig. 4, SI Table 6).

Samples were grouped into three bloom stages based on *Microcystis* and *Dolichospermum* abundance to assess interannual variation in succession patterns. Between 2016 and 2019, most samples were categorized as stages 1 and 2 (*Microcystis-*dominance or *Microcystis-Dolichospermum* co-dominance, respectively) (Fig. 2B). Most stage 3 samples (*Dolichospermum*-dominance) were observed in September and October between 2020 and 2022. Transitions between stages 1, 2, and 3 often followed a sequential pattern, but not always (SI Fig. 5).

NMDS was used to identify associations between physicochemical conditions and bloom composition (Fig. 2C, SI Fig. 6, SI Table 7). The first NMDS axis separated samples based on indicators of bloom biomass, with higher values of phycocyanin, particulate microcystin, and particulate organic carbon and nitrogen (POC, PON) aligning mostly with stage 1 samples. The second axis captured seasonal gradients of temperature, nitrate/nitrite, soluble reactive phosphorus, and total phosphorus, distinguishing between stage 1 (*Microcystis)* and stage 3 (*Dolichospermum)* samples. Despite significant associations with physicochemical parameters (PERMANOVA, *P <* .01, α = 0.05, *R*^2^ = 0–0.3, SI Table 7), samples did not distinctly cluster by stage.

Random forest classification further supported these patterns, identifying temperature, particulate microcystin, and phycocyanin as the variables most strongly associated with bloom stage classification (Fig. 2D, 58.3% accuracy, 95% Confidence Interval: 49–67%, SI Fig. 7). Measures of organic matter (POC, PON, colored dissolved organic matter (CDOM)) and dissolved and particulate nutrients also contributed to model accuracy. *Dolichospermum* abundance was significantly correlated with declining temperatures (linear regression, *P <* 2.2e-16, α = 0.05, Pearson’s correlation, r = −0.6), and analysis of surface water temperature data from WE2 between 2015 and 2023 showed an increasing frequency of cooling days in September in recent years(SI Fig. 8) [62]. Together, these results indicate that *Microcystis* to *Dolichospermum* succession varies seasonally and interannually, and may be primarily influenced by temperature and nutrient dynamics.

### Cryptic BGCs are the most abundant BGCs encoded by *Microcystis* and *Dolichospermum*

**Figure 3:**
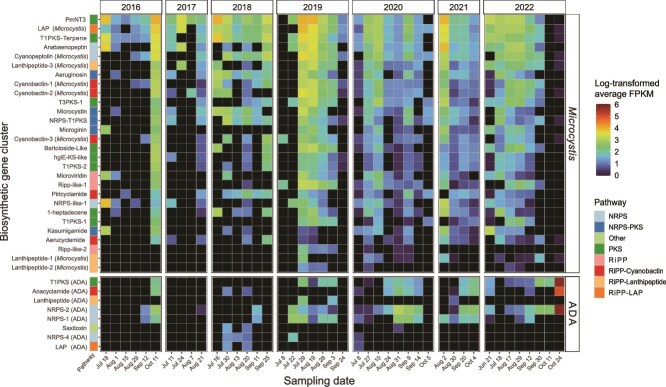
Relative abundance of BGCs from *Microcystis* and the ADA clade. Tiles are colored by log-transformed average fragments per kilobase million (FPKM) for each BGC across sampling dates within each year, with warmer colors indicating higher relative abundance. Black squares denote values of zero. BGC pathways are indicated by the color of labels on the horizontal axis.

Given the temporal variability of *Microcystis* and *Dolichospermum* abundance, we examined BGC dynamics from each genus across the time series. *Microcystis* and ADA (*Dolichospermum)* BGCs, 24 out of 37 of which were cryptic, exhibited varied temporal patterns (Fig. 3). Among ADA BGCs, anacyclamide, cryptic NRPS (NRPS-1/2), and T1PKS pathways were most abundant, occurring across all years but increasing significantly after 2019 relative to 2016–2017 (ANOVA, *P <* .05, α = 0.05), with a peak in October 2022. In contrast, cryptic RiPPs (lanthipeptide, LAP), saxitoxin, and NRPS-4 ADA BGCs appeared infrequently at low abundance between July 2018–2021.

The most frequently observed and highly abundant *Microcystis* BGCs were PmNT3, LAP, T1PKS-Terpene, lanthipeptide-3, anabaenopeptin, and cyanopeptolin (Fig. 3, SI Fig. 9) [23, 63] between late July and early August. BGCs encoding aeruginosins, microcystins, and cryptic clusters (cyanobactin-1/2, T3PKS-1, NRPS-T1PKS) occurred at lower abundance but followed similar trends. Additional cryptic BGCs (RiPP-like-2, lanthipeptide-1/2) appeared sporadically and at low abundance between 2019 and 2022. The other 13 BGCs, mainly from RiPP and PKS pathways, were found at lower abundance, or were not present such as in 2016, and often became most abundant in late August through early September. These analyses demonstrate genus-specific and temporally variable BGC abundance and the dynamic nature of cyanobacterial secondary metabolism throughout bloom progression.

### BGCs cluster into distinct biosynthesis modules with varying temporal patterns

**Figure 4:**
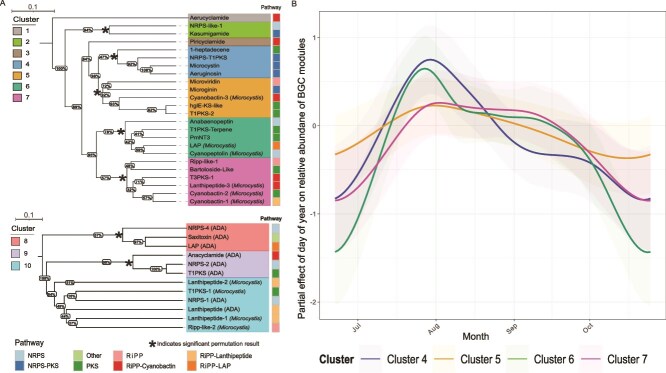
(A) Biosynthesis modules determined by hierarchical clustering (average linkage) of temporal distributions of relative abundance of BGCs based on Bray–Curtis dissimilarity. The top subtree contains *Microcystis* BGCs that occur consistently across samples, whereas the bottom subtree contains ADA BGCs and sporadically occurring *Microcystis* BGCs (zero observations in two or more years of the dataset). BGC pathways are color-coded according to the legend. An asterisk (*) denotes significantly supported modules based on permutation-derived clustering quality. Scale bars for trees are shown, where the length is equal to a branch length of 0.1. (B) Partial effects of DOY from module-specific HGAMs with the log-transformed average FPKM of BGCs from modules 4, 5, 6, and 7 as the response. Shaded ribbons indicate standard errors around the fitted temporal trends.

To identify shared temporal trends among BGCs, we performed hierarchical clustering, identifying groups of BGCs with distinct temporal profiles hereafter termed biosynthesis modules (Fig. 4A). Modules were often composed of BGCs from similar pathways, such as module four (75% NRPS-PKS), module six (80% NRPS and PKS), and module seven (66% RiPP-Cyanobactin and PKS). Conversely, module five contained BGCs from a mixture of RiPP, NRPS-PKS, and PKS pathways.

*Microcystis* biosynthesis modules followed distinct temporal patterns (Fig. 4B, SI Fig. 10, SI Table 8, *R*^2^ = 0.20–0.29). Module four, encoding the *mcy* operon, and module six peaked in late July through early August, with module four declining sharply thereafter and module six showing a secondary, weaker September peak. Module seven peaked later from August to mid-September. ADA modules eight and nine each containing a NRPS, RiPP, and PKS-like BGC, showed staggered patterns, with module eight abundant in June through July and module nine in September through October (SI Fig. 11). These data indicate that biosynthesis modules capture coherent yet temporally distinct patterns of BGC abundance, potentially reflecting coordinated secondary metabolism across *Microcystis* and *Dolichospermum*.

### Biosynthesis modules reflect mosaics of *Microcystis* isolates and their BGC content

**Figure 5:**
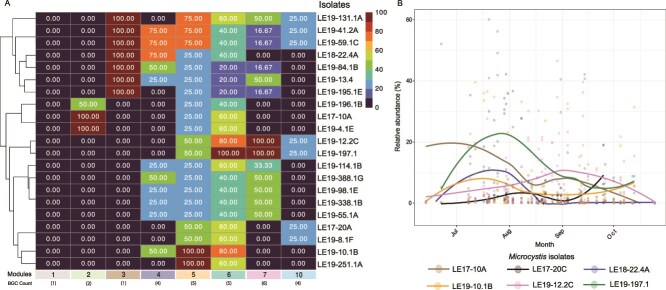
(A) Percent of each biosynthesis module present in isolates from the WLECC. Isolates are hierarchically clustered using average-linkage. Tiles are filled in by the percent of each biosynthesis module found in each isolate, with brighter tiles indicating more of the module present in that isolate. (B) Relative abundance of six WLECC isolates across field samples. Points represent individual observations, relative to the whole microbial community, and smoothed lines were fitted using a loess function.

To test the hypothesis that field-derived biosynthesis modules represent the BGC content of locally isolated *Microcystis* cultures, we calculated the percent of each module represented in MAGs recovered from unialgal, xenic *Microcystis* cultures isolated from western Lake Erie (Fig. 5A) [56]. Thirteen cultures encoded at least one complete module, but no culture encoded only one module. Module one, containing only the aerucyclamide BGC, was not represented in any of the locally relevant cultures, likely due to isolation or sequencing bias. All cultures contained at least 20% of modules five and six, with modules four and seven more sporadically detected across cultures.

Across all publicly available *Microcystis* genomes, BGCs from the same modules (e.g. microcystin and aeruginosin (four), T1PKS-Terpene and PmNT3 (six), cyanopeptolin and anabaenopeptin (six)) co-occurred most frequently (SI Fig. 12). Microcystin and anabaenopeptin BGCs did not co-occur in the publicly available genomes analyzed, with an evolutionary split between the BGCs being identified using phylogenomics (SI Fig. 13).

Four cultures encoded nearly complete modules containing four or more BGCs, such as LE18–22.4 (module four), LE19–10.1 (modules five and six), and LE19–12.2 and LE19–197.1 (modules six and seven). LE17–10 and LE17–20 are good representatives of *Microcystis* cultures with low biosynthetic potential in western Lake Erie relative to other cultures available in the culture collection. The relative abundance of LE18–22.4, LE19–197.1, LE19–10.1, and LE17–10 peaked between mid-July through early August, concurrent with peaks in microcystin and abundance of modules four, five, and six (Fig. 5B). LE17–20 and LE19–12.2 peaked later in the season between August and September, when microcystin levels declined and the relative abundance of module seven was highest. Together, these results link seasonally variable biosynthetic patterns to the dynamics of diverse *Microcystis* strains in western Lake Erie and highlight limitations of current culture collections.

### Uncoupled temporal patterns of cyanopeptides and associated BGCs

**Figure 6:**
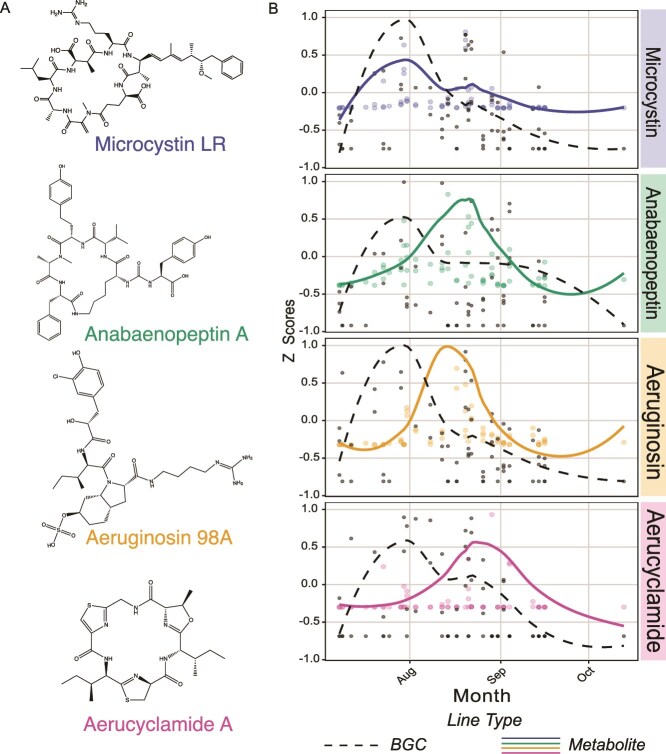
(A) Representative chemical structures of putatively annotated metabolites across the dataset: Microcystin LR, Anabaenopeptin A, Aeruginosin 98A, and Aerucyclamide A. (B) Temporal distribution of the normalized abundance of metabolites (Microcystin = sum of MC-LR, MC-YR, MC-HilR, MC-RR, and MC-HtyR; Aeruginosin 98A; Anabaenopeptin = sum of AP-A and AP-B; and Aerucyclamide ), plotted with the average FPKM of associated BGCs. Data were Z-score scaled with all individual observations less than or equal to one shown (6 observations excluded), where black dots correspond to BGC abundance and colored dots correspond to metabolite abundance. Smoothed lines for BGC and metabolite abundance, represented as black dashed and filled colored lines, respectively, were fitted using a loess function.

To assess coupling between gene abundance and metabolite production, we compared the temporal patterns of major cyanopeptides with their corresponding BGCs. Metabolomics was used to identify distinct temporal trends: microcystin peaked in late July, aeruginosin and anabaenopeptin in mid-August, and aerucyclamide from late August to early September (Fig. 6, SI Table 9, SI Fig. 14). Microcystin abundance mirrored its BGC peak, whereas aeruginosin and anabaenopeptin metabolites lagged their BGCs by 18 and 30 days, respectively. The aerucyclamide BGC showed bimodal peaks (July–early August and late August–September), yet the metabolite was detected only during the latter peak. Metabolite concentration and BGC abundance were not always temporally aligned, implying that additional regulatory or environmental factors modulate cyanopeptide synthesis in situ*.*

### Cyanopeptide maxima are associated with shifts in nitrogen, DOM, and grazer abundance

**Figure 7:**
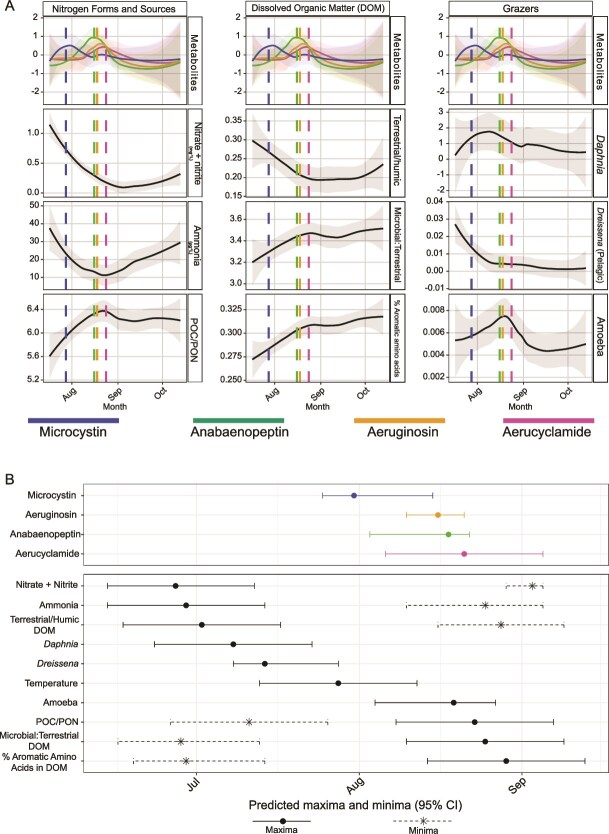
(A) Temporal patterns of the normalized abundance of metabolites (Microcystin = sum of MC-LR, MC-YR, MC-HilR, MC-RR, and MC-HtyR; Aeruginosin 98A; Anabaenopeptin = sum of AP-A and AP-B; and Aerucyclamide A) plotted with nitrogen forms/sources, DOM components, and grazer abundance in metagenomes. Metabolite data were Z-score scaled and plotted together, whereas all other variables were plotted in their original scales. Scales for each variable are as follows: Ammonia (μg L^-1^), Nitrate + Nitrate (mg L^-1^), *Daphnia*, *Dreissena*, and Amoeba (% Relative Abundance). Temporal patterns were visualized using a loess function. Dashed lines indicate predicted peak dates for each metabolite and are colored accordingly. Abbreviations: Particulate organic carbon: articulate organic nitrogen (POC/PON). (B) Predicted maxima and minima for environmental variables and metabolites were estimated using 1000 bootstrapped GAMs with a cyclic smoother on DOY. Median peak dates and 95% confidence intervals are shown as points with horizontal bars. Maxima are indicated by solid lines with circles, whereas minima, predicted only for nitrogen- and DOM-related variables, are shown as dashed lines with stars. Metabolite maxima are shown as solid-colored lines in the top panel separated from the environmental variable predictions.

Temporal variability in cyanopeptide abundance suggests regulation by environmental factors such as resource availability and grazing pressures. To evaluate these pressures, we examined the temporal patterns, maxima, and minima of cyanopeptides, nitrogen, dissolved organic matter (DOM), and grazer abundance (Fig. 7, SI Table 9). Combined nitrate and nitrite concentrations, terrestrial humic fraction of DOM, ammonia, and relative abundance of *Daphnia* and *Dreissena* in metagenomes peaked in early to mid-July, followed by temperature and microcystin peaks in late July. POC:PON ratio, microbially derived and aromatic amino acid fractions of DOM, and amoeba metagenomic relative abundance peaked in mid- to late-August, immediately following peaks in anabaenopeptin, aeruginosin, and aerucyclamide and minima of combined nitrate and nitrite, terrestrial humic DOM, and ammonia. Ammonia concentrations rose again between September and October, although not to the peak levels observed in July (Fig. 7).

Because day of year (DOY) explained roughly half the seasonal variation in cyanopeptide abundance, we assessed whether nitrogen or DOM captured additional variance. (SI Fig. 15). Nitrogen and DOM variables showed weak to moderate correlations with the residuals (*R^2^* < 0.40), suggesting limited predictive power beyond DOY. These results indicate that nitrogen concentration, DOM, and grazer dynamics correspond with, but do not fully explain, cyanopeptide variability, underscoring the multifaceted controls governing secondary metabolism during bloom progression.

## Discussion

A growing body of research has demonstrated that the production of cyanopeptides beyond microcystins is shaped by complex ecological interactions and environmental conditions, yet the temporal dynamics of these processes remain poorly understood. The BGC compositions that encode the synthesis of these metabolites vary within genera like *Microcystis* and *Dolichospermum*, which frequently co-occur and dominate freshwater cyanoHABs. Our analysis of a seven-year, metagenomic and metabolomic time series indicates coincident changes in environmental conditions and grazer abundances with three distinct phases characterized by diverse cyanopeptides in western Lake Erie.

Consistent with previous studies showing *Dolichospermum* adaptation to low temperatures and nutrient availability [3, 5], our results indicate that declining temperature and nitrogen concentrations were most associated with the late-season shift to *Dolichospermum* dominance. In contrast, the warm, N-replete yet P-depleted summer conditions favored *Microcystis*, demonstrating its exceptional P-scavenging and grazer-deterring metabolite production abilities under N sufficiency [3, 7, 64]. While previous western Lake Erie studies captured only short-term dynamics across one to two years, our seven-year dataset demonstrated a consistent shift toward *Dolichospermum* dominance beginning in 2019 and persisting through 2022. This interannual variability is not unprecedented as ADA dominance was observed in western Lake Erie in 2011 [65] but not 2014 [24, 66]. Moreover, western Lake Erie was dominated by ADA in the 1970’s prior to total P load reductions catalyzed by the Great Lakes Water Quality Agreement [67]. In addition to changing nitrogen availability, our data indicate the shift to *Dolichospermum* in 2019–2022 could be due to colder September temperatures (SI Fig. 15) that limited *Microcystis* proliferation, promoted biomass decomposition, and released bioavailable P that facilitated *Dolichospermum* growth [68]. Increased climate variability such as variable jet stream patterns causing abrupt fall cooling may further promote these successions. This study demonstrates clear BGC succession in cyanoHABs over multiple years, providing evidence for seasonally distinct biosynthesis modules—co-varying clusters of BGCs with coordinated temporal patterns that could influence bloom toxicity beyond microcystins alone. Modules dominated by NRPS and NRPS–PKS BGCs, which rely on N-rich enzymatic machinery and non-proteinogenic amino acids, were most abundant under N-replete conditions and peak microcystin abundance [69, 70]. In contrast, modules enriched in RiPPs, such as those predicted to produce cyanobactins using canonical amino acids and ribosomal machinery, and PKS BGCs, responsible for producing carbon-rich metabolites, were more abundant during periods with lower N availability [69]. These findings expand upon previous work linking nutrient availability to microcystin production and congener differentiation [71–73] by showing that nutrient controls may extend broadly across diverse classes of biosynthetic pathways and cyanopeptides.

To further explore environmental controls on cyanopeptides, we integrated metagenomic and metabolomic data, identifying three bloom phases defined by distinct cyanopeptides. These phases mirrored biosynthesis module composition and corresponded to changes in concentration of inorganic N and DOM makeup. Microcystin, nitrate, ammonia, temperature, and terrestrial humic DOM peaked early in the summer, likely indicating nutrient loads delivered via spring runoff [74]. These maxima aligned with elevated levels of NRPS and NRPS-PKS biosynthesis modules, supporting the hypothesis that microcystin production is promoted by N availability in concert with other cues like temperature [64, 71]. Two additional cyanopeptide phases occurred later in the season under different conditions. Anabaenopeptins and aeruginosins peaked in late August, followed by a peak in aerucyclamides, a RiPP-derived metabolite with on average six nitrogen atoms in its structure compared to microcystins’ ten, coincided with depleted inorganic N and increases in organic nitrogen, including aromatic amino acids. The co-variance of microbially processed DOM and inorganic N depletion with non-microcystin cyanopeptides suggests that strains lacking *mcy* gain a competitive advantage late in the bloom, a pattern observed in eutrophic lakes such as Lake Erie [26, 75], Lake Taihu [76], and Lake Champlain [77], and in *Microcystis* culturing experiments [78]. Based on these data, we hypothesize that shifts in nitrogen and DOM pools may alter cyanopeptide profiles through mechanisms such as co-regulation by shared environmental signals [79], stimulation of biosynthesis via increased carbon flux through aromatic amino acid pathways (e.g. shikimate) [80], or competitive and protective responses to accumulating DOM components [81]. Moreover, the uncoupling of the anabaenopeptin, aeruginosin, and aerucyclamide BGCs from their metabolites suggests late season conditions may favor BGC expression and metabolite synthesis. Overall, these results identified phase-specific environmental associations, with early-season cyanopeptides linked to external nutrient inputs and warming, and late-season compounds to internal recycling of organic nutrients.

In addition to metabolic triggers, ecological interactions, such as those within the phycosphere, may also influence cyanopeptide abundance. Previous studies have shown that microcystins influence the microbiome and its cross-feeding interactions with *Microcystis* [82], but the late season production of anabaenopeptins, aeruginosins, and aerucyclamides suggests different interactions. We hypothesize that these cyanopeptide phases may be driven by N remineralization and recycling mediated by the phycosphere, implying a potential associative relationship between non-*mcy* encoding *Microcystis* strains and their associated microbiome [83, 84].

Beyond interactions within the phycosphere, the shared temporal patterns of biosynthesis modules and cyanopeptides suggest broader, multi-trophic roles conferring strain-specific advantages. Certain *Microcystis* BGCs frequently co-occur, such as microcystin with aeruginosin or anabaenopeptin with cyanopeptolin, whereas microcystin and anabaenopeptin rarely coincide in the same genome, suggesting selective pressures favoring specific metabolite combinations. Although the functions of most cyanopeptides remain unsolved, they likely influence bloom dynamics individually and together via grazing deterrence, allelopathy, quorum sensing, and nutrient regulation [85–90].

Microcystin abundance peaked during periods of high *Daphnia* and *Dreissena* abundance and known activity, further supporting its role as a grazer deterrent [91, 92]. As microcystin declined, anabaenopeptin and aeruginosin increased, resulting in the co-occurrence of these three cyanopeptides. Prior studies have demonstrated synergistic and additive toxicity of these compounds in *Daphnia* [16] This second cyanopeptide phase also coincided with increased amoeba abundance, against which anabaenopeptins have demonstrated inhibitory effects, consistent with a defensive role of anabaenopeptins and aeruginosins [93]. The rare genomic co-occurrence of anabaenopeptins and microcystins despite overlapping temporal patterns with grazer populations suggests that these cyanopeptides may serve similar ecological functions or support strain-specific niche partitioning. Variation in biosynthetic capacity may mediate strain fitness throughout bloom progression, underscoring the need to investigate the ecological functions and combined effects of co-occurring metabolites under changing environmental and grazing regimes.

This study identifies and characterizes interannually conserved temporal patterns of cyanobacterial composition, biosynthetic potential, and cyanopeptide abundance in freshwater cyanoHABs, demonstrating intraspecific variability in secondary metabolism as a marker of distinct bloom populations. These successions co-varied with ecological gradients, generating testable hypotheses about environmental drivers of strain-specific fitness. The delineation of three bloom phases, each defined by unique biosynthesis modules, *Microcystis* strains, and cyanopeptides, advances our understanding of cyanoHAB chemical ecology beyond microcystins alone. The absence of microcystins does not indicate absence of risk to human and animal health, as other bioactive cyanopeptides may reach potentially toxic levels [15, 94, 95], though their regulatory thresholds remain untested. Moreover, cyanopeptide co-occurrence warrants investigation into the combinatorial toxic effects of these cyanopeptides to refine cyanoHAB risk assessment [96]. Integrating multi-omics, strain-resolved experiments, and evolutionary frameworks will be critical for understanding cyanoHAB functional complexity and anticipating how anthropogenic pressures may intensify their ecological and public health impacts.

## Supplementary Material

Supplementary-Material_wrag026

## Data Availability

All physicochemical parameters measured in this study are freely available from NOAA National Centers for Environmental Information (NCEI) [33]. Raw reads, assemblies, and MAGs used in this study were deposited on NCBI under BioProject PRJNA1190886, BioSamples SAMN45060126-SAMN45060294. All metabolomics datafiles used in this study can be found in Lopez et al. (2025) [37] and were deposited on the MassIVE database under https://doi.org/10.25345/C58W38F3P [38]. All scripts for analysis are available on GitHub at: https://github.com/Geo-omics/2016-2022-Western-Lake-Erie-Time-Series-Metagenomics-and-Metabolomics (accessed January 26th, 2026).

## References

[1] Harke MJ, Steffen MM, Gobler CJ et al. A review of the global ecology, genomics, and biogeography of the toxic cyanobacterium. *Microcystis spp Harmful Algae* 2016;54:4–20. 28073480 10.1016/j.hal.2015.12.007

[2] Chorus I., Welker M. (eds.). Toxic Cyanobacteria in Water: A Guide to their Public Health Consequences, Monitoring and Management, Vol. 2. London: Taylor & Francis, 2021, 1–859.

[3] Paerl HW, Otten TG. Duelling ‘CyanoHABs’: unravelling the environmental drivers controlling dominance and succession among diazotrophic and non-N2-fixing harmful cyanobacteria. *Environ Microbiol* 2016;18:316–24. 26310611 10.1111/1462-2920.13035

[4] Hart LN, Zepernick BN, Natwora KE et al. Metagenomics reveals spatial variation in cyanobacterial composition, function, and biosynthetic potential in the Winam gulf, Lake Victoria. *Kenya Appl and Environ Microbiol* 2025;91:507–24. 10.1128/aem.01507-24 PMC11837572 39772868

[5] Liu Y, Zhu G, Fan Y et al. Successional conditions of *Dolichospermum* and *Microcystis* in Taihu Lake. *China J Ocean Limnol* 2024;42:1777–88. 10.1007/s00343-024-4063-3

[6] Wan L, Chen X, Deng Q et al. Phosphorus strategy in bloom-forming cyanobacteria (*Dolichospermum* and *Microcystis*) and its role in their succession. *Harmful Algae* 2019;84:46–55. 10.1016/j.hal.2019.02.007 31128812

[7] Harke MJ, Berry DL, Ammerman JW et al. Molecular response of the bloom-forming cyanobacterium, *Microcystis aeruginosa*, to phosphorus limitation. *Microb Ecol* 2012;63:188–98. 10.1007/s00248-011-9894-8 21720829

[8] Harke MJ, Gobler CJ. Global transcriptional responses of the toxic cyanobacterium, *Microcystis aeruginosa*, to nitrogen stress, phosphorus stress, and growth on organic matter. *PLoS One* 2013;8:e69834. 10.1371/journal.pone.0069834 23894552 PMC3720943

[9] Paerl HW, Hall NS, Calandrino ES. Controlling harmful cyanobacterial blooms in a world experiencing anthropogenic and climatic-induced change. *Sci of The Total Environ* 2011;409:1739–45. 10.1016/j.scitotenv.2011.02.001 21345482

[10] Deng D, Meng H, Ma Y et al. The cumulative impact of temperature and nitrogen availability on the potential nitrogen fixation and extracellular polymeric substances secretion by *Dolichospermum*. *Harmful Algae* 2024;135:102633. 10.1016/j.hal.2024.102633 38830715

[11] Ger KA, Urrutia-Cordero P, Frost PC et al. The interaction between cyanobacteria and zooplankton in a more eutrophic world. *Harmful Algae* 2016;54:128–44. 10.1016/j.hal.2015.12.005 28073472

[12] He Y, Chen Y, Tao H et al. Secondary metabolites from cyanobacteria: source, chemistry, bioactivities, biosynthesis and total synthesis. *Phytochem Rev* 2024;24:483–525. 10.1007/s11101-024-09960-w

[13] Janssen EML . Cyanobacterial peptides beyond microcystins – a review on co-occurrence, toxicity, and challenges for risk assessment. *Water Res* 2019;151:488–99. 10.1016/j.watres.2018.12.048 30641464

[14] Zastepa A, Westrick JA, Miller TR et al. The Lake Erie harmful algal blooms grab: high-resolution mapping of toxic and bioactive metabolites (cyanotoxins/cyanopeptides) in cyanobacterial harmful algal blooms within the western basin. *Aquat Ecosyst Health Manage* 2024;27:46–53.

[15] Portmann C, Blom JF, Gademann K et al. Aerucyclamides a and B: isolation and synthesis of toxic ribosomal heterocyclic peptides from the cyanobacterium *Microcystis aeruginosa* PCC 7806. *J Nat Prod* 2008;71:1193–6. 10.1021/np800118g 18558743

[16] Pawlik-Skowrońska B, Bownik A. Synergistic toxicity of some cyanobacterial oligopeptides to physiological activities of *Daphnia magna* (crustacea). *Toxicon* 2022;206:74–84. 10.1016/j.toxicon.2021.12.013 34942216

[17] Österholm J, Popin RV, Fewer DP et al. Phylogenomic analysis of secondary metabolism in the toxic cyanobacterial genera *anabaena*, *Dolichospermum*, and *Aphanizomenon*. *Toxins* 2020;12:248. 10.3390/toxins12040248 32290496 PMC7232259

[18] Juhel G, Ramsay RM, Davenport J et al. Effect of the microcystin-producing cyanobacterium, *Microcystis aeruginosa*, on immune functions of the zebra mussel *Dreissena polymorpha*. *J Shellfish Res* 2015;34:433–42. 10.2983/035.034.0227

[19] Pawlik-Skowrońska B, Toporowska M, Mazur-Marzec H. Effects of secondary metabolites produced by different cyanobacterial populations on the freshwater zooplankters *Brachionus calyciflorus* and *Daphnia pulex*. *Environ Sci Pollut Res Int* 2019;26:11793–804. 10.1007/s11356-019-04543-1 30815809 PMC6476996

[20] de Torres M, Dax A, Grand I et al. Lethal and behavioral effects of semi-purified microcystins, Micropeptin and apolar compounds from cyanobacteria on freshwater microcrustacean *Thamnocephalus platyurus*. *Aquat Toxicol* 2024;273:106983. 10.1016/j.aquatox.2024.106983 38852545

[21] Al-Tebrineh J, Mihali TK, Pomati F et al. Detection of saxitoxin-producing cyanobacteria and *Anabaena circinalis* in environmental water blooms by quantitative PCR. *Appl Environ Microbiol* 2010;76:7836–42. 10.1128/AEM.00174-10 20935128 PMC2988610

[22] Otten TG, Xu H, Qin B et al. Spatiotemporal patterns and ecophysiology of toxigenic *Microcystis* blooms in Lake Taihu, China: implications for water quality management. *Environ Sci Technol* 2012;46:3480–8. 10.1021/es2041288 22324444

[23] Yancey CE, Hart L, Hefferan S et al. Metabologenomics reveals strain-level genetic and chemical diversity of *Microcystis* secondary metabolism. *mSystems* 2024;9:00334–24. 10.1128/msystems.00334-24 PMC11264947 38916306

[24] Berry MA, Davis TW, Cory RM et al. Cyanobacterial harmful algal blooms are a biological disturbance to western Lake Erie bacterial communities. *Environ Microbiol* 2017;19:1149–62. 10.1111/1462-2920.13640 28026093

[25] Tanvir RU, Hu Z, Zhang Y et al. Cyanobacterial community succession and associated cyanotoxin production in hypereutrophic and eutrophic freshwaters. *Environ Pollut* 2021;290:118056. 10.1016/j.envpol.2021.118056 34488165 PMC8547520

[26] Kharbush JJ, Robinson RS, Carter SJ. Patterns in sources and forms of nitrogen in a large eutrophic lake during a cyanobacterial harmful algal bloom. *Limnol Oceanogr* 2023;68:803–15. 10.1002/lno.12311

[27] Lezcano MÁ, Agha R, Cirés S et al. Spatial-temporal survey of *Microcystis* oligopeptide chemotypes in reservoirs with dissimilar waterbody features and their relation to genetic variation. *Harmful Algae* 2019;81:77–85. 10.1016/j.hal.2018.11.009 30638501

[28] Agha R, Àngeles Lezcano M, del Mar LM et al. Seasonal dynamics and sedimentation patterns of *Microcystis* oligopeptide-based chemotypes reveal subpopulations with different ecological traits. *Limnol Oceanogr* 2014;59:861–71.

[29] Blin K, Shaw S, Augustijn HE et al. antiSMASH 7.0: new and improved predictions for detection, regulation, chemical structures and visualization. *Nucleic Acids Res* 2023;51:W46–50. 10.1093/nar/gkad344 37140036 PMC10320115

[30] Terlouw BR, Blin K, Navarro-Muñoz JC et al. MIBiG 3.0: a community-driven effort to annotate experimentally validated biosynthetic gene clusters. *Nucleic Acids Res* 2023;51:D603–10. 10.1093/nar/gkac1049 36399496 PMC9825592

[31] Nothias LF, Petras D, Schmid R et al. Feature-based molecular networking in the GNPS analysis environment. *Nat Methods* 2020;17:905–8. 10.1038/s41592-020-0933-6 32839597 PMC7885687

[32] Paerl HW, Scott JT, McCarthy MJ et al. It takes two to tango: when and where dual nutrient (N & P) reductions are needed to Protect Lakes and downstream ecosystems. *Environ Sci Technol* 2016;50:10805–13. 10.1021/acs.est.6b02575 27667268

[33] Cooperative Institute for Great Lakes Research UOM . NOAA Great Lakes Environmental Research Laboratory. Physical, Chemical, and Biological Water Quality Monitoring Data to Support Detection of Harmful Algal Blooms (HABs) in Western Lake Erie, Collected by the Great Lakes Environmental Research Laboratory and the Cooperative Institute for Great Lakes Research since 2012. Michigan, United States: NOAA National Centers for Environmental Information, 2019. Available from: https://www.ncei.noaa.gov/archive/accession/GLERL-CIGLR-HAB-LakeErie-water-qual.

[34] Boegehold AG, Burtner AM, Camilleri AC et al. Routine monitoring of western Lake Erie to track water quality changes associated with cyanobacterial harmful algal blooms. *Earth System Science Data* 2023;15:3853–68. 10.5194/essd-15-3853-2023

[35] Cory RM, Davis TW, Dick GJ et al. Seasonal dynamics in dissolved organic matter, hydrogen peroxide, and cyanobacterial blooms in Lake Erie. *Front Mar Sci* 2016;3:1–17. 10.3389/fmars.2016.00054

[36] Greenstein KE, Zamyadi A, Wert EC. Comparative assessment of physical and chemical Cyanobacteria cell lysis methods for Total microcystin-LR analysis. *Toxins* 2021;13:596. 10.3390/toxins13090596 34564601 PMC8473049

[37] Lopez AE, Laughrey ZR, Loftin KA. Qualitative Analysis of Cyanotoxins in Lake Erie Samples Using Liquid Chromatography/High Resolution Mass Spectrometry (LC/HRMS) from July 2016 to October 2022: U.S. Geological Survey Data Release. Reston, VA, USA: U.S. Geological Survey, 2025. 10.5066/P1XNTTVC

[38] Dick G . MassIVE MSV000097661 - Western Lake Erie Qualitative Time Series (2016–2020, WE2, WE4, WE12, WE8): This Dataset Consists of Non-targeted Metabolomics Samples Collected Via NOAA GLERL and CIGLR in Western Lake Erie Sampling of Sites WE2, WE12, WE4, and WE8 between the Years of 2016 through 2020. Data Was Generated by USGS. MassIVE. San Diego, CA, USA: University of California, 2025, [cited 2026 Jan 21]. Available from: https://massive.ucsd.edu/ProteoSAFe/dataset.jsp?accession=MSV000097661.

[39] Den Uyl PA, Kiledal EA, Errera RM et al. Genomic identification and characterization of saxitoxin producing Cyanobacteria in western Lake Erie harmful algal blooms. *Environ Sci Technol* 2025;59:7600–12. 10.1021/acs.est.4c10888 40209228 PMC12782359

[40] Parks DH, Chuvochina M, Rinke C et al. GTDB: an ongoing census of bacterial and archaeal diversity through a phylogenetically consistent, rank normalized and complete genome-based taxonomy. *Nucleic Acids Res* 2022;50:D785–94. 10.1093/nar/gkab776 34520557 PMC8728215

[41] Parks DH, Imelfort M, Skennerton CT et al. CheckM: assessing the quality of microbial genomes recovered from isolates, single cells, and metagenomes. *Genome Res* 2015;25:1043–55. 10.1101/gr.186072.114 25977477 PMC4484387

[42] Olm MR, Brown CT, Brooks B et al. dRep: a tool for fast and accurate genomic comparisons that enables improved genome recovery from metagenomes through de-replication. *ISME J* 2017;11:2864–8. 10.1038/ismej.2017.126 28742071 PMC5702732

[43] Woodcroft BJ . CoverM: Read Coverage Calculator for Metagenomics. Woolloongabba, Australia: Center for Microbiome Research. [Internet]. [cited 2022 Jun 6]. Available from: https://github.com/wwood/CoverM

[44] Li H . Minimap2: pairwise alignment for nucleotide sequences. *Bioinformatics* 2018;34:3094–100. 10.1093/bioinformatics/bty191 29750242 PMC6137996

[45] Liaw A, Wiener M. Classification and regression by “randomForest.”. *R News* 2002;2:18–22.

[46] Steinegger M, Söding J. MMseqs2 enables sensitive protein sequence searching for the analysis of massive data sets. *Nat Biotechnol* 2017;35:1026–8. 10.1038/nbt.3988 29035372

[47] Suzek BE, Wang Y, Huang H et al. UniRef clusters: a comprehensive and scalable alternative for improving sequence similarity searches. *Bioinformatics* 2015;31:926–32. 10.1093/bioinformatics/btu739 25398609 PMC4375400

[48] Medema MH, Kottmann R, Yilmaz P et al. Minimum information about a biosynthetic gene cluster. *Nat Chem Biol* 2015;11:625–31. 26284661 10.1038/nchembio.1890 PMC5714517

[49] Trapnell C, Williams BA, Pertea G et al. Transcript assembly and quantification by RNA-Seq reveals unannotated transcripts and isoform switching during cell differentiation. *Nat Biotechnol* 2010;28:511–5. 10.1038/nbt.1621 20436464 PMC3146043

[50] R Core Team . R: A Language and Environment for Statistical Computing [Internet]. Vienna, Austria: R Foundation for Statistical Computing, 2013, Available from: http://www.R-project.org/.

[51] Letunic I, Bork P. Interactive tree of life (iTOL) v5: an online tool for phylogenetic tree display and annotation. *Nucleic Acids Res* 2021;49:W293–6. 10.1093/nar/gkab301 33885785 PMC8265157

[52] Wood SN . Fast stable restricted maximum likelihood and marginal likelihood estimation of semiparametric generalized linear models. Journal of the Royal Statistical Society 2011;73:3–36. 10.1111/j.1467-9868.2010.00749.x

[53] Camacho C, Coulouris G, Avagyan V et al. BLAST+: architecture and applications. *BMC Bioinformatics* 2009;10:421. 10.1186/1471-2105-10-421 20003500 PMC2803857

[54] Lee MD . GToTree: a user-friendly workflow for phylogenomics. *Bioinformatics* 2019;35:4162–4. 10.1093/bioinformatics/btz188 30865266 PMC6792077

[55] Price MN, Dehal PS, Arkin AP. FastTree 2 – approximately maximum-likelihood trees for large alignments. *PLoS One* 2010;5:e9490. 10.1371/journal.pone.0009490 20224823 PMC2835736

[56] Yancey CE, Kiledal EA, Chaganti SR et al. The western Lake Erie culture collection: a promising resource for evaluating the physiological and genetic diversity of *Microcystis* and its associated microbiome. *Harmful Algae* 2023;126:102440. 10.1016/j.hal.2023.102440 37290887

[57] Chambers MC, Maclean B, Burke R et al. A cross-platform toolkit for mass spectrometry and proteomics. *Nat Biotechnol* 2012;30:918–20. 10.1038/nbt.2377 23051804 PMC3471674

[58] Schmid R, Heuckeroth S, Korf A et al. Integrative analysis of multimodal mass spectrometry data in MZmine 3. *Nat Biotechnol* 2023;41:447–9. 10.1038/s41587-023-01690-2 36859716 PMC10496610

[59] Schymanski EL, Jeon J, Gulde R et al. Identifying small molecules via high resolution mass spectrometry: communicating confidence. *Environ Sci Technol* 2014;48:2097–8. 10.1021/es5002105 24476540

[60] McDonald K, DesRochers N, Renaud JB et al. Metabolomics reveals strain-specific cyanopeptide profiles and their production dynamics in *Microcystis aeruginosa* and *M. Flos-aquae*. *Toxins* 2023;15:1–18. 10.3390/toxins15040254 PMC10147050 37104192

[61] Pakkir Shah AK, Walter A, Ottosson F et al. Statistical analysis of feature-based molecular networking results from non-targeted metabolomics data. *Nat Protoc* 2025;20:92–162. 10.1038/s41596-024-01046-3 39304763

[62] NOAA . NOAA WE2 nutrient buoy data from 2015-2023 [internet]. Great Lakes environmental research. *Laboratory* [cited 2025 Jul 1]. Available from: https://seagull-erddap.glos.org/erddap/tabledap/GLERLWE2_archive.html.

[63] Larsen J, Type III, Biosynthesis P et al. School of Environmental and Life Sciences. Australia: University of Newcastle, 2023, [cited 2025 Mar 3]. Available from: https://nova.newcastle.edu.au/vital/access/services/Download/uon:50347/ATTACHMENT01.

[64] Harke MJ, Davis TW, Watson SB et al. Nutrient-controlled niche differentiation of western Lake Erie cyanobacterial populations revealed via metatranscriptomic surveys. *Environ Science & Tech* 2016;50:604–15. 10.1021/acs.est.5b03931 26654276

[65] Michalak AM, Anderson EJ, Beletsky D et al. Record-setting algal bloom in Lake Erie caused by agricultural and meteorological trends consistent with expected future conditions. *Proc Natl Acad Sci* 2013;110:6448–52. 10.1073/pnas.1216006110 23576718 PMC3631662

[66] Yancey CE, Mathiesen O, Dick GJ. Transcriptionally active nitrogen fixation and biosynthesis of diverse secondary metabolites by *Dolichospermum* and *Aphanizomenon*-like cyanobacteria in western Lake Erie *Microcystis* blooms. *Harmful Algae* 2023;124:102408. 10.1016/j.hal.2023.102408 37164563

[67] Munawar M, Munawar IF. A Lakewide study of phytoplankton biomass and its species composition in Lake Erie, April–December 1970. *J Fish Res Bd Can* 1976;33:581–600. 10.1139/f76-075

[68] Wang Y, Chen F. Decomposition and phosphorus release from four different size fractions of *Microcystis* spp. taken from Lake Taihu, China. Journal of. *Environ Sci* 2008;20:891–6. 10.1016/S1001-0742(08)62143-9 18814588

[69] Wenski SL, Thiengmag S, Helfrich EJN. Complex peptide natural products: biosynthetic principles, challenges and opportunities for pathway engineering. *Synthetic and Systems Biotechnol* 2022;7:631–47. 10.1016/j.synbio.2022.01.007 PMC8842026 35224231

[70] Walsh CT, O’Brien RV, Khosla C. Nonproteinogenic amino acid building blocks for nonribosomal peptide and hybrid polyketide scaffolds. *Angew Chem Int Ed Engl* 2013;52:7098–124. 10.1002/anie.201208344 23729217 PMC4634941

[71] Chaffin JD, Westrick JA, Reitz LA et al. Microcystin congeners in Lake Erie follow the seasonal pattern of nitrogen availability. *Harmful Algae* 2023;127:102466. 10.1016/j.hal.2023.102466 37544667 PMC10867787

[72] Van de Waal DB, Smith VH, Declerck SAJ et al. Stoichiometric regulation of phytoplankton toxins. *Ecol Lett* 2014;17:736–42. 24712512 10.1111/ele.12280

[73] Wagner ND, Osburn FS, Wang J et al. Biological stoichiometry regulates toxin production in *Microcystis aeruginosa* (UTEX 2385). *Toxins* 2019;11:601. 31623095 10.3390/toxins11100601 PMC6833104

[74] Watson SB, Miller C, Arhonditsis G et al. The re-eutrophication of Lake Erie: harmful algal blooms and hypoxia. *Harmful Algae* 2016;56:44–66. 10.1016/j.hal.2016.04.010 28073496

[75] Hoffman DK, McCarthy MJ, Boedecker AR et al. The role of internal nitrogen loading in supporting non-N-fixing harmful cyanobacterial blooms in the water column of a large eutrophic lake. *Limnol Oceanogr* 2022;67:2028–41.

[76] Hampel JJ, McCarthy MJ, Gardner WS et al. Nitrification and ammonium dynamics in Taihu Lake, China: seasonal competition for ammonium between nitrifiers and cyanobacteria. *Biogeosciences* 2018;15:733–48. 10.5194/bg-15-733-2018

[77] McCarthy MJ, Gardner WS, Lehmann MF et al. Implications of water column ammonium uptake and regeneration for the nitrogen budget in temperate, eutrophic Missisquoi Bay, Lake Champlain (Canada/USA). *Hydrobiologia* 2013;718:173–88. 10.1007/s10750-013-1614-6

[78] Briand E, Bormans M, Gugger M et al. Changes in secondary metabolic profiles of *Microcystis aeruginosa* strains in response to intraspecific interactions. *Environ Microbiol* 2016;18:384–400. 10.1111/1462-2920.12904 25980449 PMC5083810

[79] Kosakowska A, Nedzi M, Pempkowiak J. Responses of the toxic cyanobacterium *Microcystis aeruginosa* to iron and humic substances. *Plant Physiol Biochem* 2007;45:365–70. 10.1016/j.plaphy.2007.03.024 17509890

[80] Shende VV, Bauman KD, Moore BS. The shikimate pathway: gateway to metabolic diversity. *Nat Prod Rep* 2024;41:604–48. 10.1039/D3NP00037K 38170905 PMC11043010

[81] Chen J, Zhou Y, Zhang Y. New insights into microbial degradation of cyanobacterial organic matter using a fractionation procedure. *Int J Environ Res Public Health* 2022;19:6981. 10.3390/ijerph19126981 35742228 PMC9222324

[82] Große R, Heuser M, Teikari JE et al. Microcystin shapes the *Microcystis* phycosphere through community filtering and by influencing cross-feeding interactions. *ISME Commun* 2024;5:170. 10.1093/ismeco/ycae170 PMC11748430 39839888

[83] Wang K, Mou X, Cao H et al. Co-occurring microorganisms regulate the succession of cyanobacterial harmful algal blooms. *Environ Pollut* 2021;288:117682. 10.1016/j.envpol.2021.117682 34271516 PMC8478823

[84] Li W, Baliu-Rodriguez D, Premathilaka SH et al. Microbiome processing of organic nitrogen input supports growth and cyanotoxin production of *Microcystis aeruginosa* cultures. *ISME J* 2024;18:wrae082. 10.1093/ismejo/wrae082 PMC11126159 38718148

[85] Ger KA, Faassen EJ, Pennino MG et al. Effect of the toxin (microcystin) content of *Microcystis* on copepod grazing. *Harmful Algae* 2016;52:34–45. 10.1016/j.hal.2015.12.008 28073469

[86] Rohrlack T, Christoffersen K, Kaebernick M et al. Cyanobacterial protease inhibitor microviridin J causes a lethal Molting disruption in *Daphnia pulicaria*. *Appl Environ Microbiol* 2004;70:5047–50. 10.1128/AEM.70.8.5047-5050.2004 15294849 PMC492328

[87] Pereira DA, Giani A. Cell density-dependent oligopeptide production in cyanobacterial strains. *FEMS Microbiol Ecol* 2014;88:175–83. 10.1111/1574-6941.12281 24410818

[88] Sukenik A, Eshkol R, Livne A et al. Inhibition of growth and photosynthesis of the dinoflagellate *Peridinium gatunense* by *Microcystis* sp. (cyanobacteria): a novel allelopathic mechanism. *Limnol Oceanogr* 2002;47:1656–63.

[89] Zilliges Y, Kehr JC, Meissner S et al. The cyanobacterial hepatotoxin microcystin binds to proteins and increases the fitness of *Microcystis* under oxidative stress conditions. *PLoS One* 2011;6:e17615. 10.1371/journal.pone.0017615 21445264 PMC3060824

[90] Krausfeldt LE, Farmer AT, Castro HF et al. Nitrogen flux into metabolites and microcystins changes in response to different nitrogen sources in *Microcystis aeruginosa* NIES-843. *Environ Microbiol* 2020;22:2419–31. 10.1111/1462-2920.15032 32338427 PMC9008454

[91] Vanderploeg HA, Liebig JR, Carmichael WW et al. Zebra mussel (*Dreissena polymorpha*) selective filtration promoted toxic *Microcystis* blooms in Saginaw Bay (Lake Huron) and Lake Erie. *Can J Fish Aquat Sci* 2001;58:1208–21. 10.1139/f01-066

[92] Boegehold AG, Glyshaw P, Vanderploeg HA et al. *Microcystis* strains in Lake Erie explain interactions between a selective filter feeder and the phytoplankton community. *Hydrobiologia* 2025;852:3703–18. 10.1007/s10750-025-05839-9

[93] Urrutia-Cordero P, Agha R, Cirés S et al. Effects of harmful cyanobacteria on the freshwater pathogenic free-living amoeba *Acanthamoeba castellanii*. *Aquat Toxicol* 2013;130-131:9–17. 10.1016/j.aquatox.2012.12.019 23333903

[94] Halland N, Brönstrup M, Czech J et al. Novel small molecule inhibitors of activated thrombin activatable fibrinolysis inhibitor (TAFIa) from natural product anabaenopeptin. *J Med Chem* 2015;58:4839–44. 10.1021/jm501840b 25990761

[95] Kohler E, Grundler V, Häussinger D et al. The toxicity and enzyme activity of a chlorine and sulfate containing aeruginosin isolated from a non-microcystin-producing *Planktothrix* strain. *Harmful Algae* 2014;39:154–60. 10.1016/j.hal.2014.07.003 28100989 PMC5238944

[96] Hart LN, Lev KL, Hefferan S et al. Cyanopeptide mixtures induce variable synergistic and antagonistic effects across diverse human cell lines. *Environ Toxicol* 2026;1–15. 10.1002/tox.70028 PMC13537360 41518131

