# Supplementary material for: Diverse Cyanopeptides follow distinct temporal succession patterns in freshwater harmful algal blooms
Source: ISME J. 2026 Feb 19;20(1):wrag026. doi: 10.1093/ismejo/wrag026 (PMC13196583; doi:10.1093/ismejo/wrag026)
Supplement: Supplementary-Material_wrag026 [file supplementary-material_wrag026.zip › Clean_SI_wrag026(1).docx]

**Supplementary Information for the Manuscript Titled, “*“Diverse Cyanopeptides Follow Distinct Temporal Succession Patterns in Freshwater Harmful Algal Blooms”***

Lauren N. Hart^1,2^, Reagan Errera^3^, Casey Godwin^4^, Keith A. Loftin^5^, Zachary R. Laughrey^5^, Leon R. Katona^6^, Emma C. Johnson^7^, Rose M. Cory^7^, E. Anders Kiledal^7^, Paul Den Uyl^4^, Jenan J. Kharbush^7^, David H. Sherman^2,8^, Gregory J. Dick^4,8^

*^1^Program in Chemical Biology, University of Michigan, Ann Arbor, MI, USA*

*^2^Life Science Institute, University of Michigan, Ann Arbor, MI, USA*

*^3^National Oceanic and Atmospheric Administration Great Lakes Environmental Research Laboratory, Ann Arbor, MI, USA*

*^4^Cooperative Institute for Great Lakes Research, School for Environment and Sustainability, University of Michigan, Ann Arbor, MI, USA*

*^5^U.S. Geological Survey, Central Plains Water Science Center, Algal and other Environmental Toxins Laboratory, Lawrence, KS, USA*

*^6^U.S. Geological Survey, Upper Midwest Water Science Center, Lansing, MI, USA*

*^7^Department of Earth and Environmental Sciences, University of Michigan, Ann Arbor, MI, USA*

*^8^Departments of Medicinal Chemistry, Chemistry, Microbiology & Immunology, University of Michigan, Ann Arbor, MI, USA*

Any use of trade, firm, or product names is for descriptive purposes only and does not imply endorsement by the U.S. Government.

**SI Tables (all in attached .xlsx SI table file)**

**SI Table 1:** Metadata for all samples used in this study, including sampling and physicochemical parameter information.

**SI Table 2**: HPLC Solvent Gradient

**SI Table 3**: Biosynthetic gene cluster (BGC) database information

**SI Table 4**: *Microcystis* genomes used for phylogenomic analysis.

**SI Table 5**: MZmine Parameters used in the feature-finding processing wizard.

**SI Table 6**: Hierarchical general additive models (HGAM) equation and results for the relative abundance (%) of *Microcystis* and *Dolichospermum* relative to the whole microbial community.

**SI Table 7:** R^2^ and *P*-values for NMDS vectors in Figure 2C**.**

**SI Table 8:** Hierarchical general additive models (HGAM) equation and results for the FPKM of BGCs within modules 4, 5, 6 and 7.

**SI Table 9:** Residual standard errors of loess fits of metabolite, dissolved organic matter (DOM), gene, nitrogen, and grazer abundance patterns.

**________________________________________________________________**

**SI Figures**

**
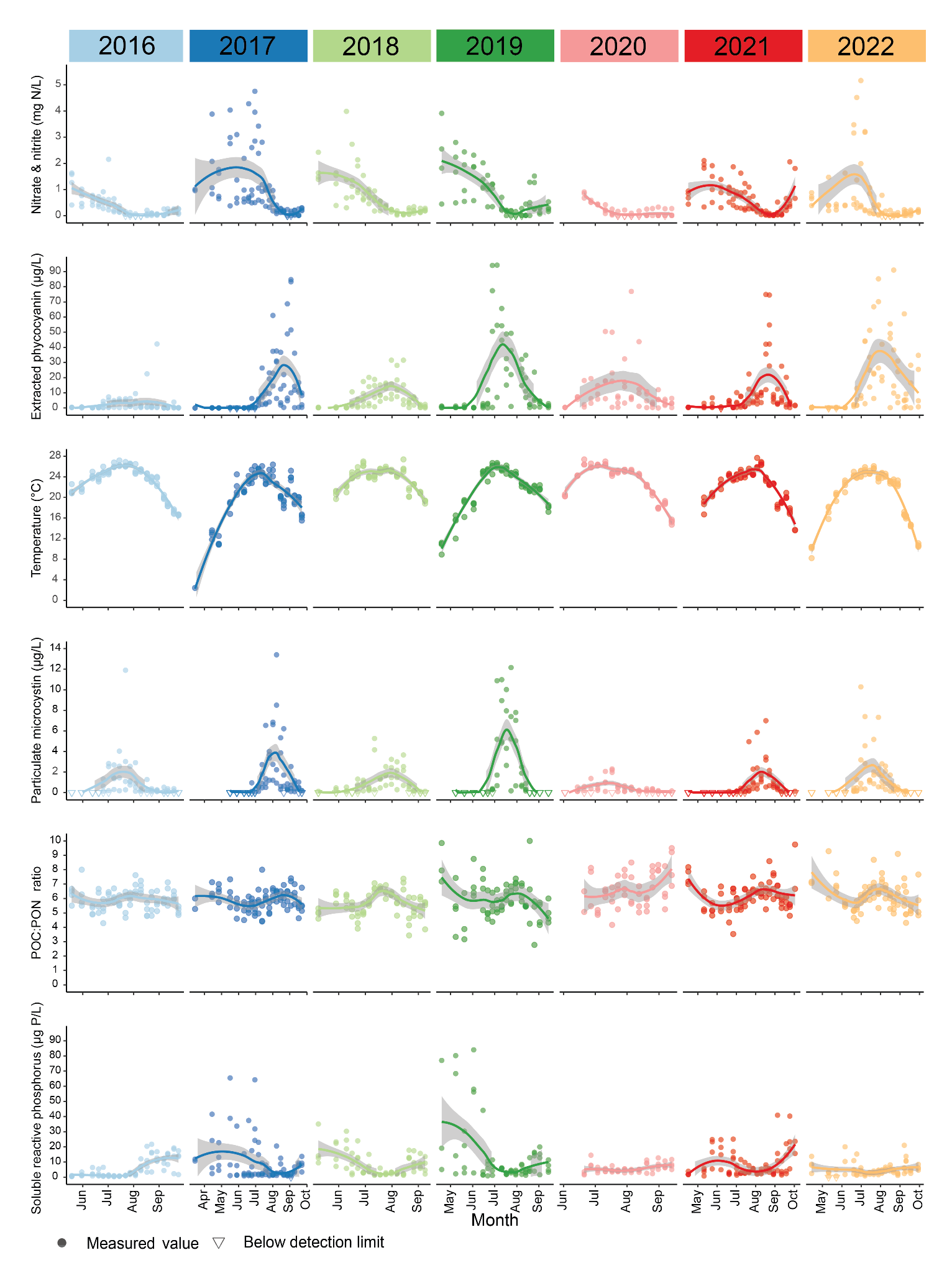
**

**SI Figure 1:** Comparison of nitrate + nitrite (mg/L), extracted phycocyanin (µg/L), temperature (°C), particulate microcystin (µg/L), particulate organic carbon: particulate organic nitrogen ratio (POC:PON), and soluble reactive phosphorus (µg P/L) from all stations between the years of 2016 and 2022. Individual observations are plotted as filled in circles, with observations below the detection limit plotted as unfilled triangles. Values under the detection limit were only identified for particulate microcystins and nitrate + nitrite and were imputed as half the limit of detection. Smoothed lines were fit using a loess function.

1. **1-heptadecene_20_0**

**
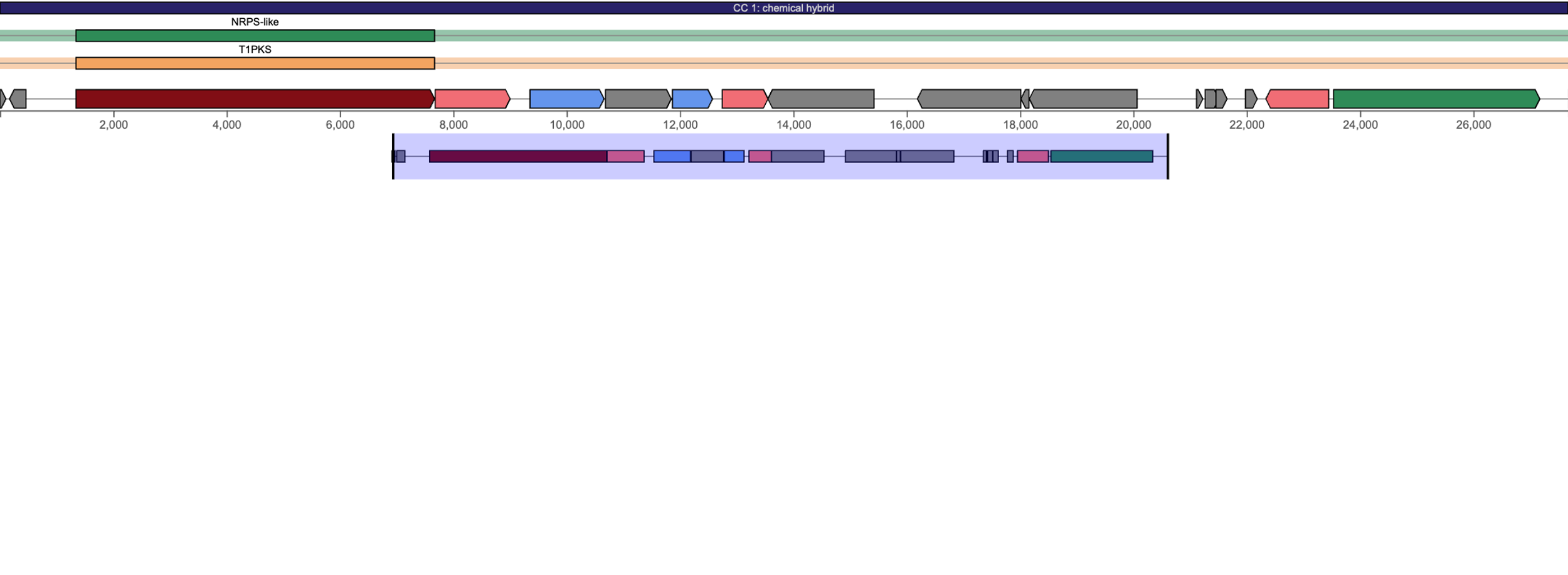
**

1. ***Microcystis aeruginosa* NIES-98_aeruginosin98-A, aeruginosin98-B, aeruginosin 98-C2**

**
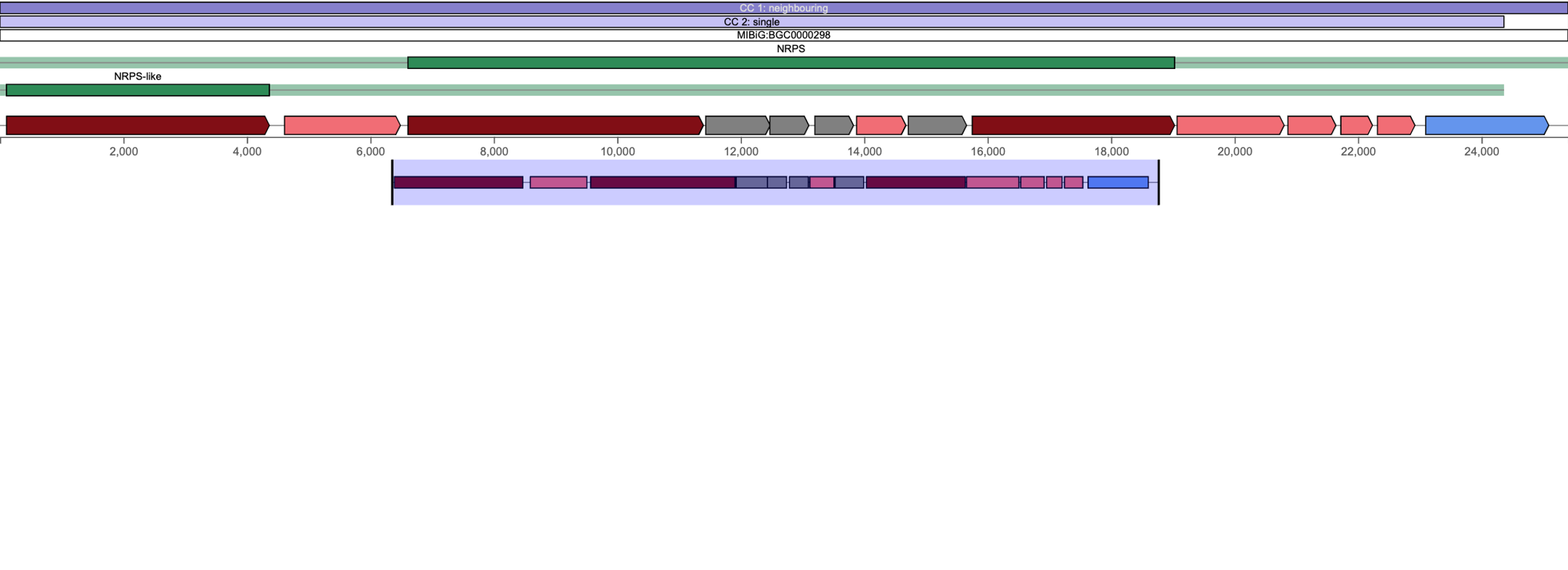
**

1. **Anabaenopeptin_16_0**

**
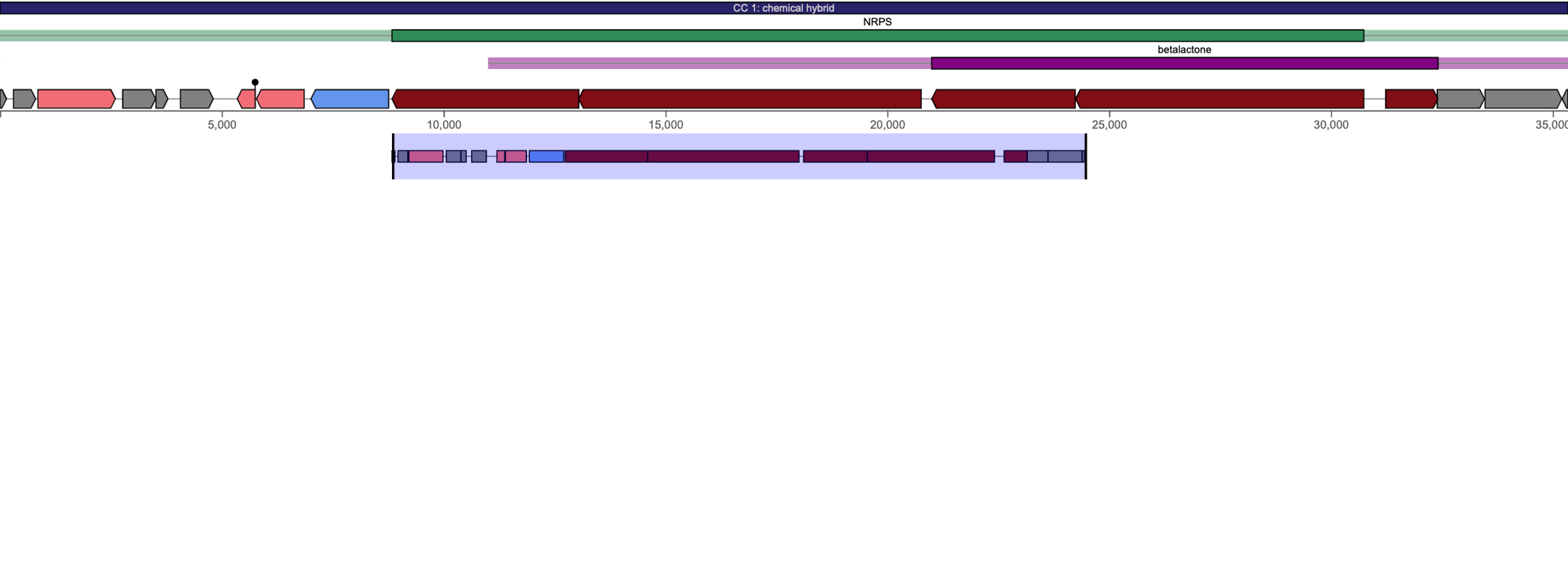
**

1. **Bartoloside-T1PKS_18_0**

**
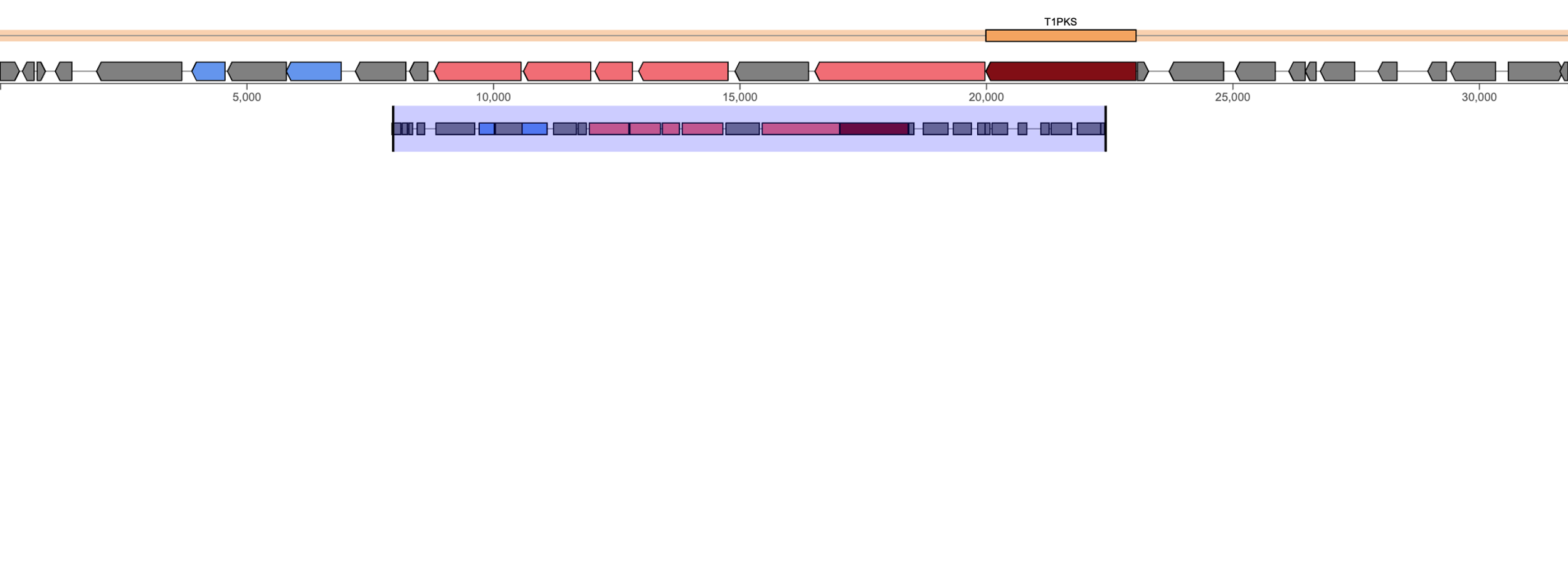
**

1. **Cyanobactin_50_0**

**
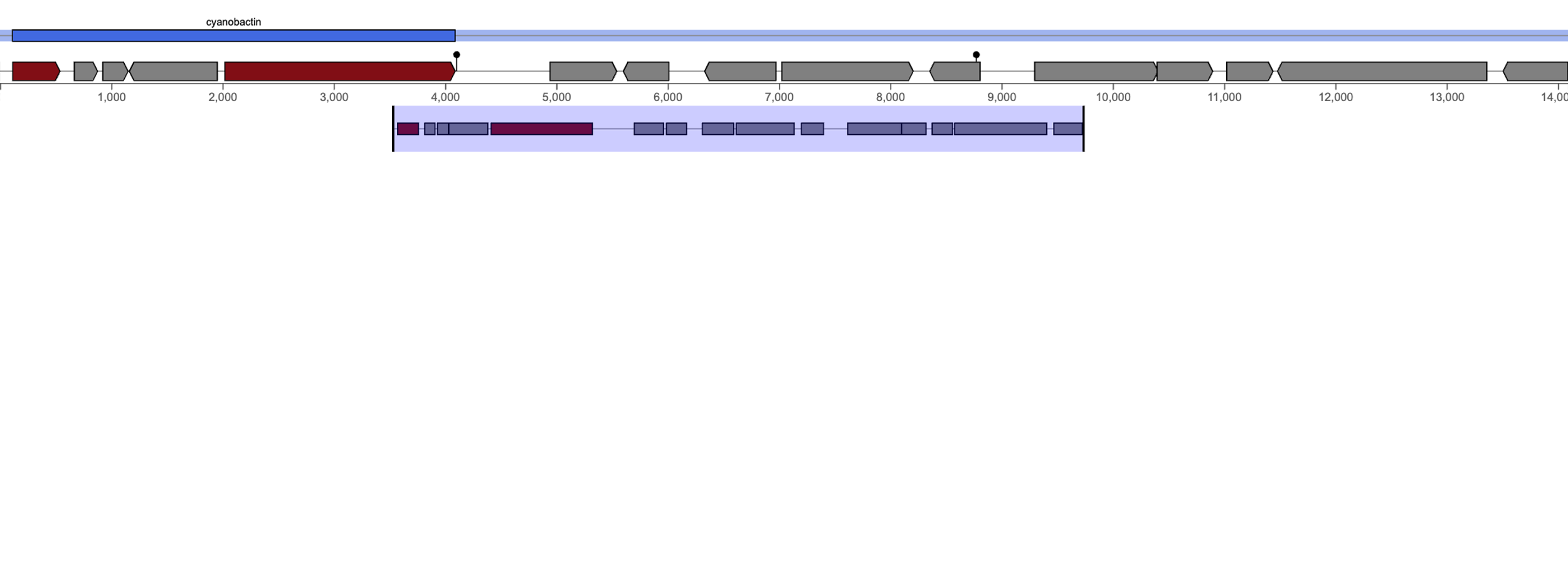
**

1. ***Microcystis* sp. NIVA-CYA172/5_cyanopeptolin**

**
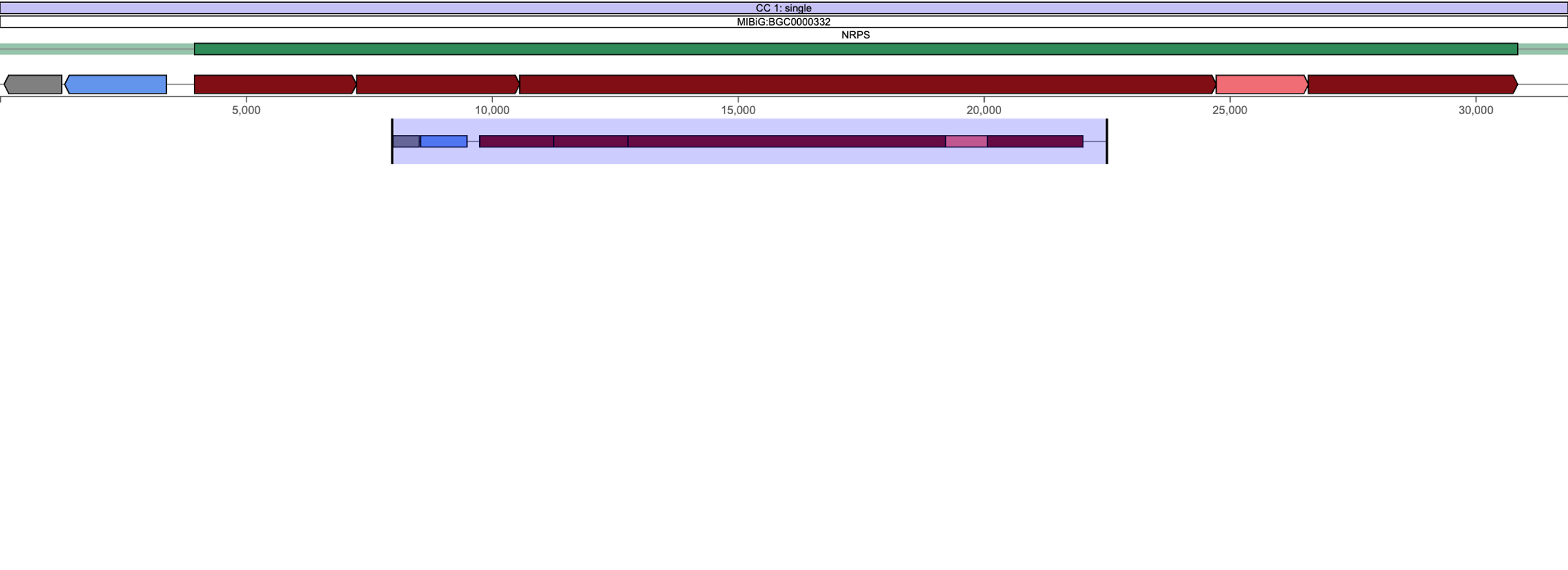
**

1. ***Microcystis aeruginosa* NIES-87_kasumigamide**

**
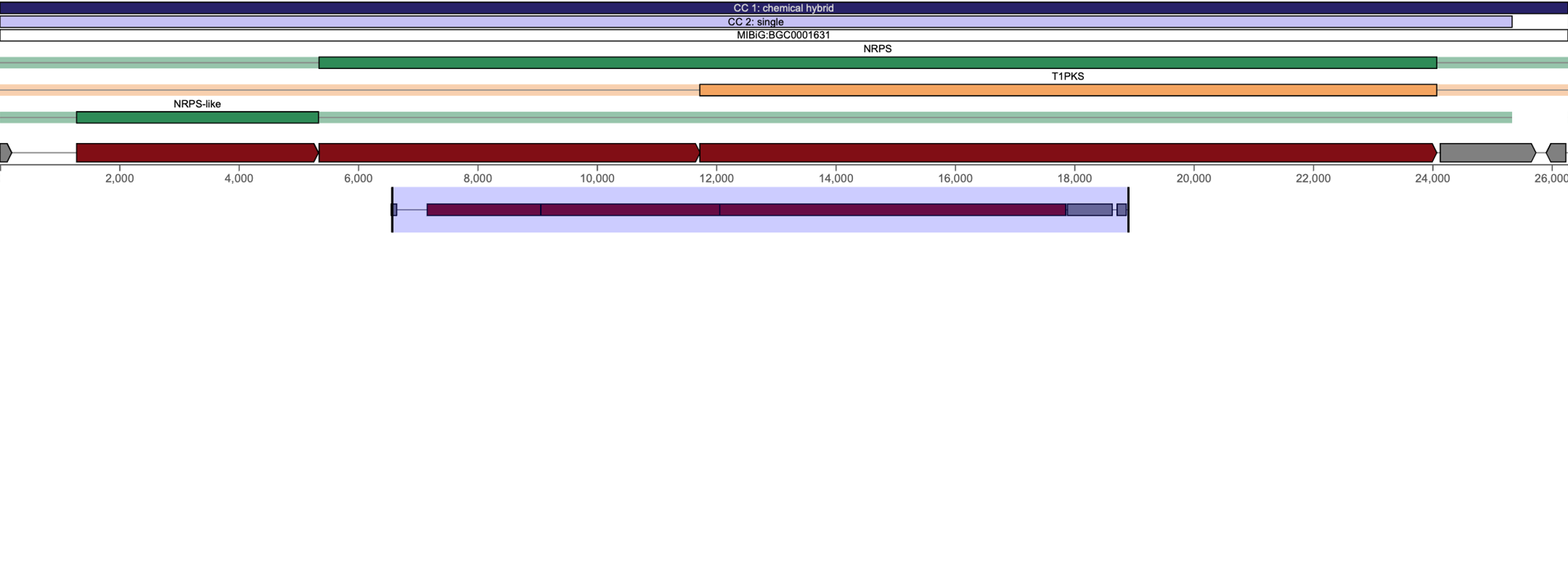
**

1. **RRE-containing_23_0**

**
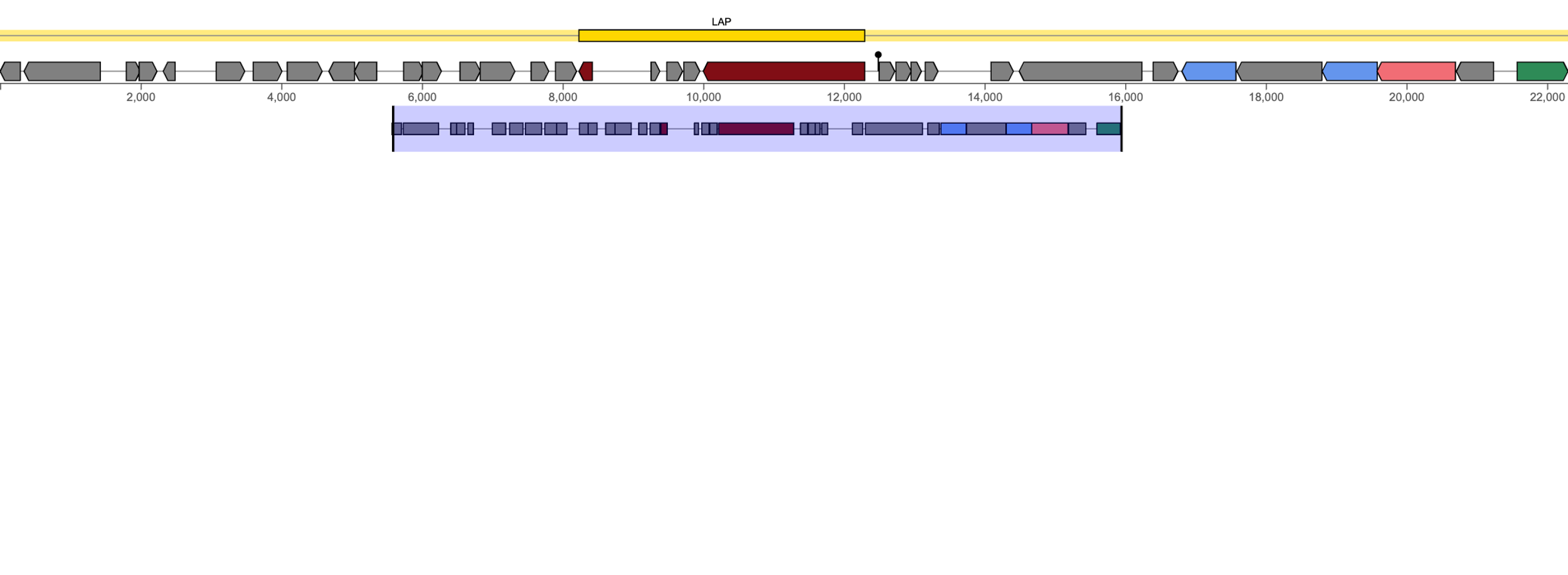
**

1. **lanthipeptide_21_0**

**
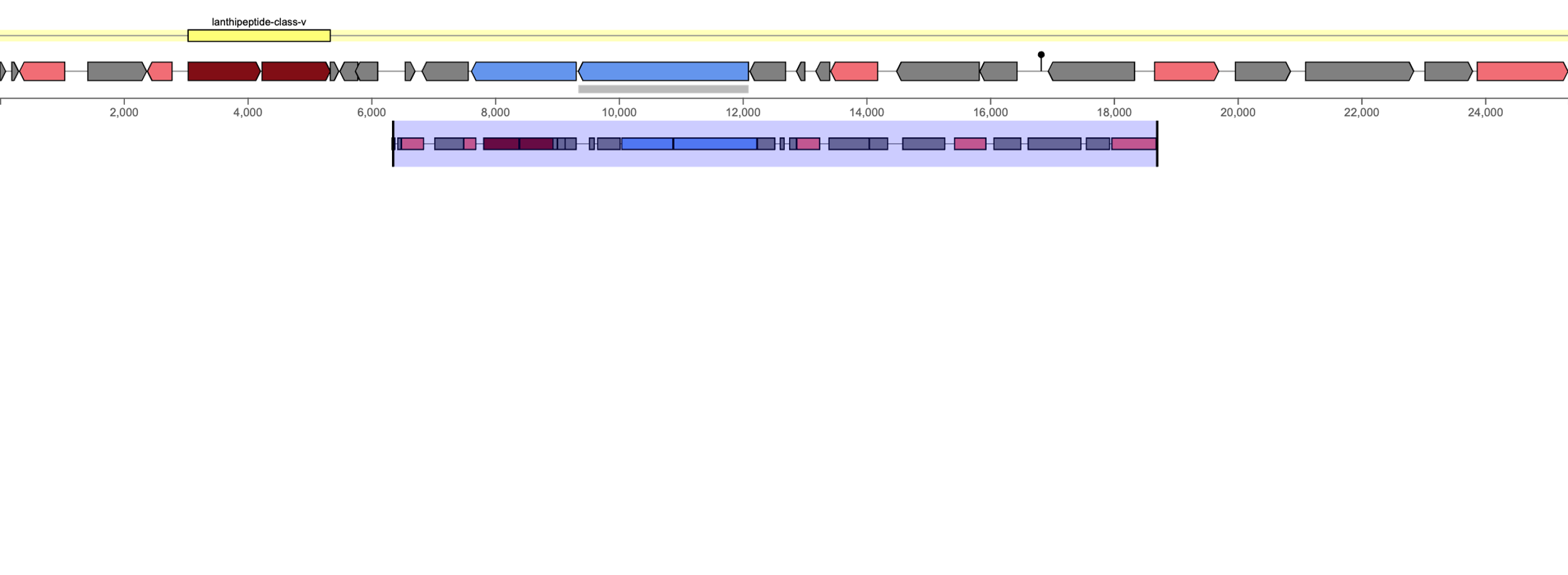
**

1. ***Microcystis aeruginosa* PCC7806_microcystin LR**

**
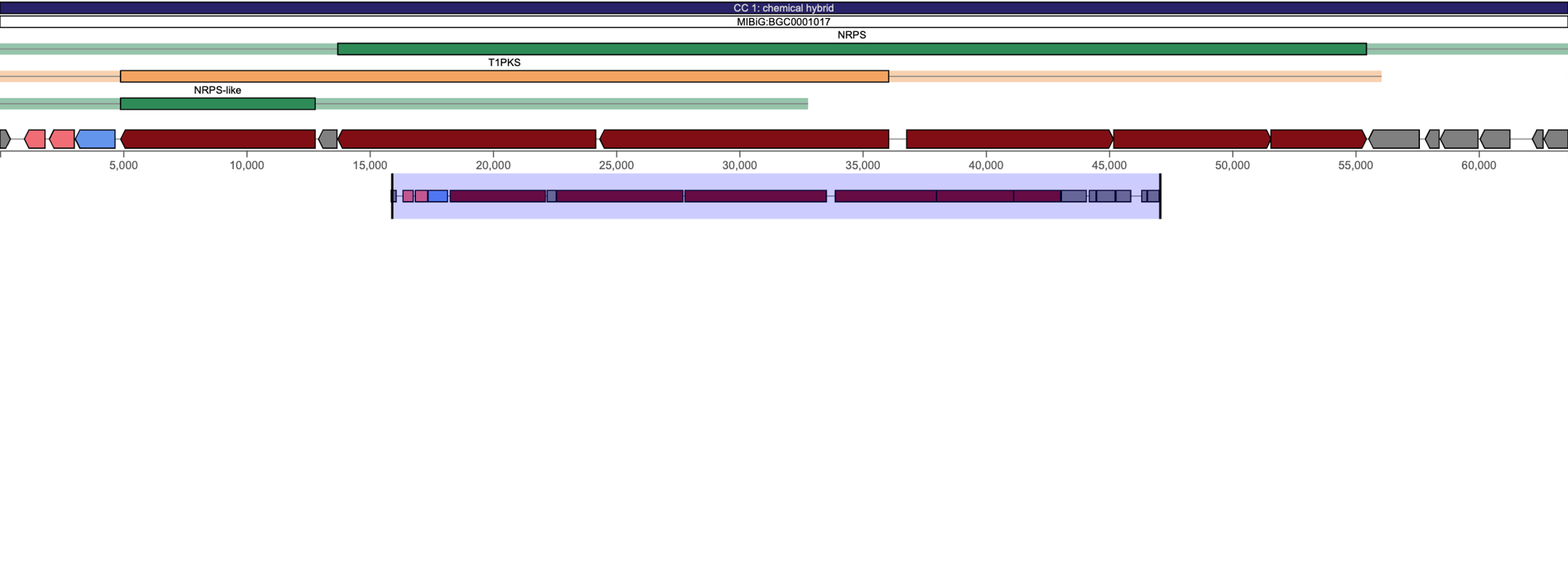
**

1. ***Microcystis aeruginosa* LEGE91341_microginin**

**
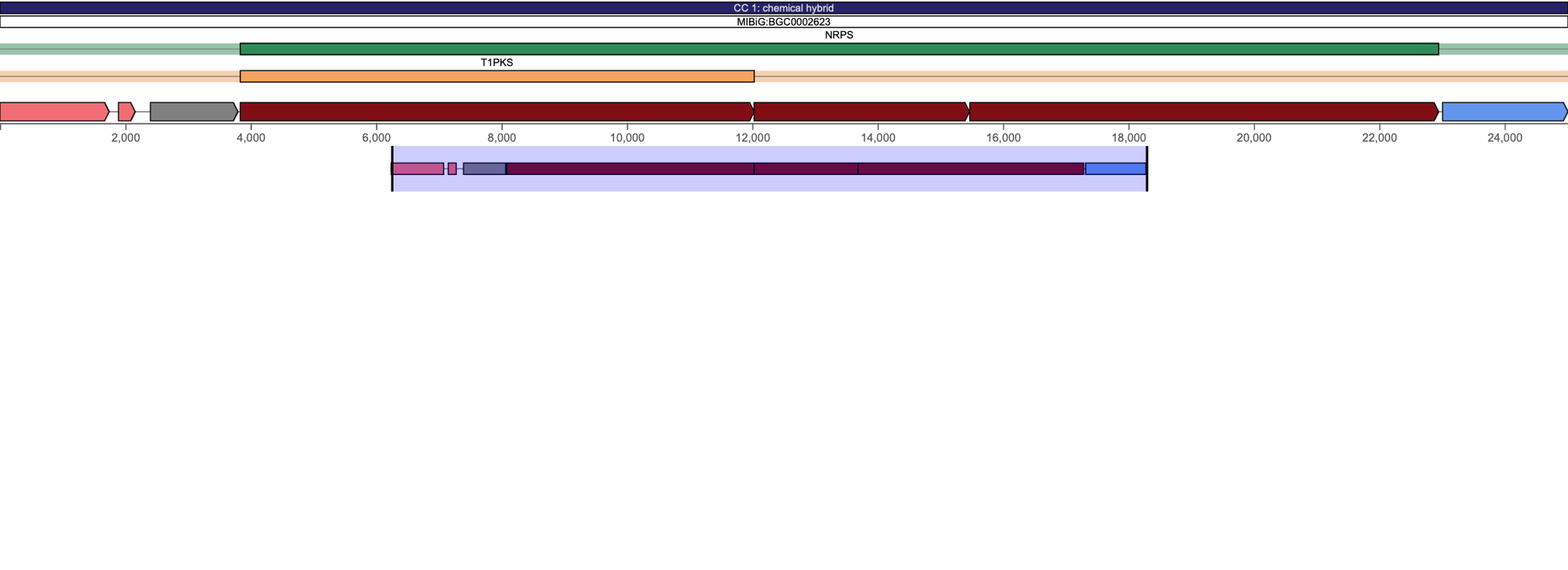
**

1. ***Microcystis aeruginosa* NIES-298_microviridinB**

**
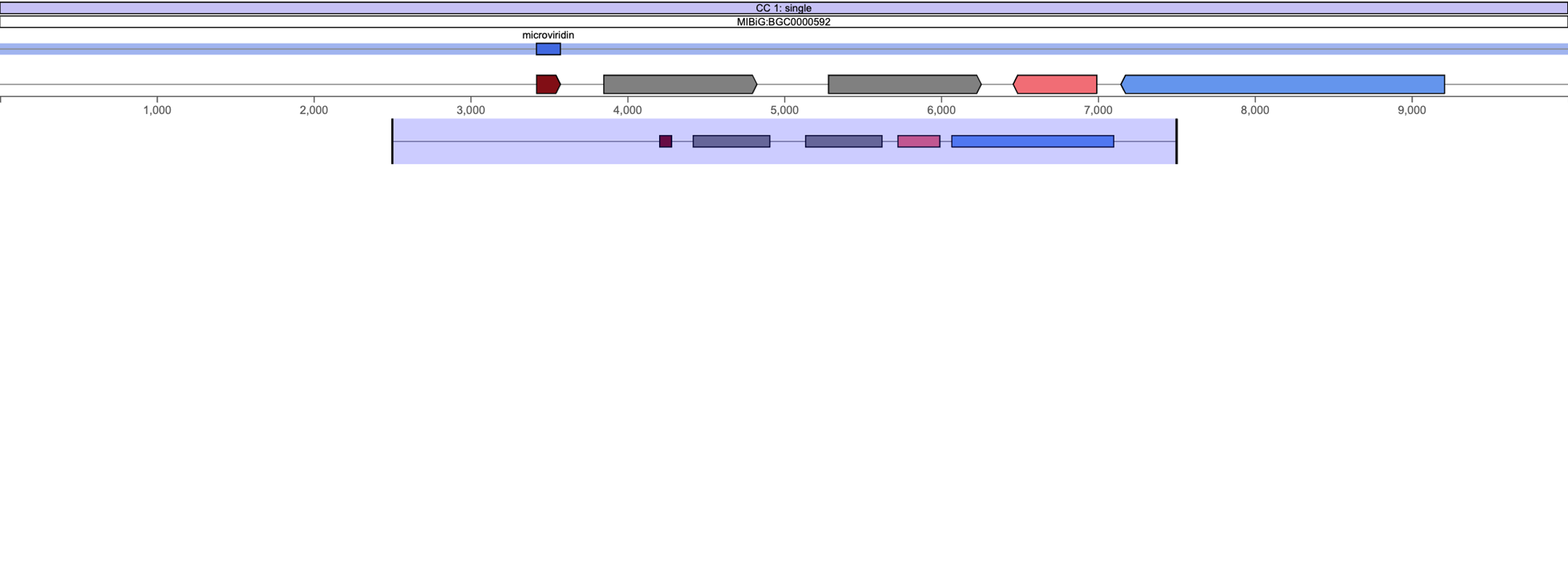
**

1. **NRPS-T1PKS_26_0**

**
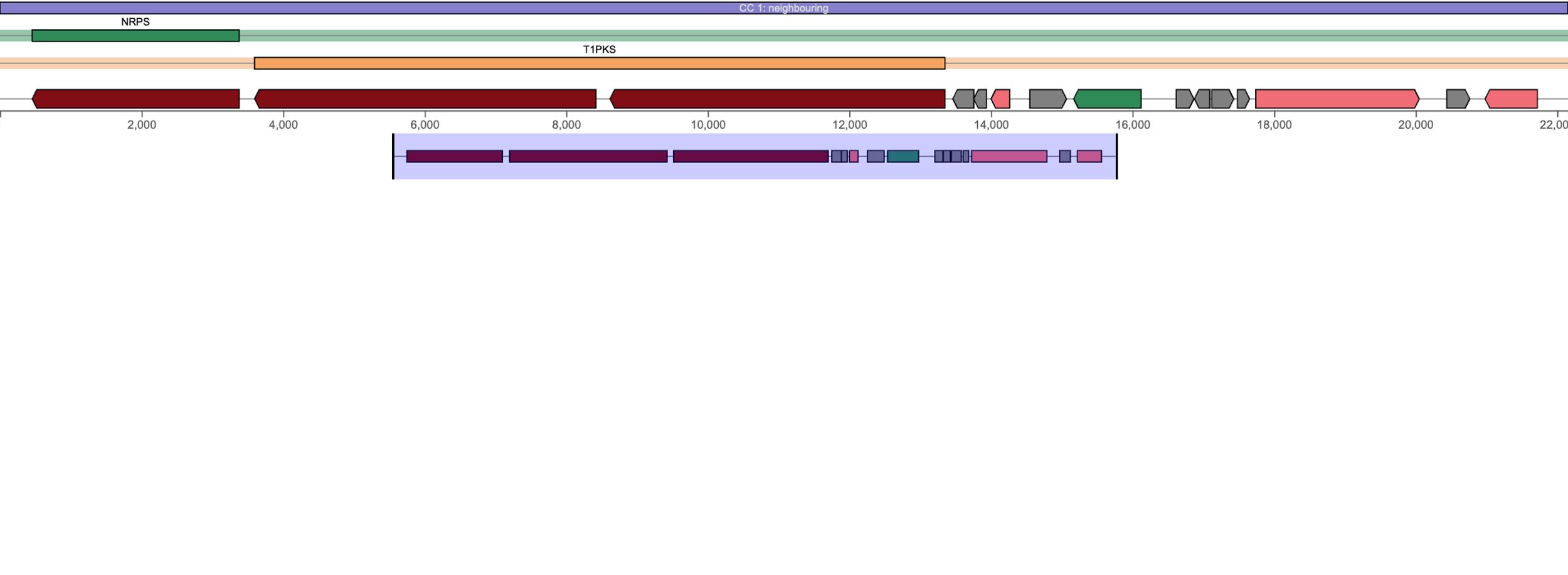
**

1. **NRPS-like_34_0**

**
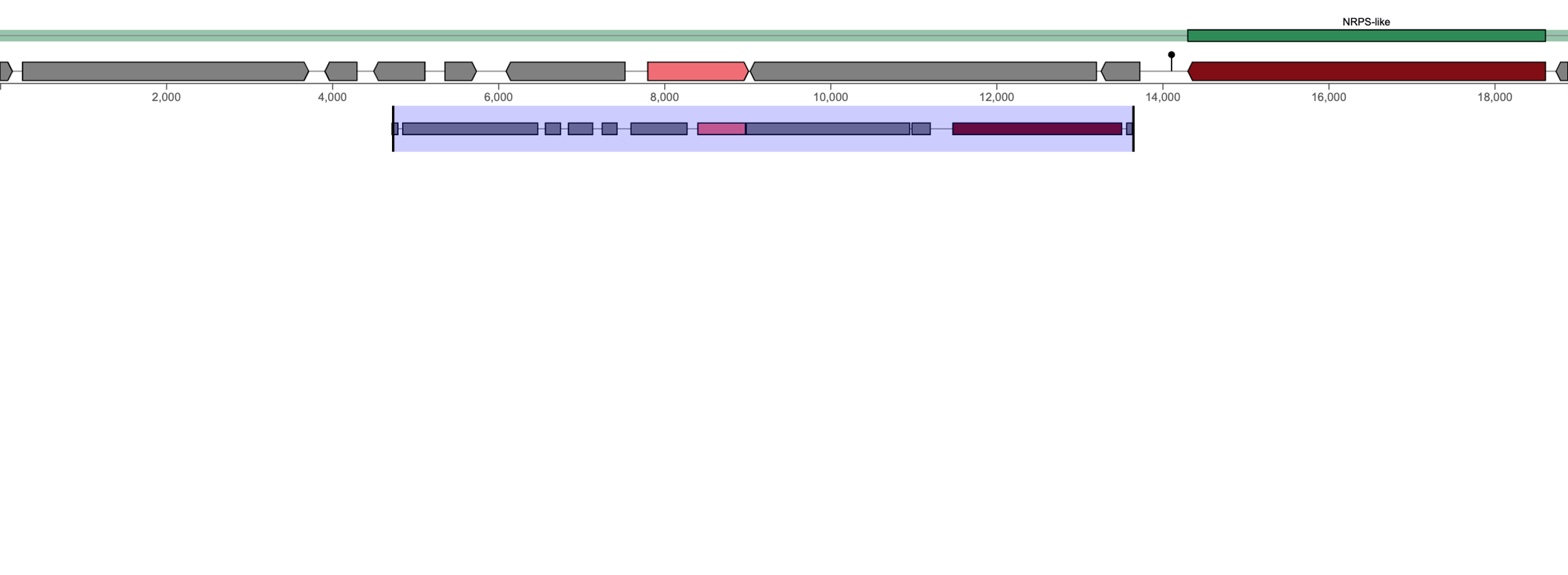
**

1. **T3PKS-Merocyclophane_5_0**

**
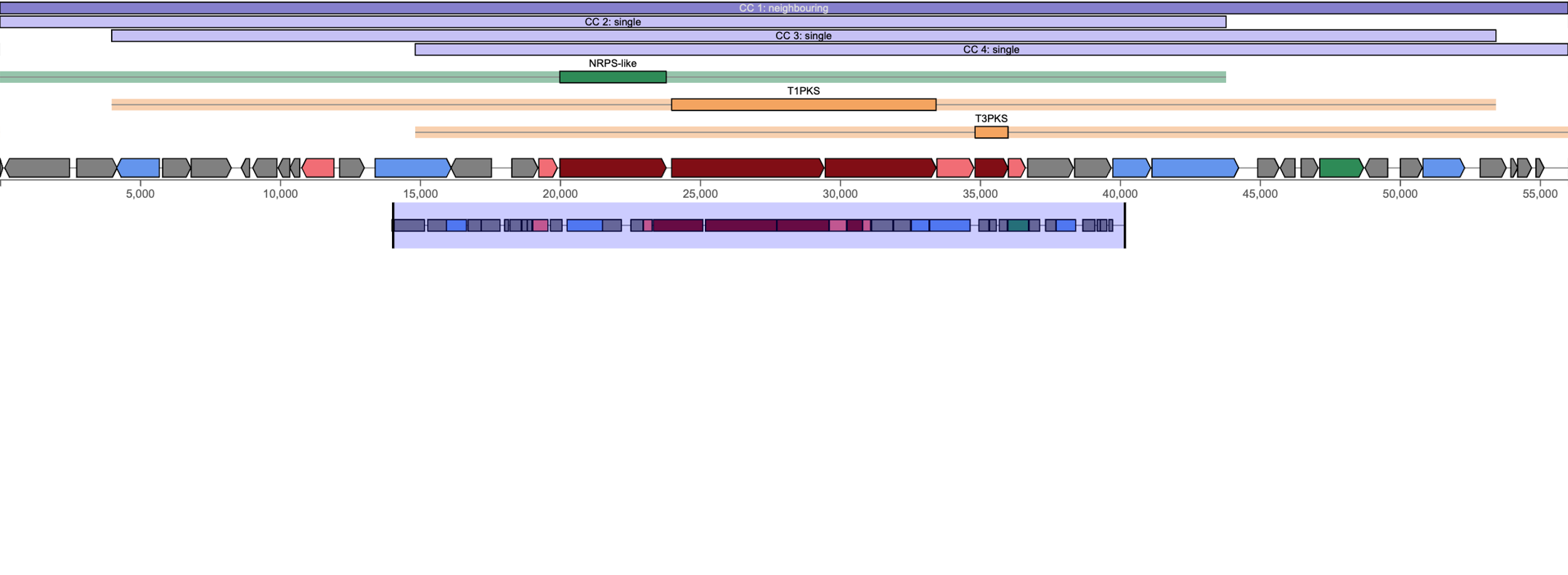
**

1. **T1PKS-Terpene_11_0**

**
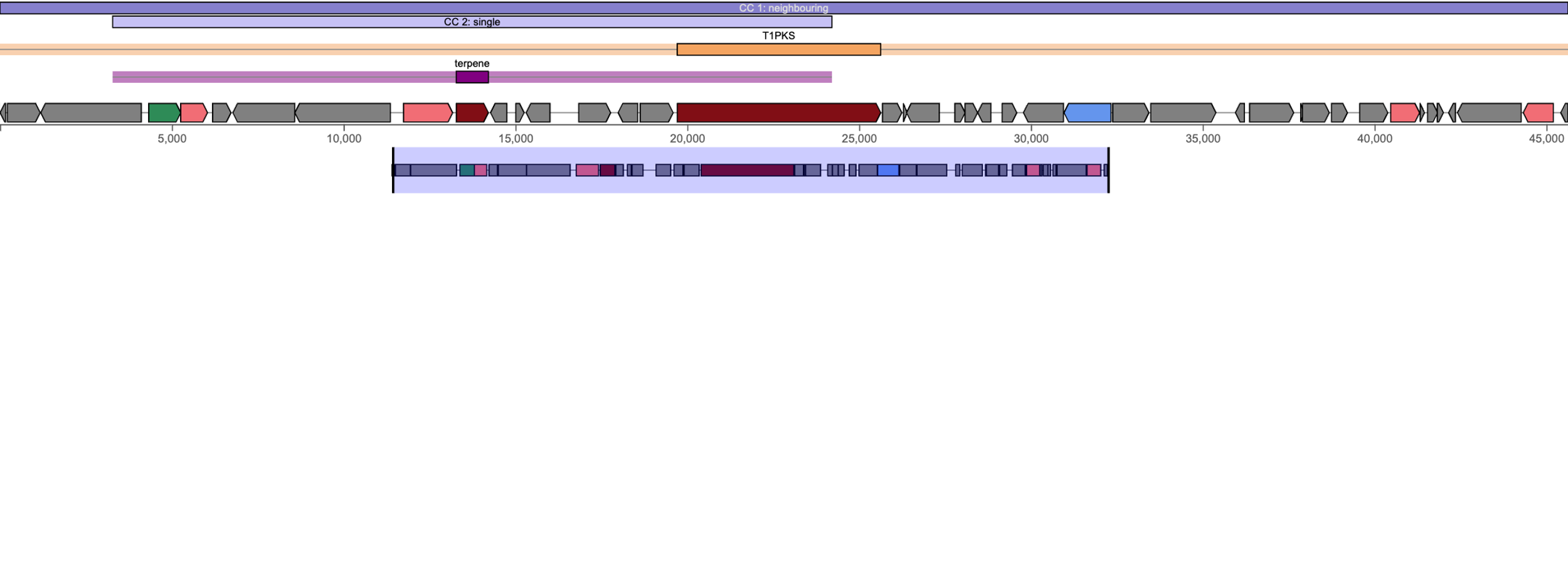
**

1. **Cyanobactin_90_0**

**
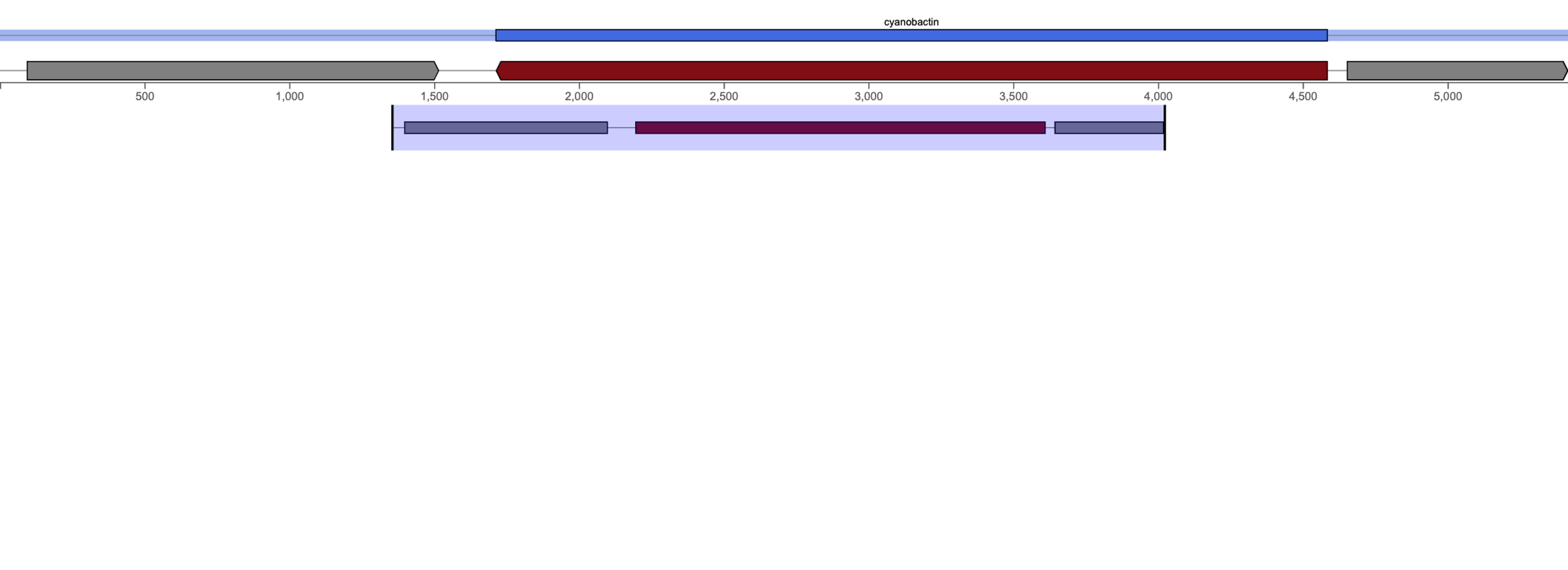
**

1. **Cyanobactin_64_0 (Cyanobactin 2)**

**
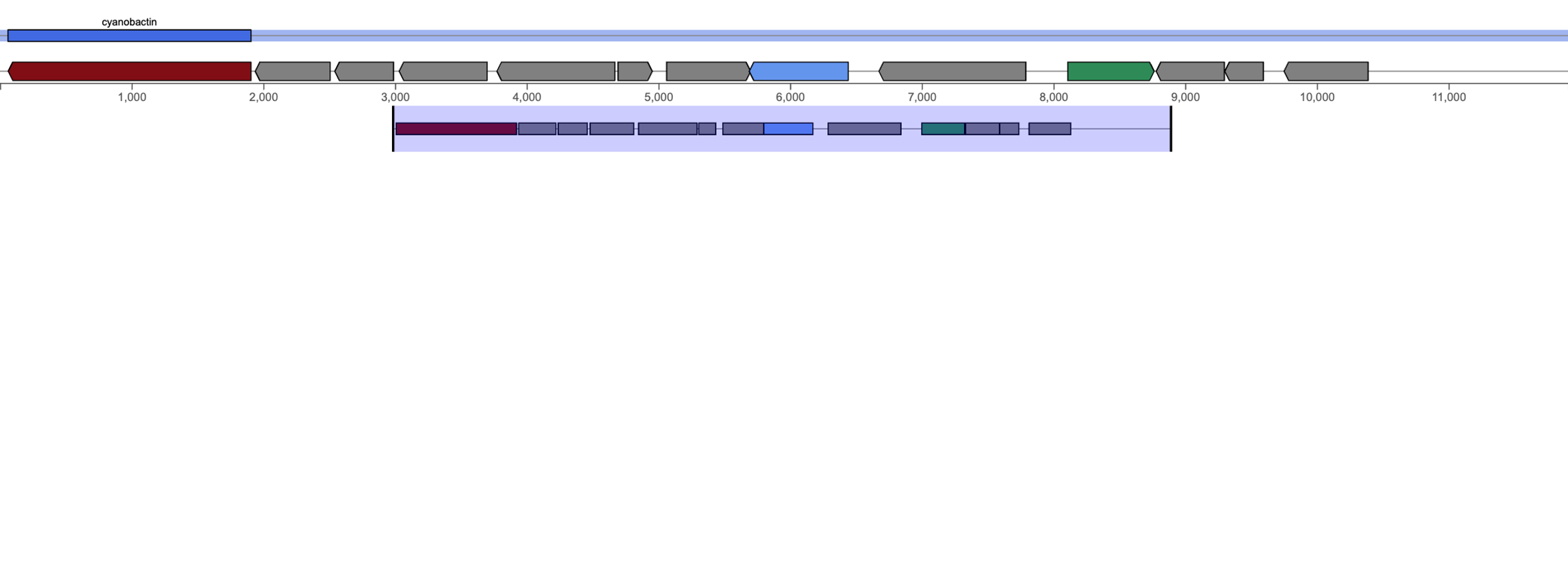
**

1. ***Microcystis aeruginosa* PCC7005_piricyclamide7005E1, piricyclamide7005E2, piricyclamide7005E3, piricyclamide7005E4**

**
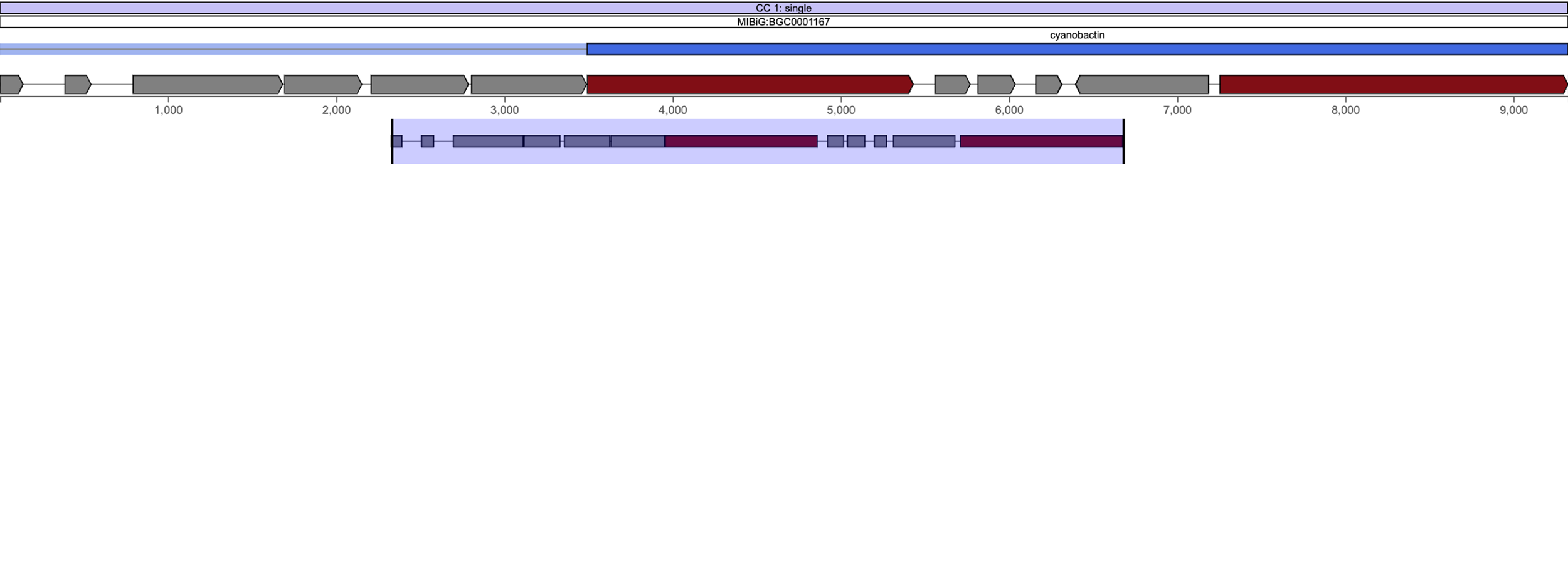
**

1. **T1PKS_37_0**

**
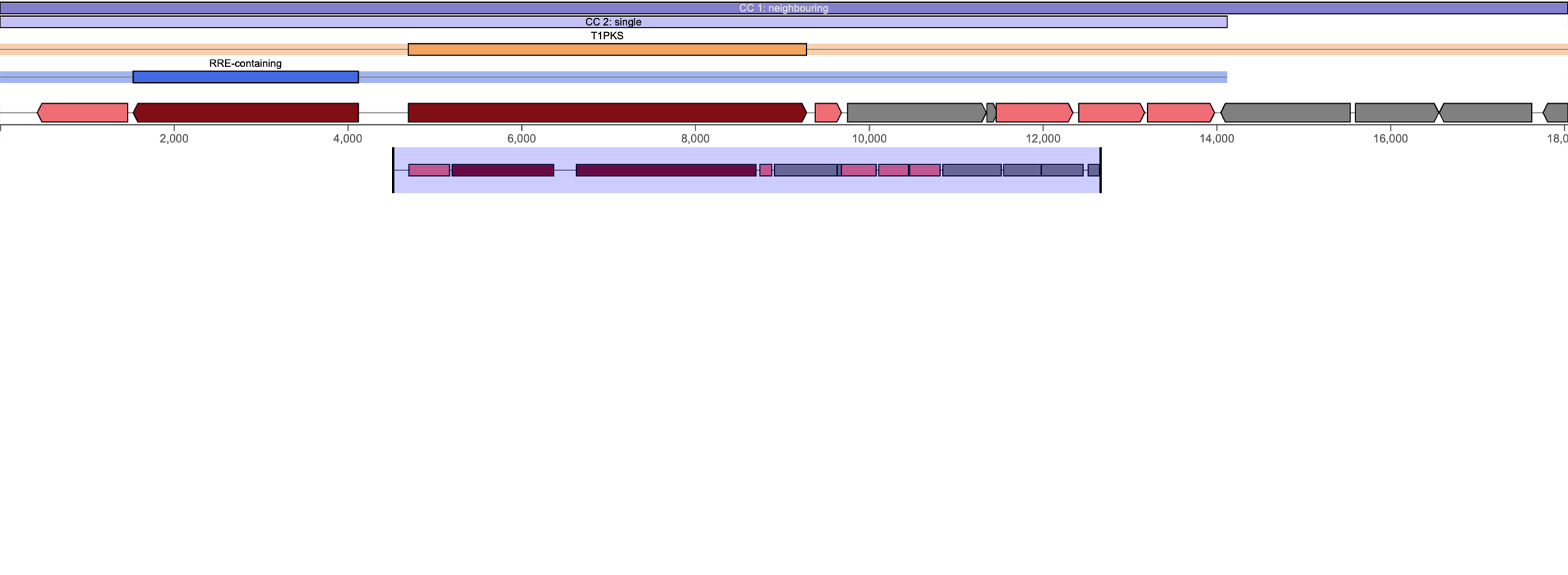
**

1. **Novel-T3PKS_28_0**

**
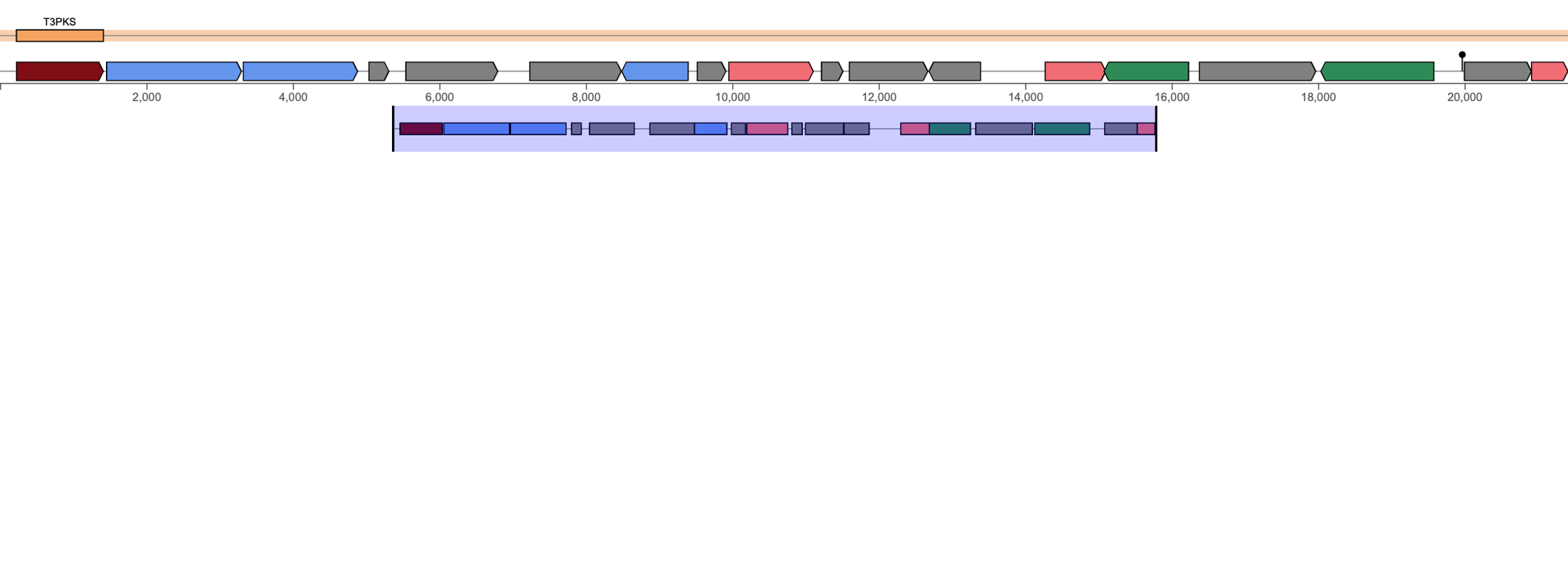
**

1. **hglE-KS_45_0**

**
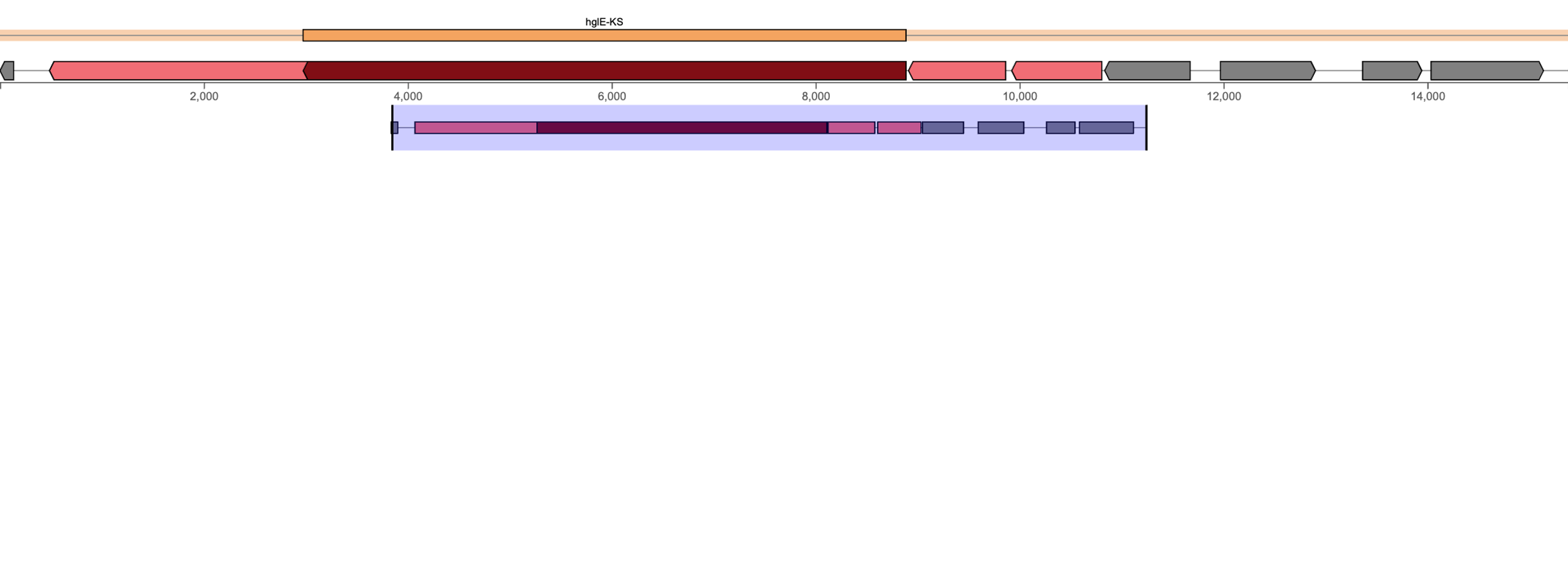
**

1. **RiPP-like_84_0**

**
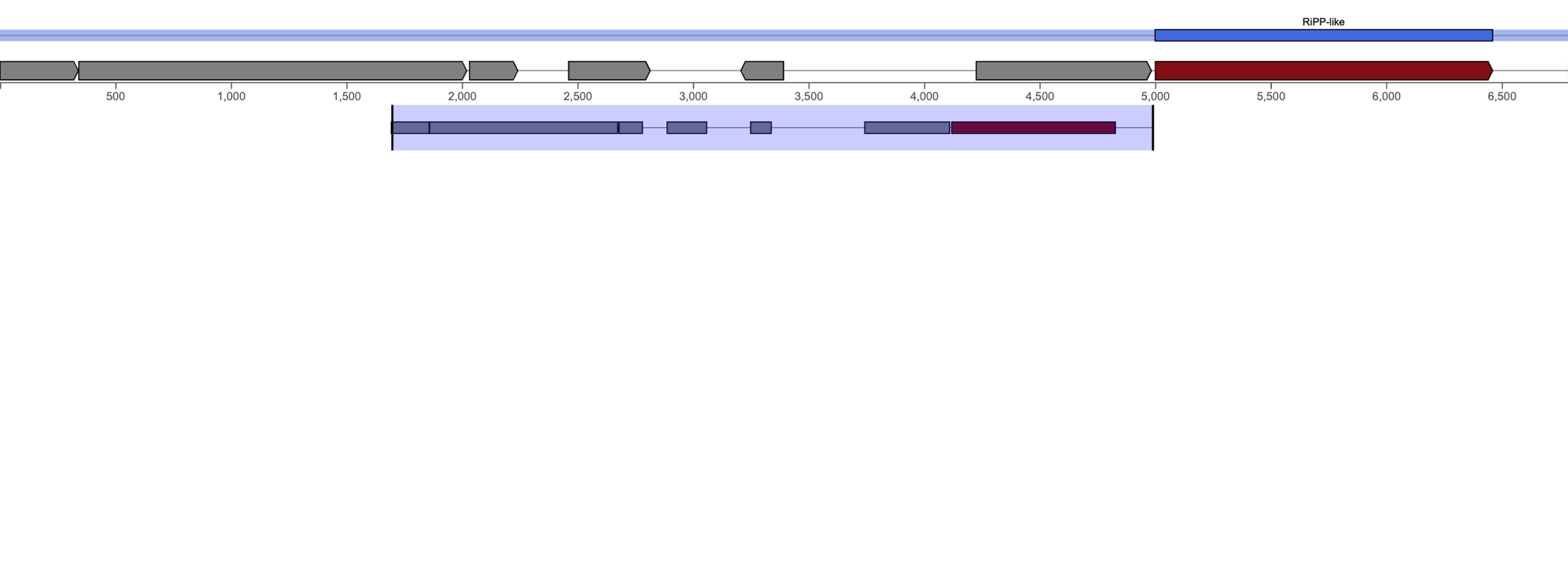
**

1. **Anabaena sp.90_anacyclamide A10**

**
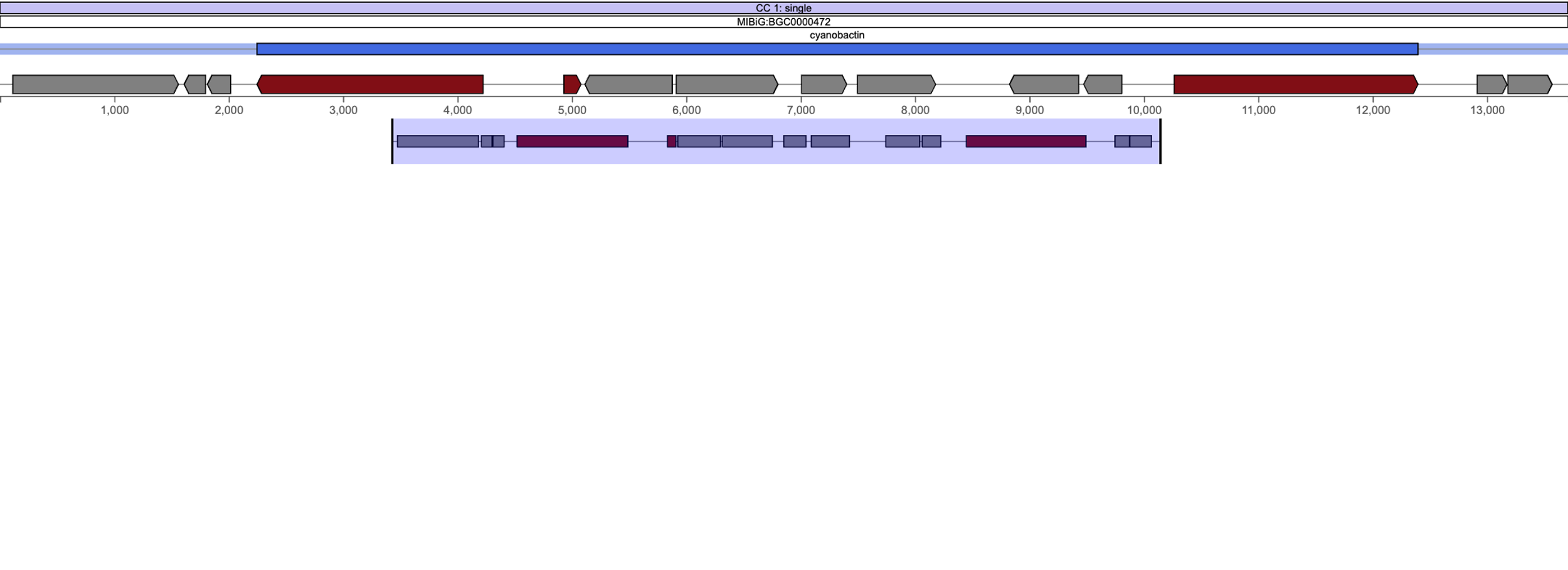
**

1. **samp_2073_477589_LAP**

**
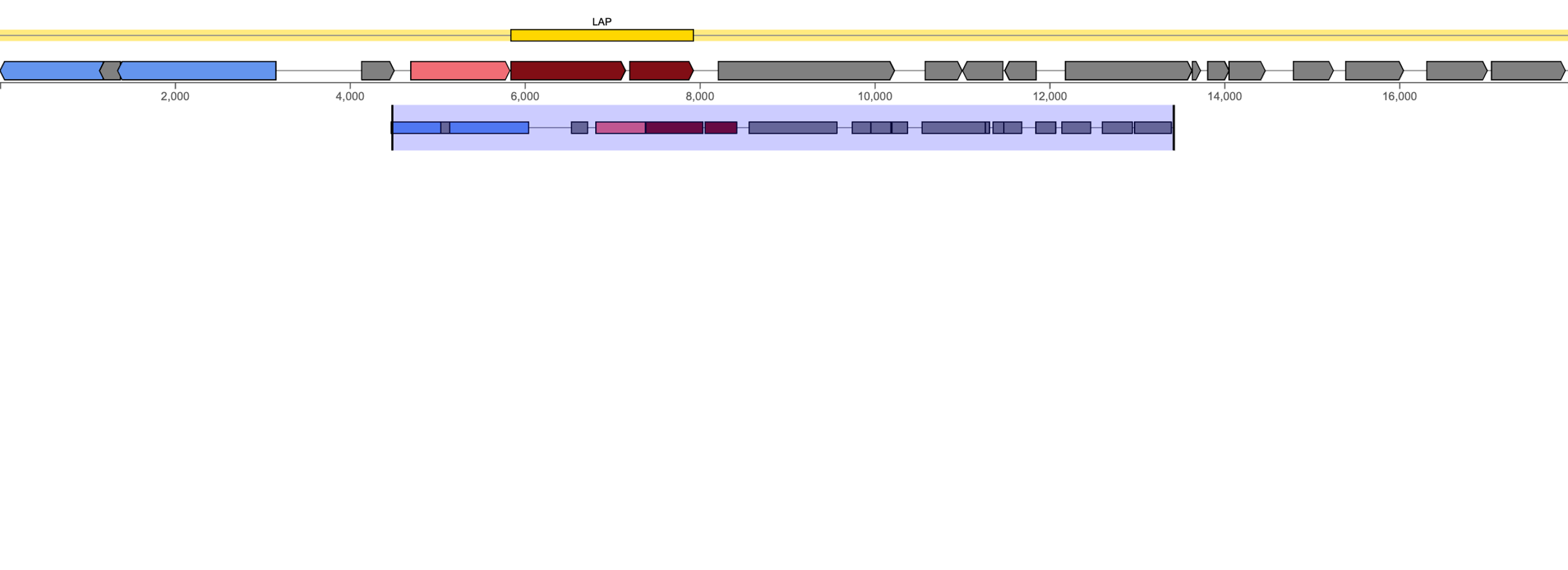
**

1. **samp_471_14978_NRPS**

**
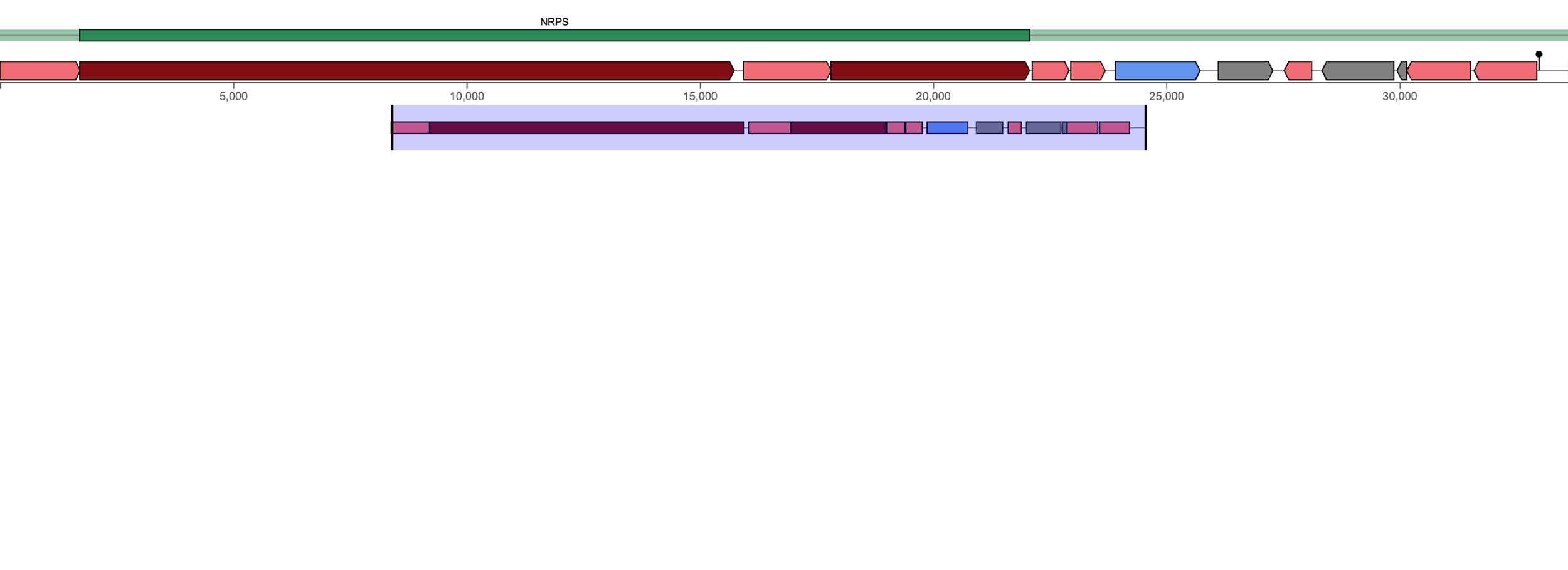
**

1. ***Dolichospermum circinale* AWQC131C_decarbamoylgonyautoxin 2, decarbamoylgonyautoxin 3, decarbamoylsaxitoxin, gonyautoxin 2, gonyautoxin 3**

**
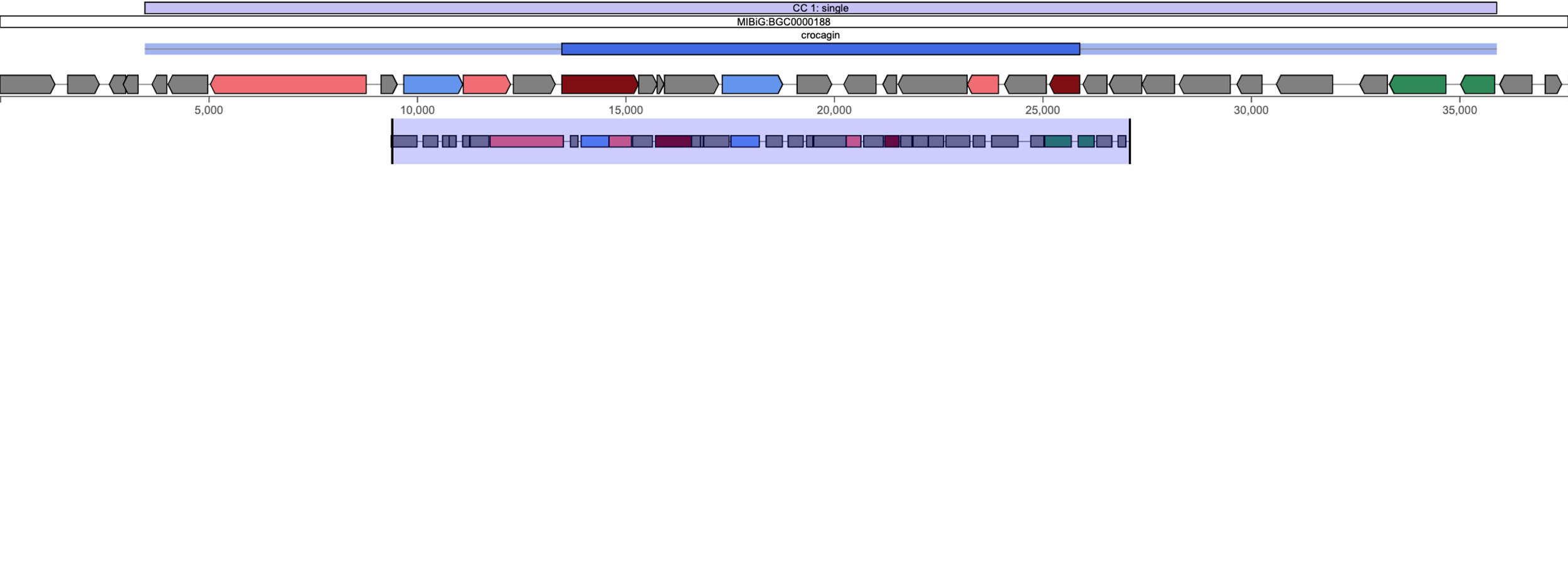
**

1. ***Microcystis aeruginosa* PCC7806_aerucyclamide/microcyclamide**

**
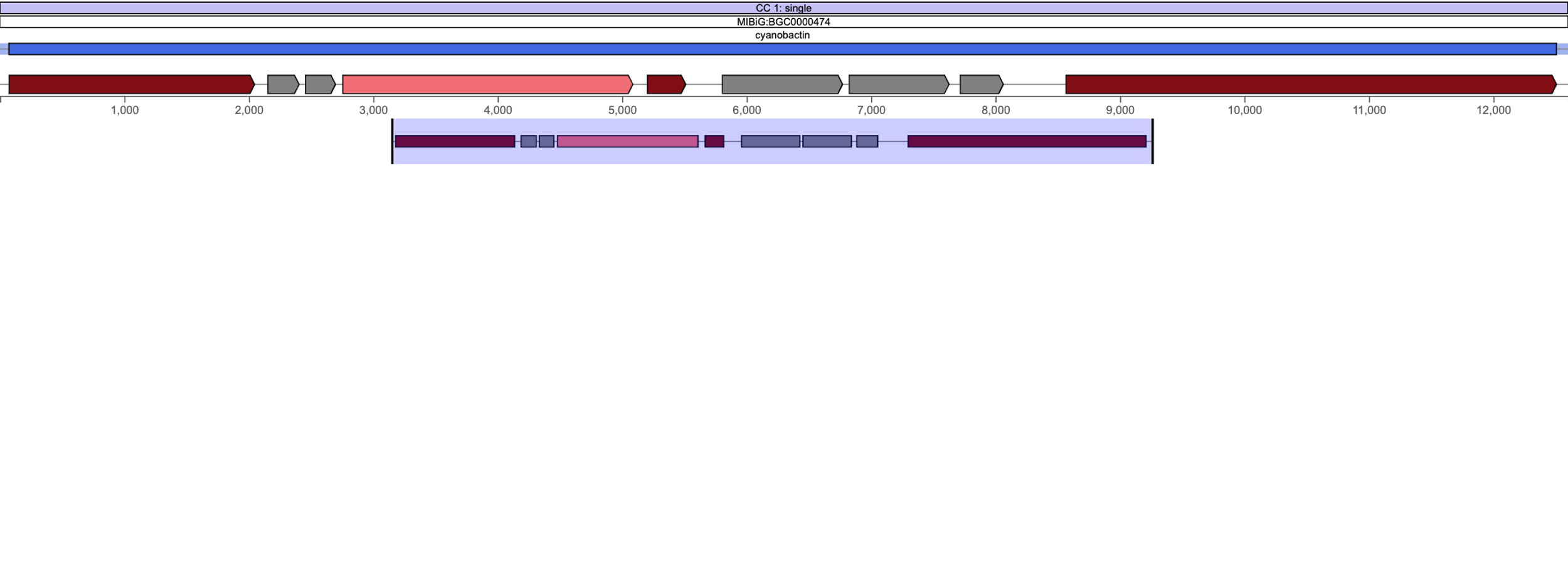
**

**
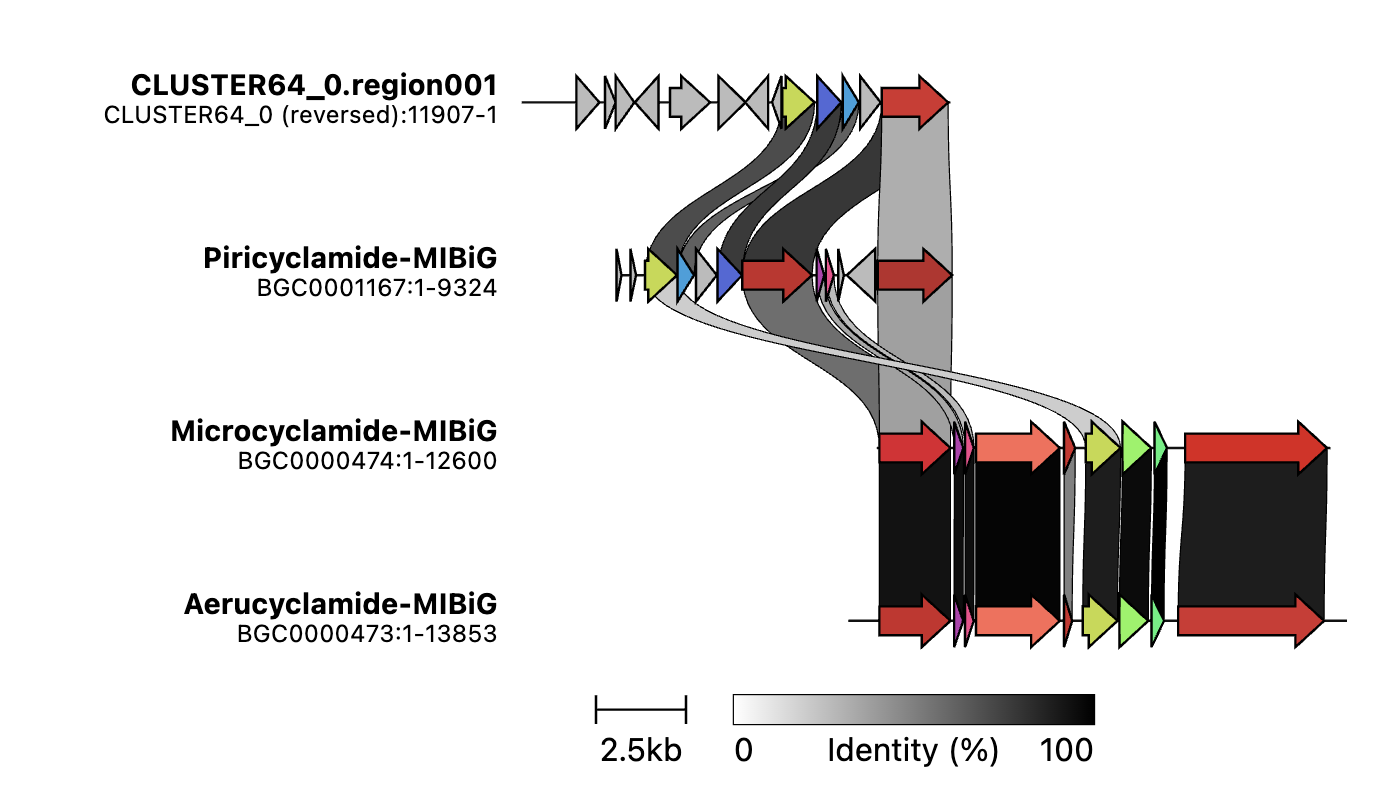
**

1. **samp_4305_693378_NRPS**

**
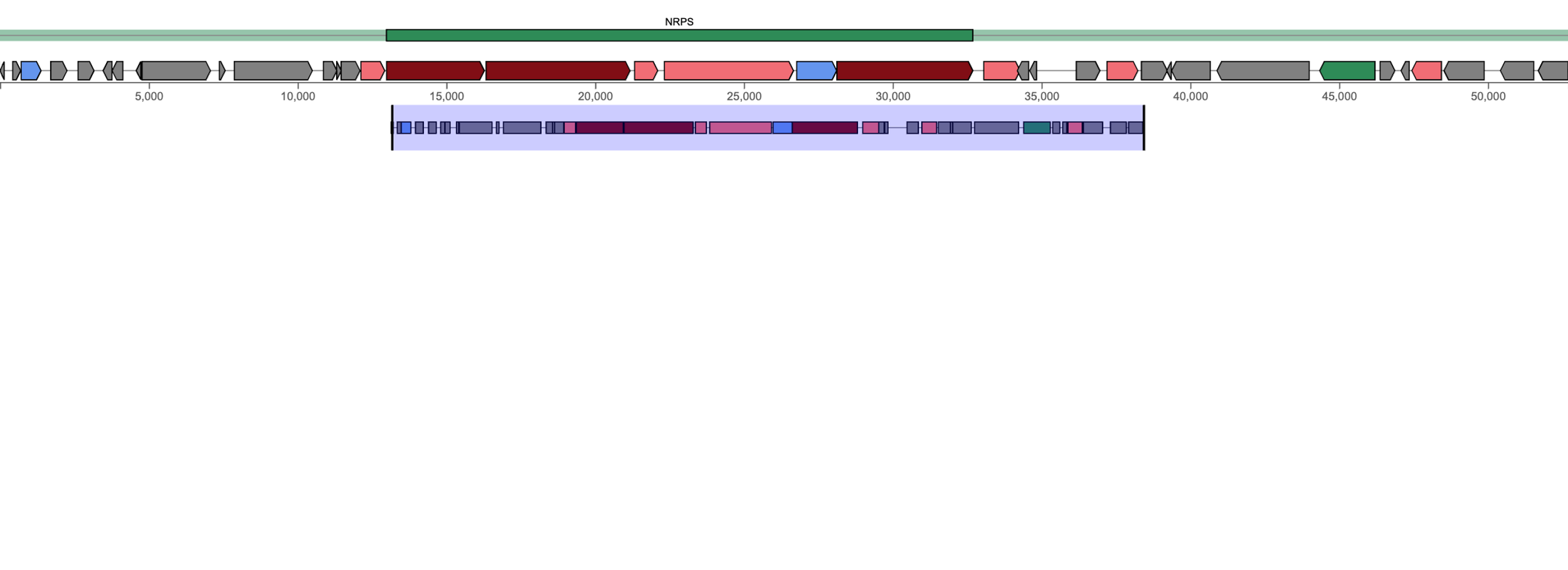
**

1. **RiPP-like_80_0**

**
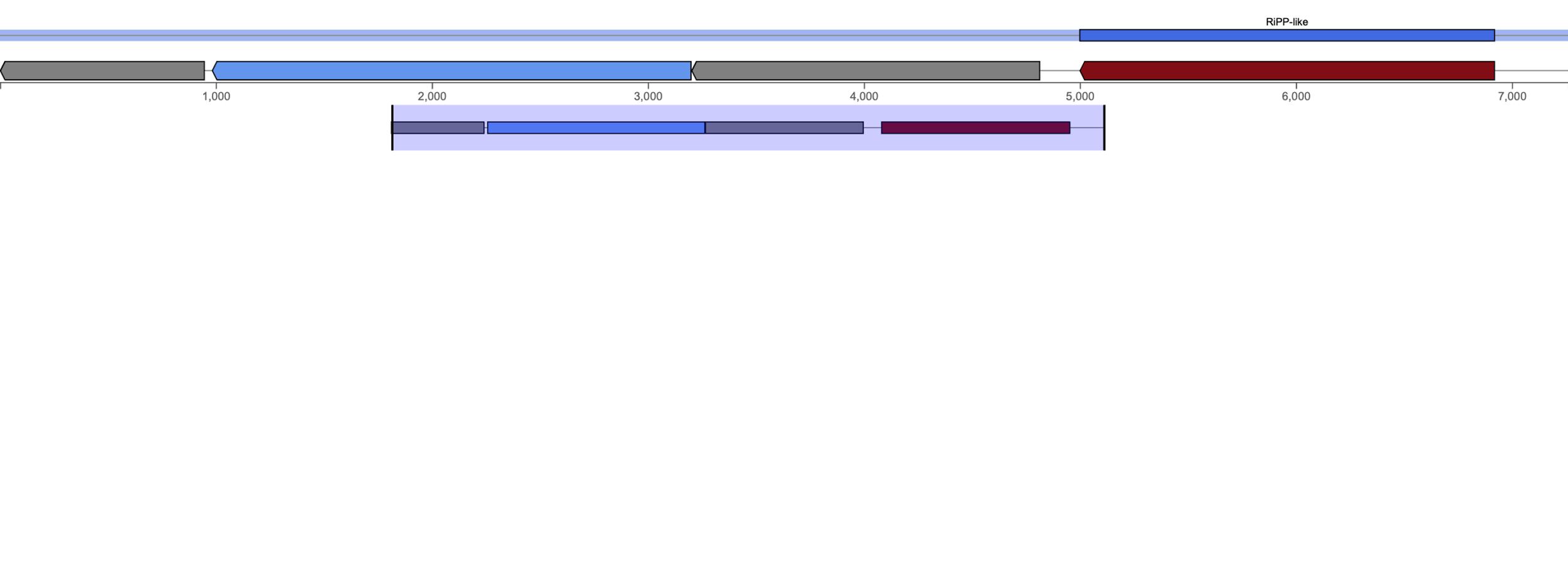
**

1. **lanthipeptide_87_0**

**
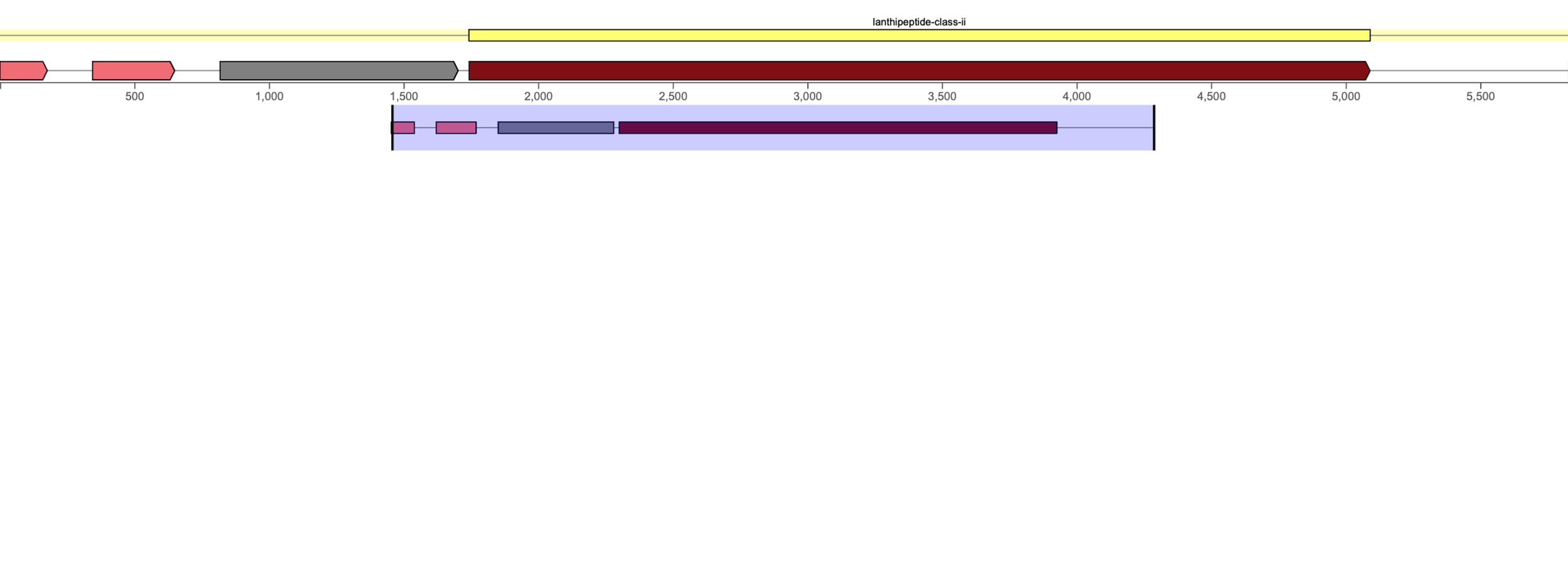
**

1. **lanthipeptide_54_0**

**
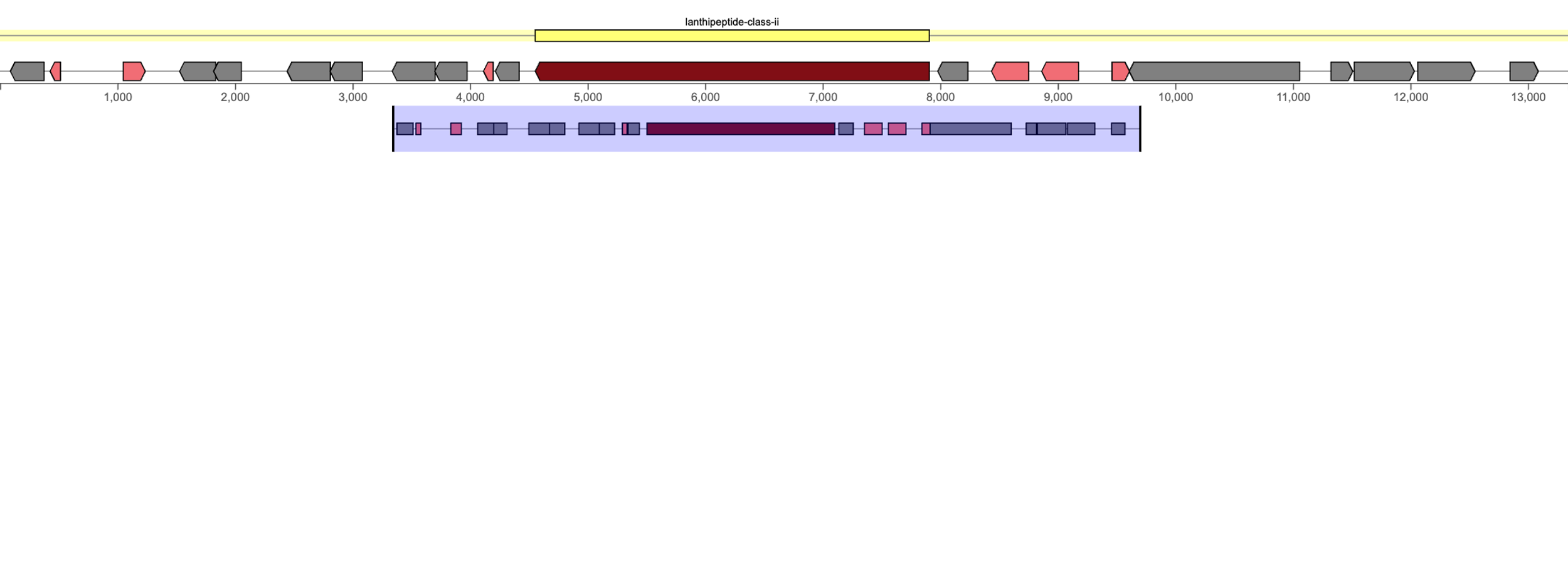
**

1. **samp_4305_474408_T1PKS**

**
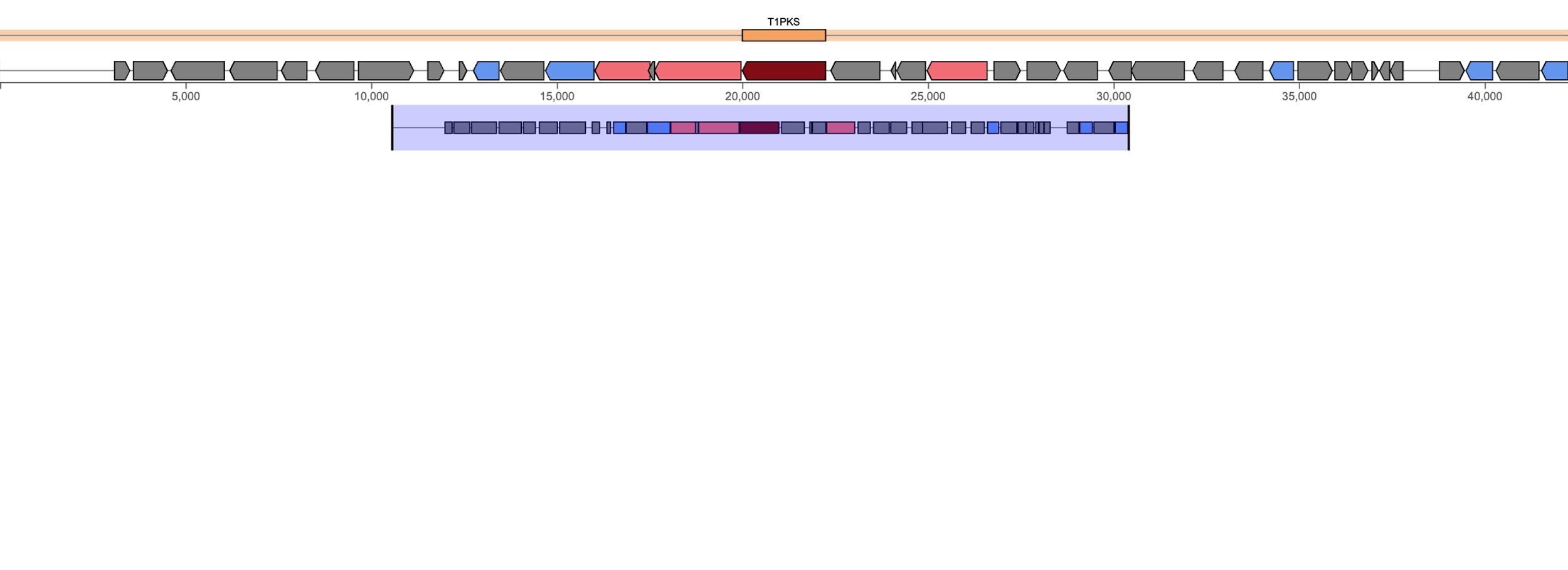
**

1. **T1PKS_51_0**

**
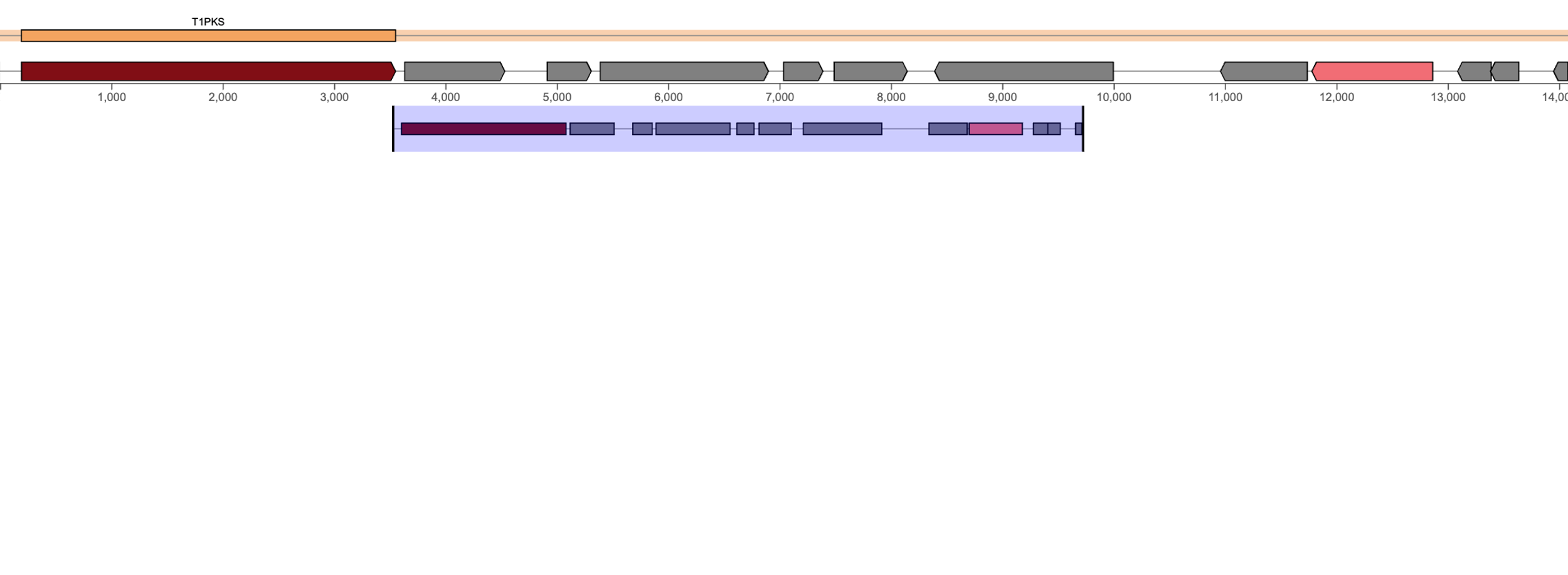
**

1. **samp_4380_82358_NRPS-like**

**
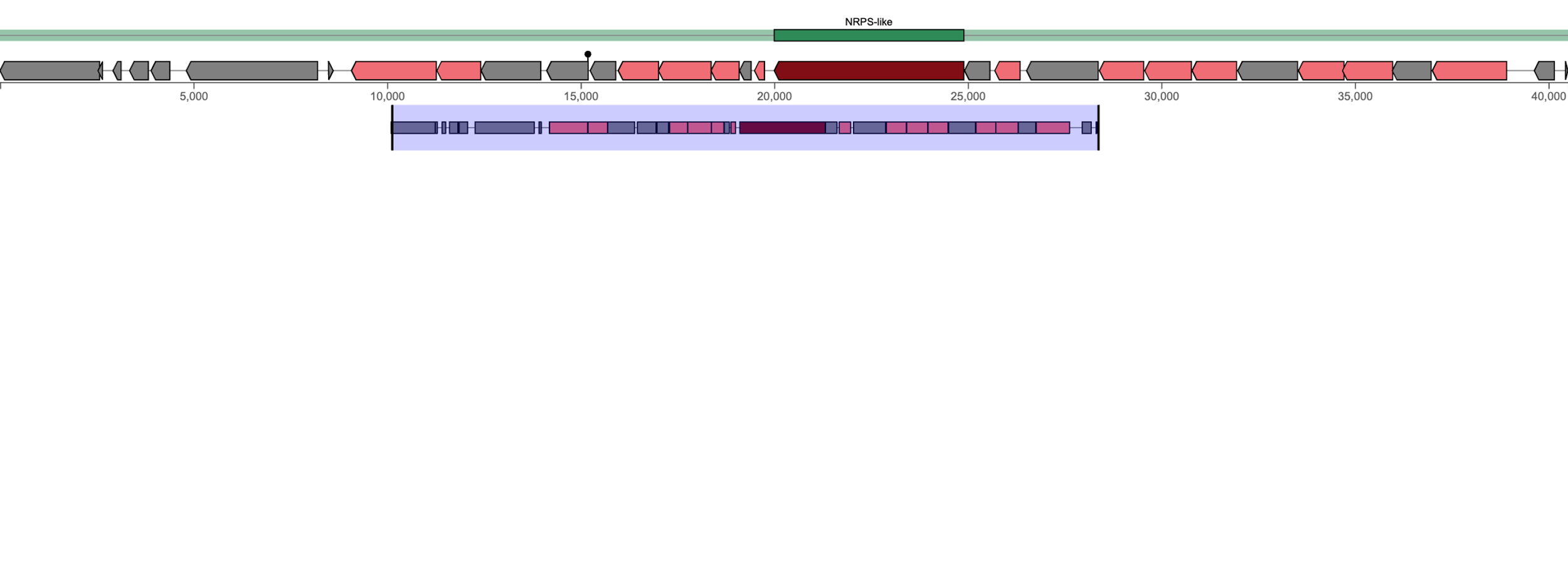
**

1. **samp_4305_554341_lanthipeptide-class-v**

**
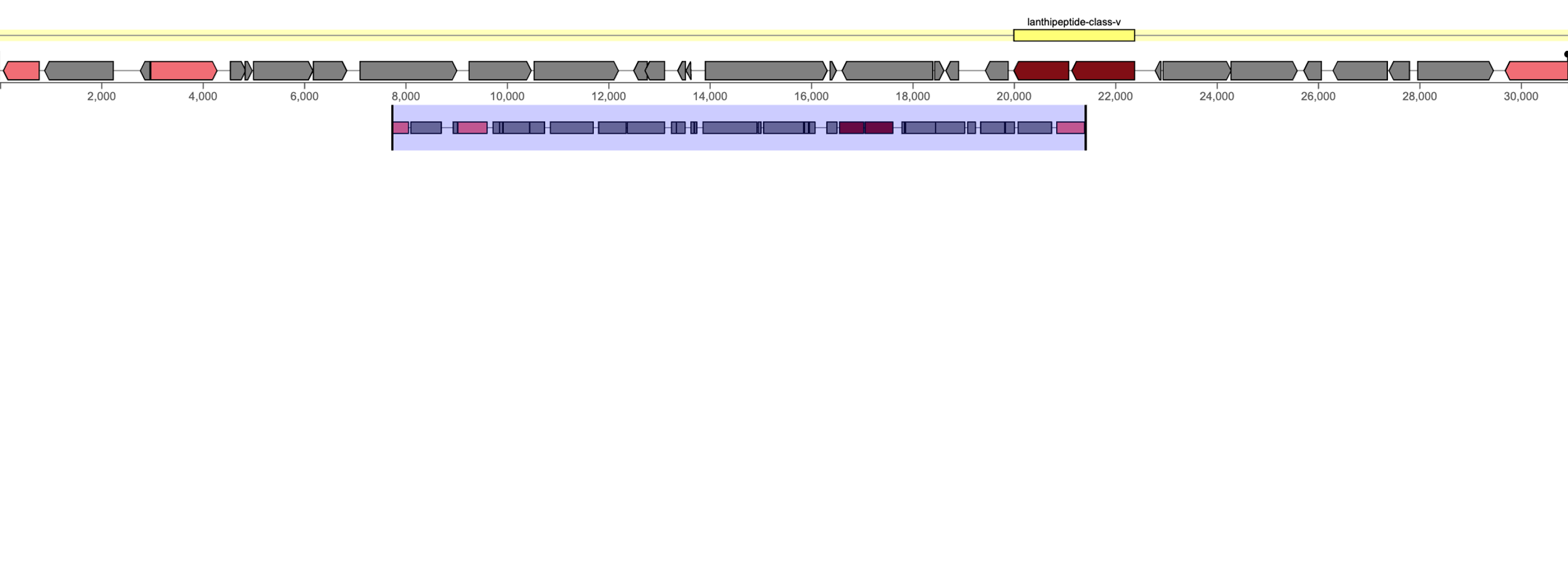
**

**SI Figure 2** (a-jj): Schematics of biosynthetic gene clusters used in the study, generated via antiSMASH v7 (1).

**__________________________________________________________________**

**A. Microcystins**

**MC-LR**

**
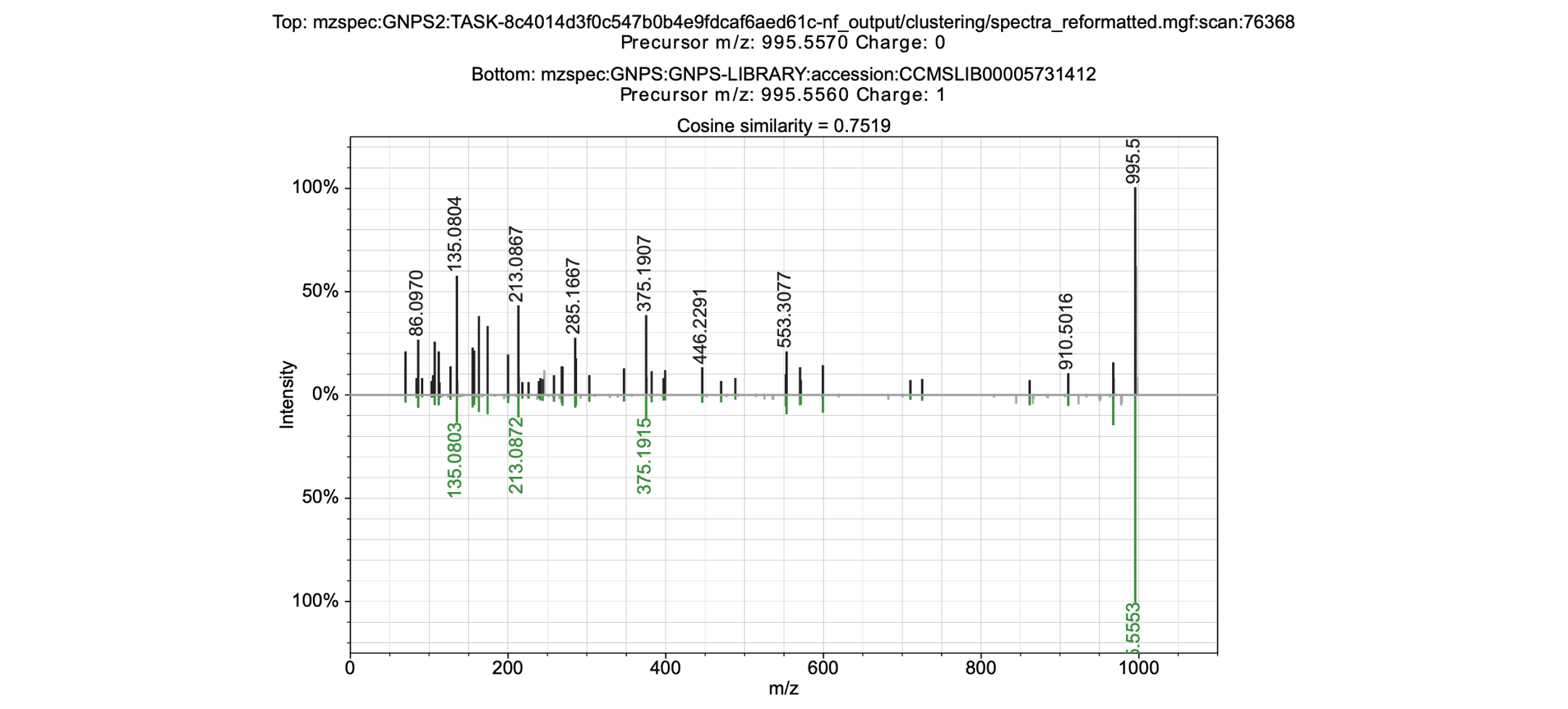
**

**MC-YR**

**
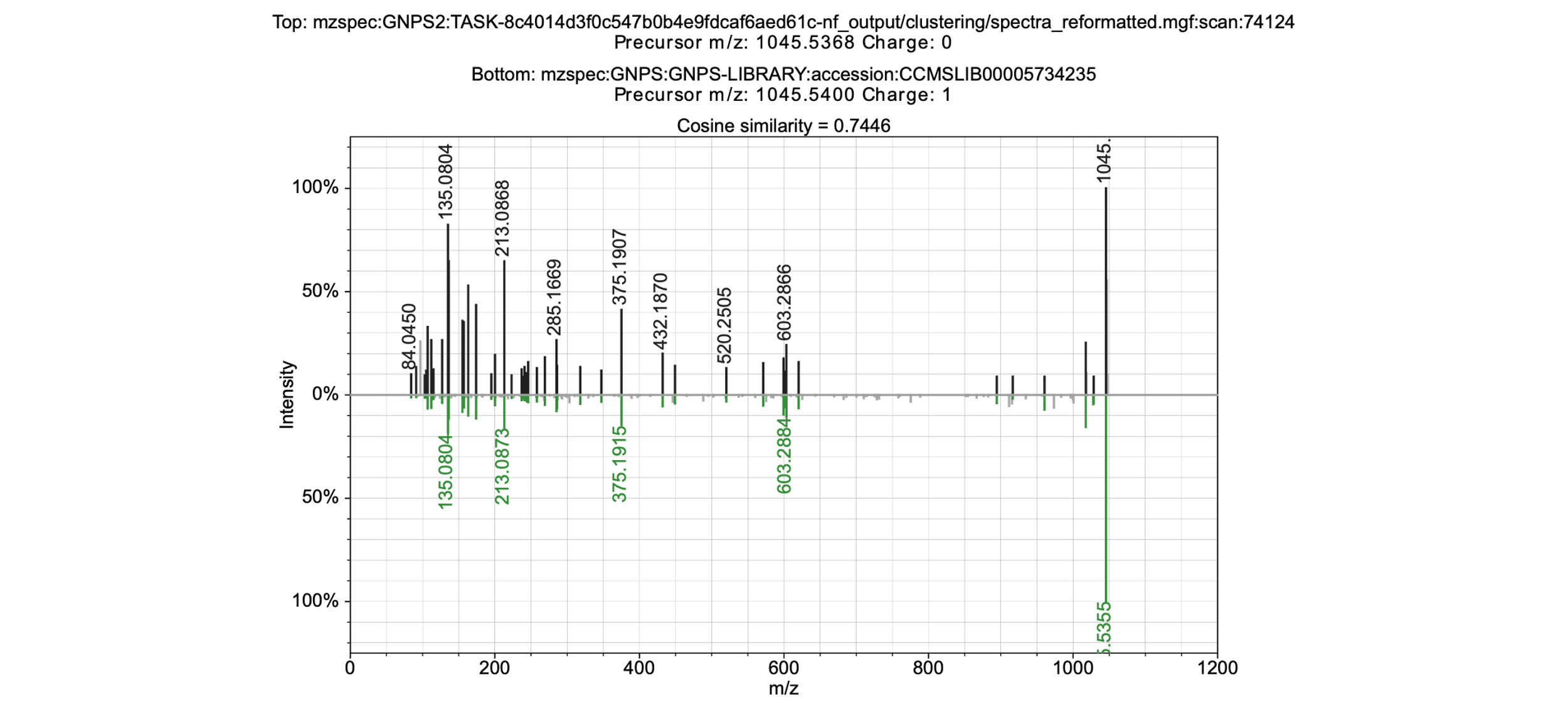
**

**MC-HilR**

**
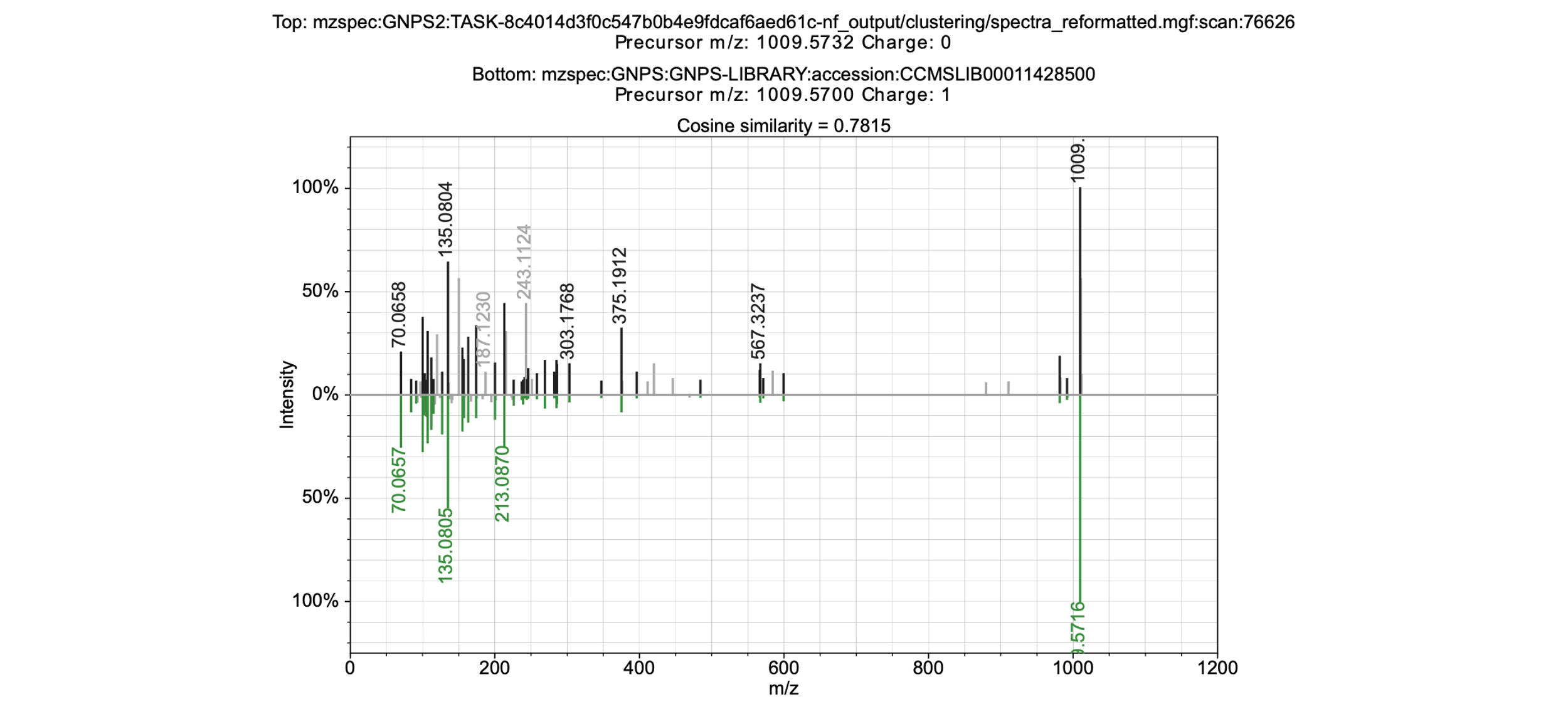
**

**MC-RR**

**
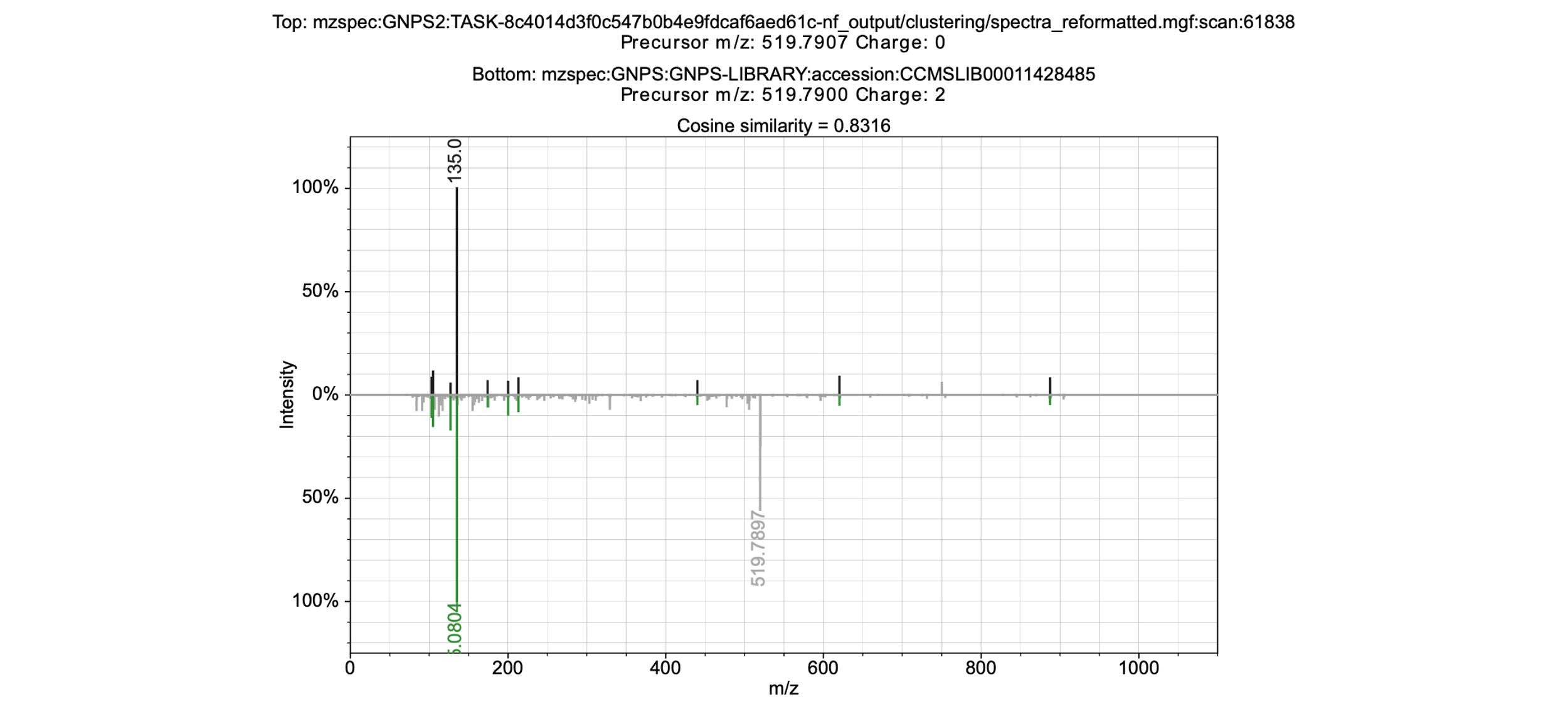
**

**MC-HtyR**

**
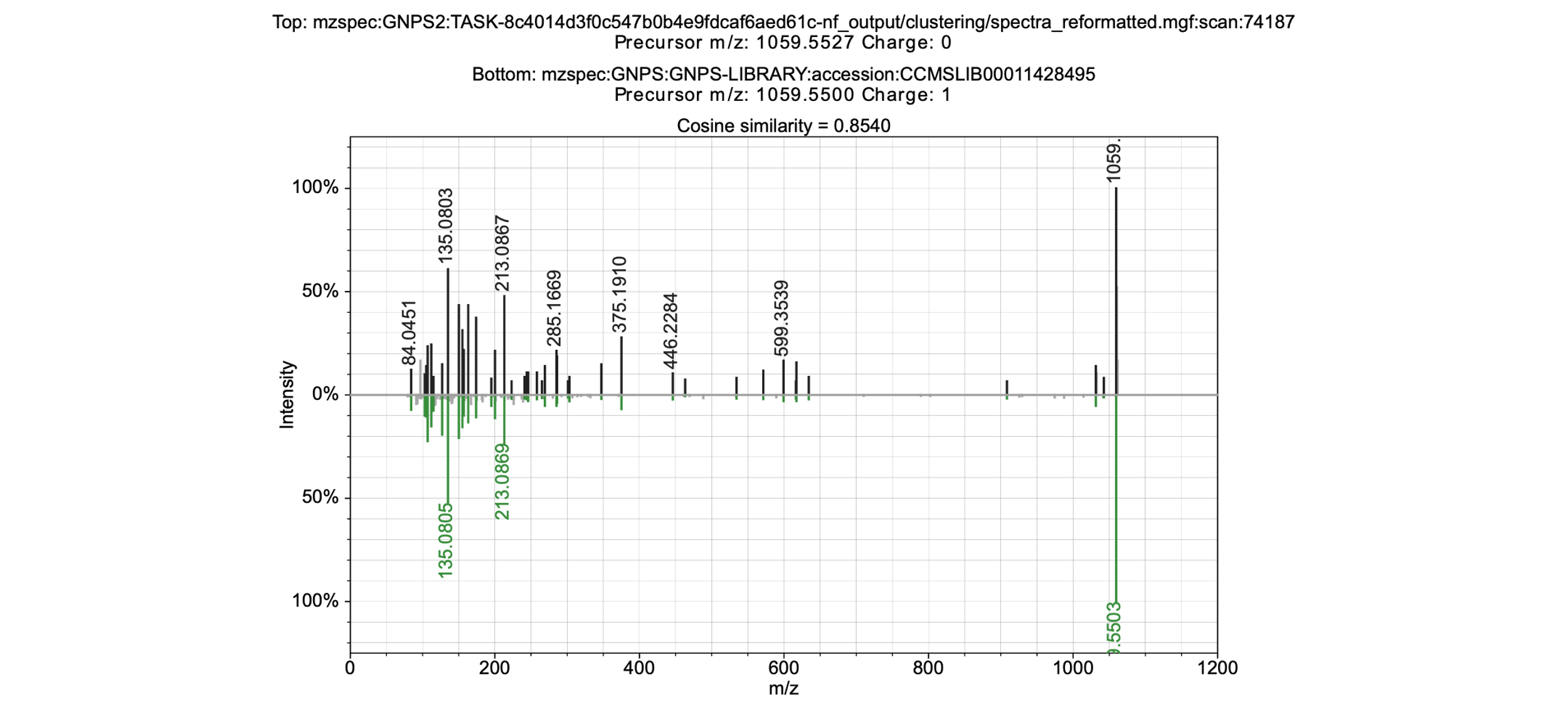
**

**B. Aeruginosin 98A**

**
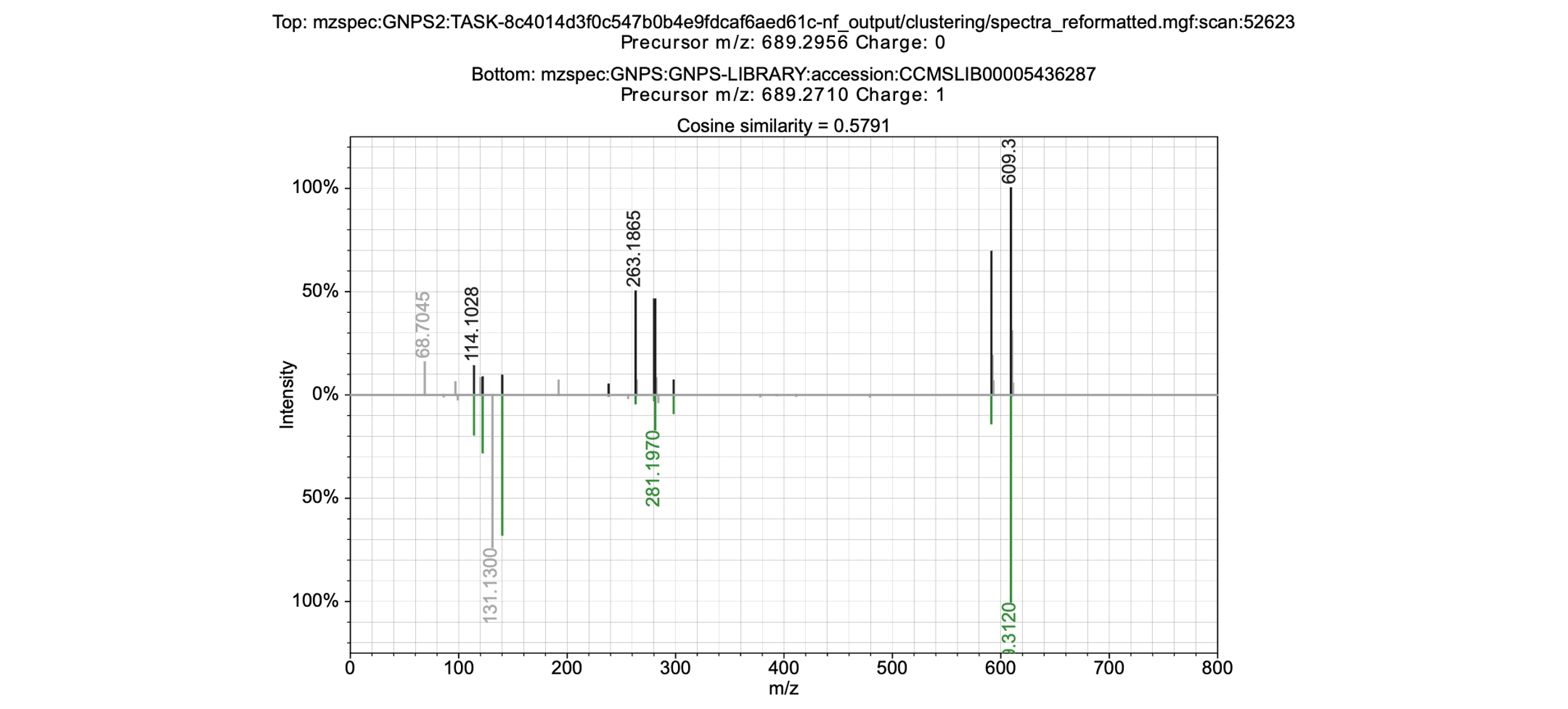
**

**C. Anabaenopeptins**

**Anabaenopeptin A**

**
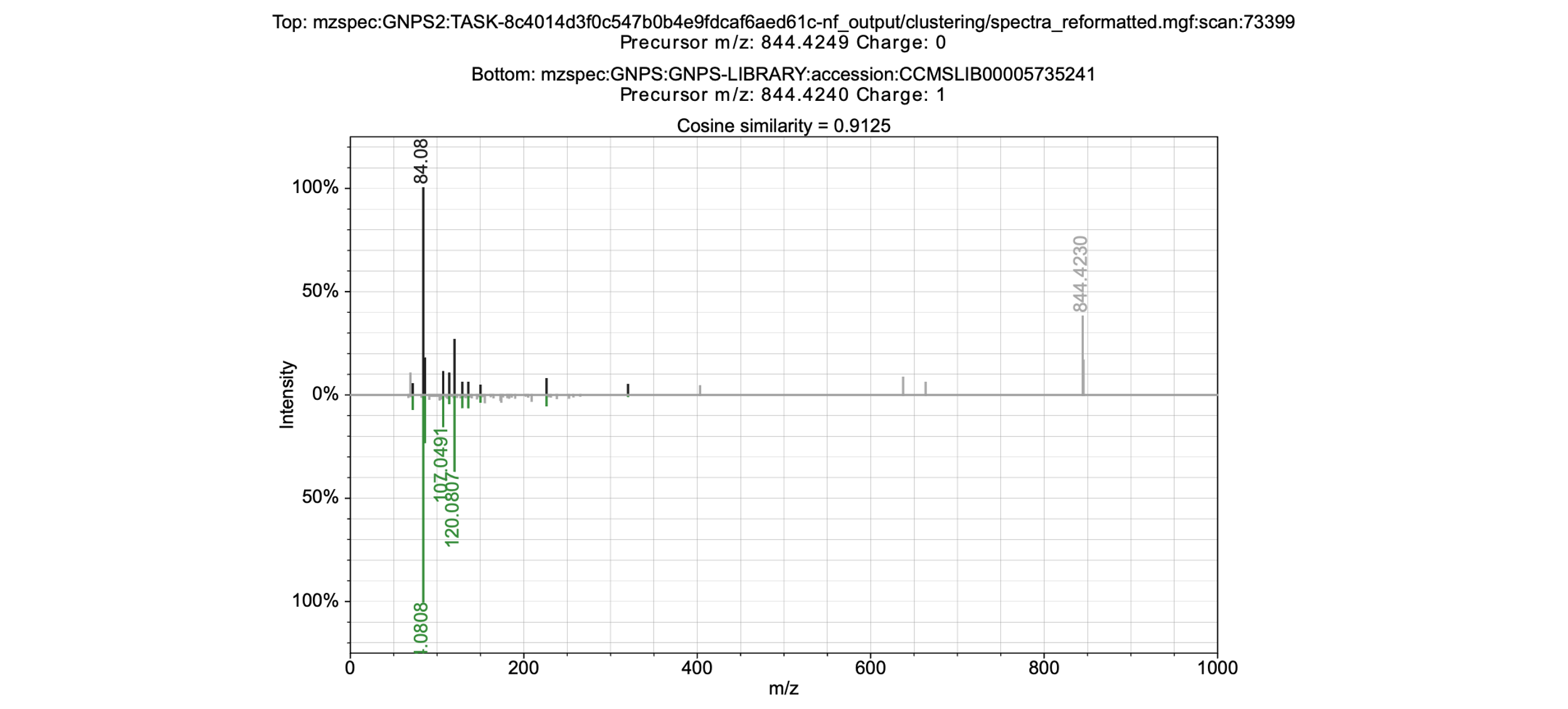
**

**Anabaenopeptin B**

**
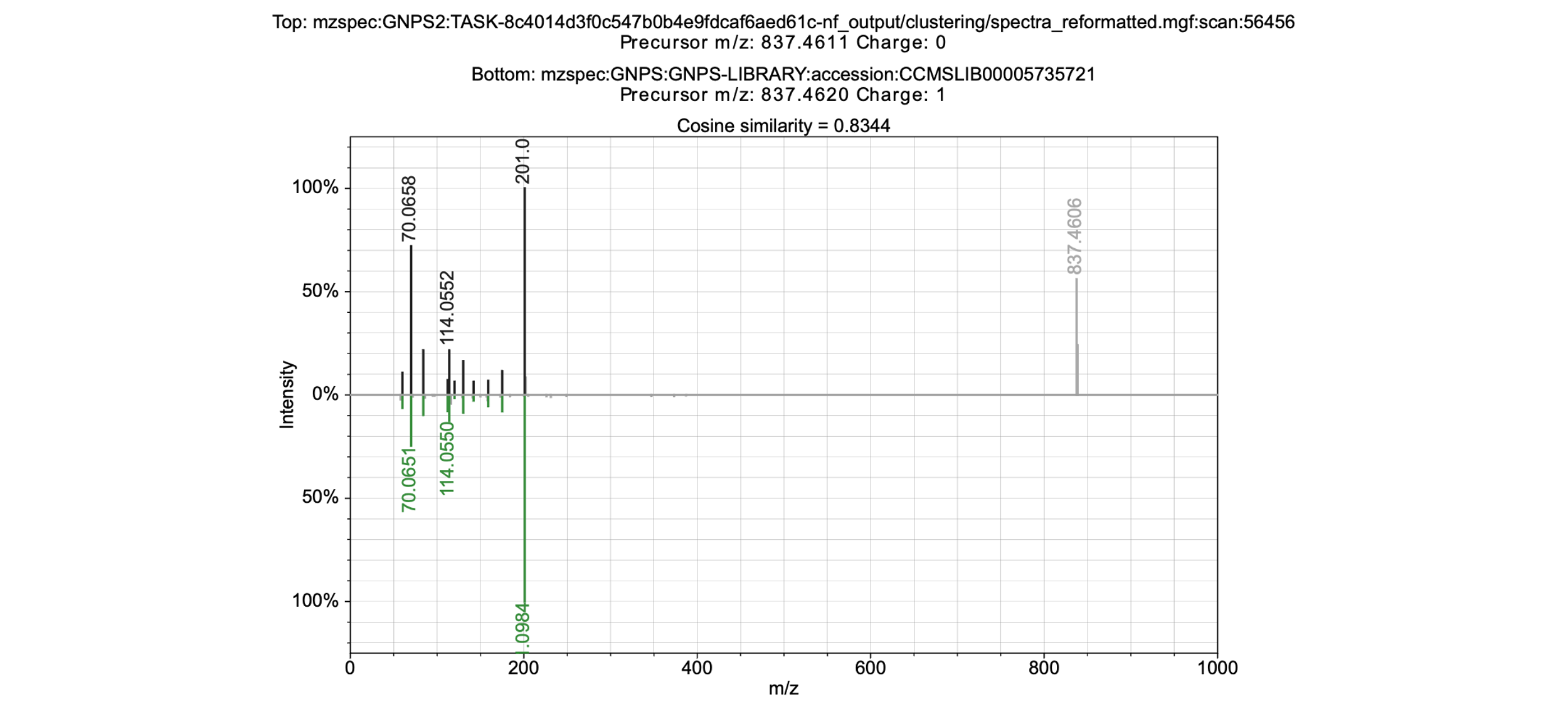
**

**D. Aerucyclamide A**

**
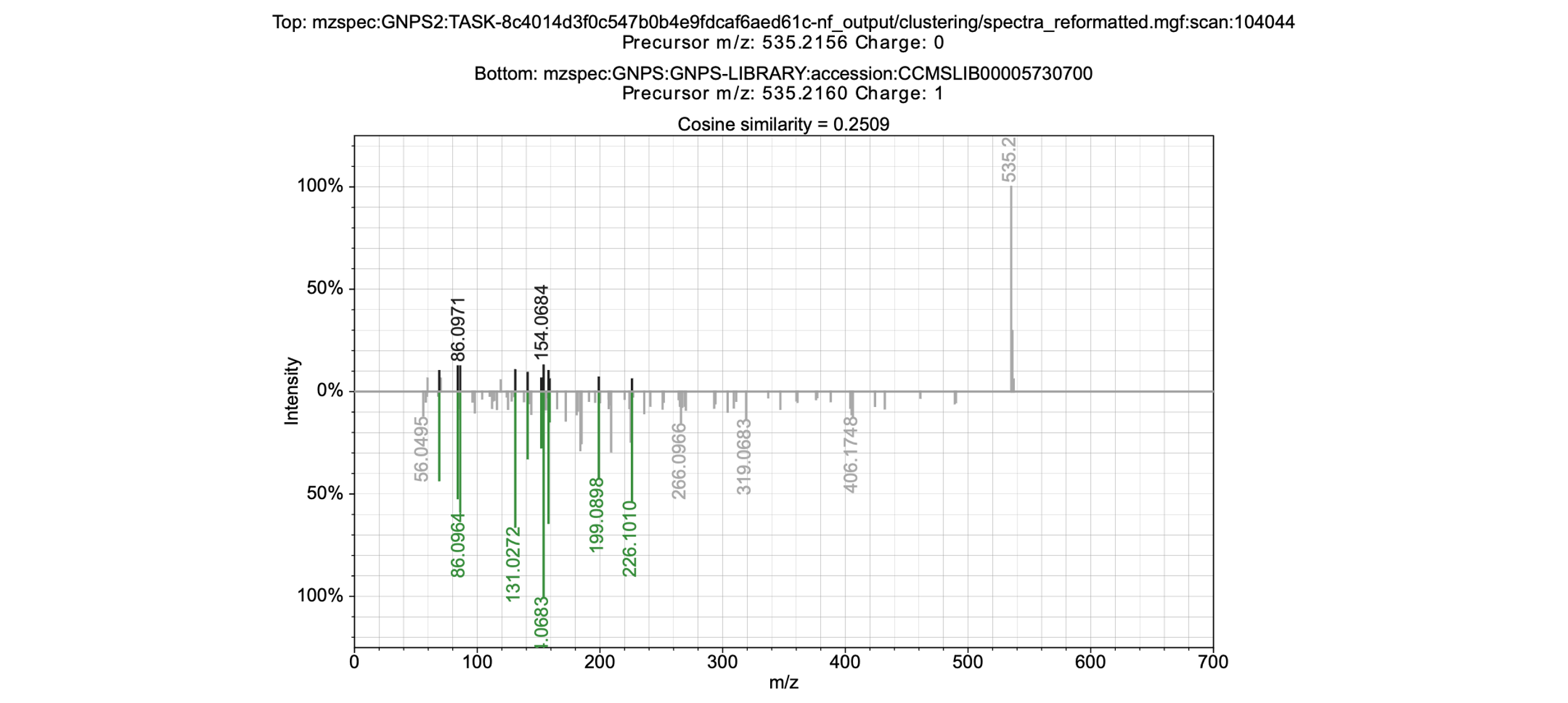
**

**SI Figure 3:** Mirror matches generated by Global Natural Products Social Molecular Networking (GNPS) of library matches detected and used in this study. The black spectrum on top represents the sample spectrum whereas the bottom green spectrum represents the database spectrum for the library match. All metabolite predictions shown here meet at least level 3 criteria as outlined in Schymasnki et al. 2014 (2). **(a)** Spectra putatively annotated as microcystins (MC) annotated as the congeners LR (cosine = 0.836), YR (cosine = 0.822), HilR (cosine = 0.619), RR (cosine = 0.756), HtyR (cosine = 0.736) are shown, **(b)** Aeruginosin 98A (cosine = 0.626), **(c)** Anabaenopeptins A (cosine = 0.855) and B (cosine = 0.912), and **(d)** Aerucyclamide A are shown (cosine = 0.2509).

**__________________________________________________________________**

**
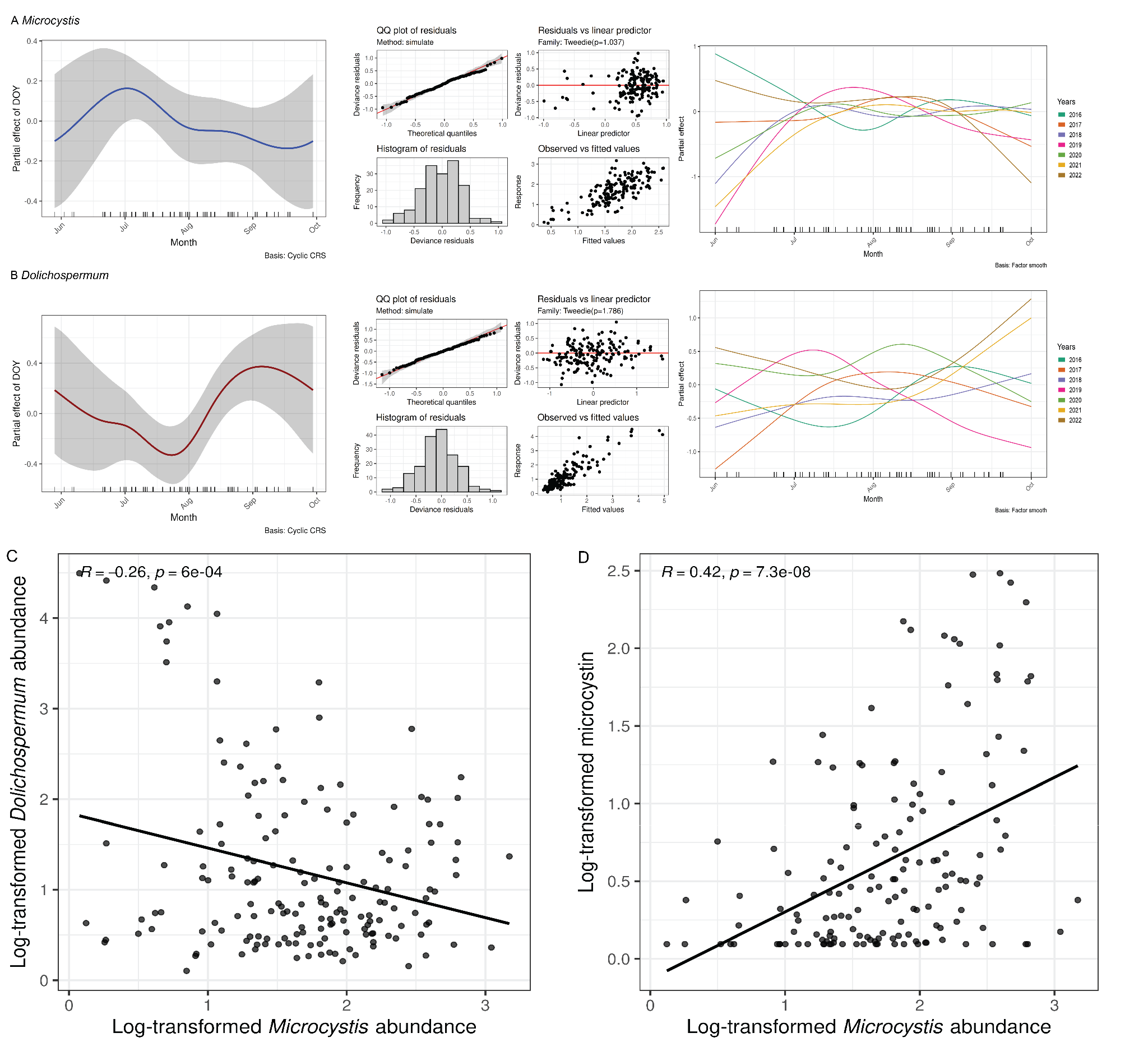
**

**SI Figure 4:** Fixed and partial effects of day of year (DOY), year, and model diagnostics for hierarchical generalized additive models (HGAMs) fit to log-transformed relative abundance of **(a)** *Microcystis* and **(b)** *Dolichospermum***.** The first panels display the fixed effect smooths for DOY based on the HGAM equation described in SI Table 6. Diagnostic plots (second panels) indicate that residuals are homoscedastic and approximately normally distributed (top right panel) and fitted values closely follow observed values (bottom right panel), supporting model adequacy for both taxa. The third panels show partial effect smooths of DOY stratified by year, with year-specific smooths illustrating interannual variation in seasonal patterns of each organism. Tick marks along the x-axis indicate the distribution of observed values. **(c)** A pairwise correlation plot of log-transformed *Microcystis* and *Dolichospermum* relative abundance reveals a modest but significant negative association (Pearson’s r = –0.26, *P* = 6x10^-4^), suggesting potential ecological interaction or shared environmental controls between the two taxa. Because these two response variables are derived from the same biological system, it is likely they are correlated in some manner. However, models were fit independently, and potential interdependence was not explicitly accounted for in the analysis. **(d)** A pairwise correlation plot of log-transformed *Microcystis* and particulate microcystin values (Pearson’s r= 0.42, *P=*7.3e-08*).*

**
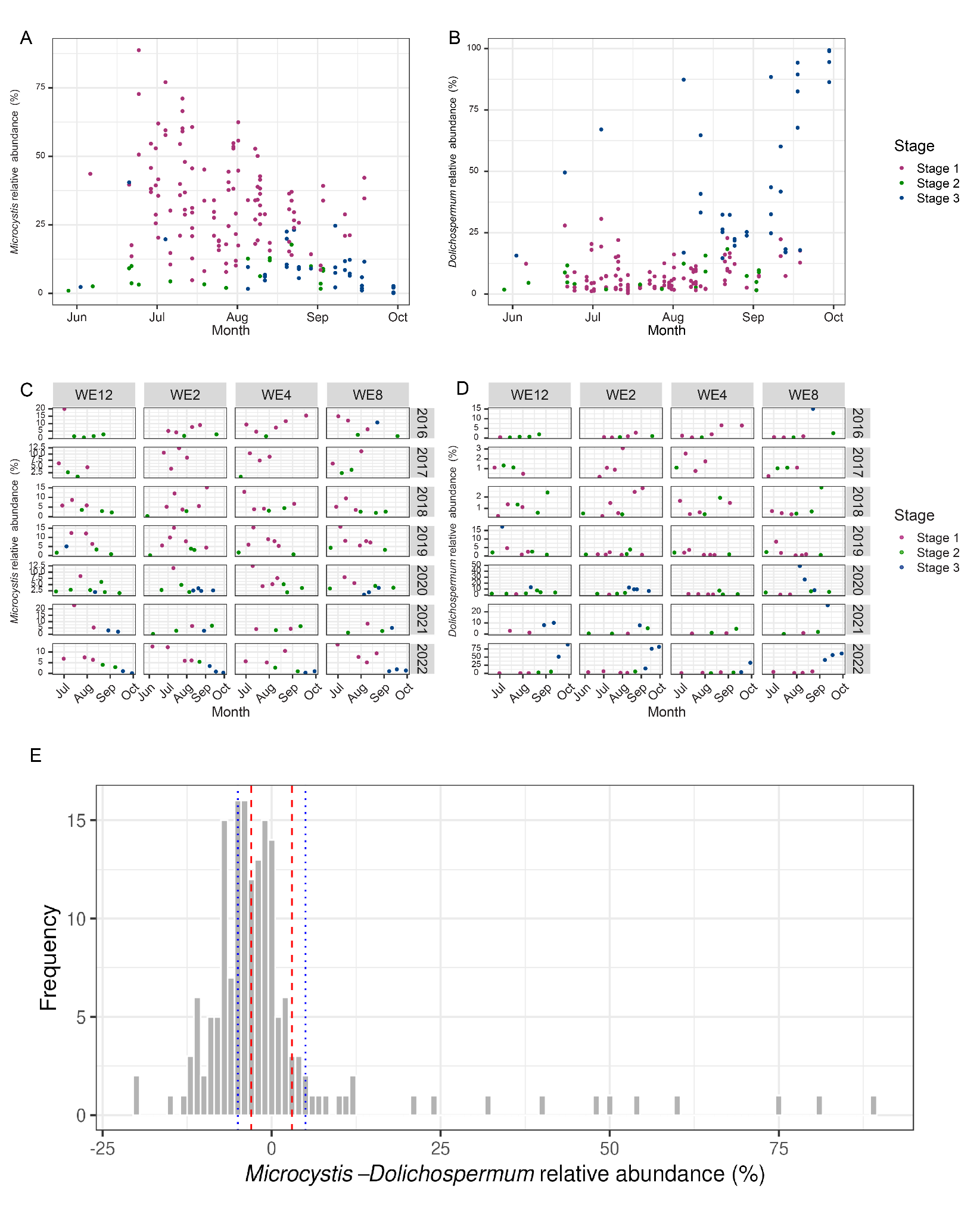
**

**SI Figure 5:** Seasonal pattern of relative abundance (%) of **(a)** *Microcystis* and **(b)** *Dolichospermum* across all stations and years. Points are colored by stage, where Stage 1 indicates *Microcystis* > *Dolichospermum* by 3% or more, Stage 2 indicates approximately equal populations of *Microcystis* and *Dolichospermum* (±3%), and Stage 3 indicates *Dolichospermum* > *Microcystis* by 3% or more. These stages were determined based on the distribution of their relative abundance relative to one another. Seasonal patterns faceted by station (WE12, WE2, WE4, and WE8) and year for **(c)** *Microcystis* and **(d)** *Dolichospermum* relative abundance (%) are also shown, with points colored by stage. **(e)** Distribution of relative abundance difference between *Microcystis* and *Dolichospermum*, with red dashed lines indicating a 3% difference and blue dashed lines indicating a 5% difference.

**
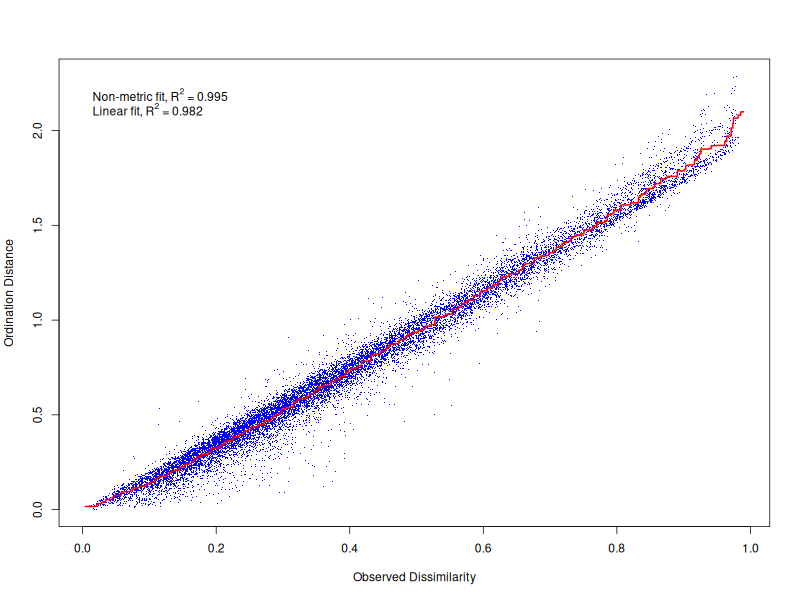
**

**SI Figure 6:** NMDS stress plot assessing the goodness-of-fit of a non-metric multidimensional scaling (NMDS) ordination based on physicochemical parameters. The plot compares observed dissimilarities (x-axis) to distances between points in the ordination space (y-axis). Blue points represent pairwise distances; the red line shows a monotonic fit between observed and ordination distances. The non-metric fit (R² = 0.995) and linear fit (R² = 0.982) indicate an excellent correspondence between the original distance matrix and the reduced-dimensional NMDS space.


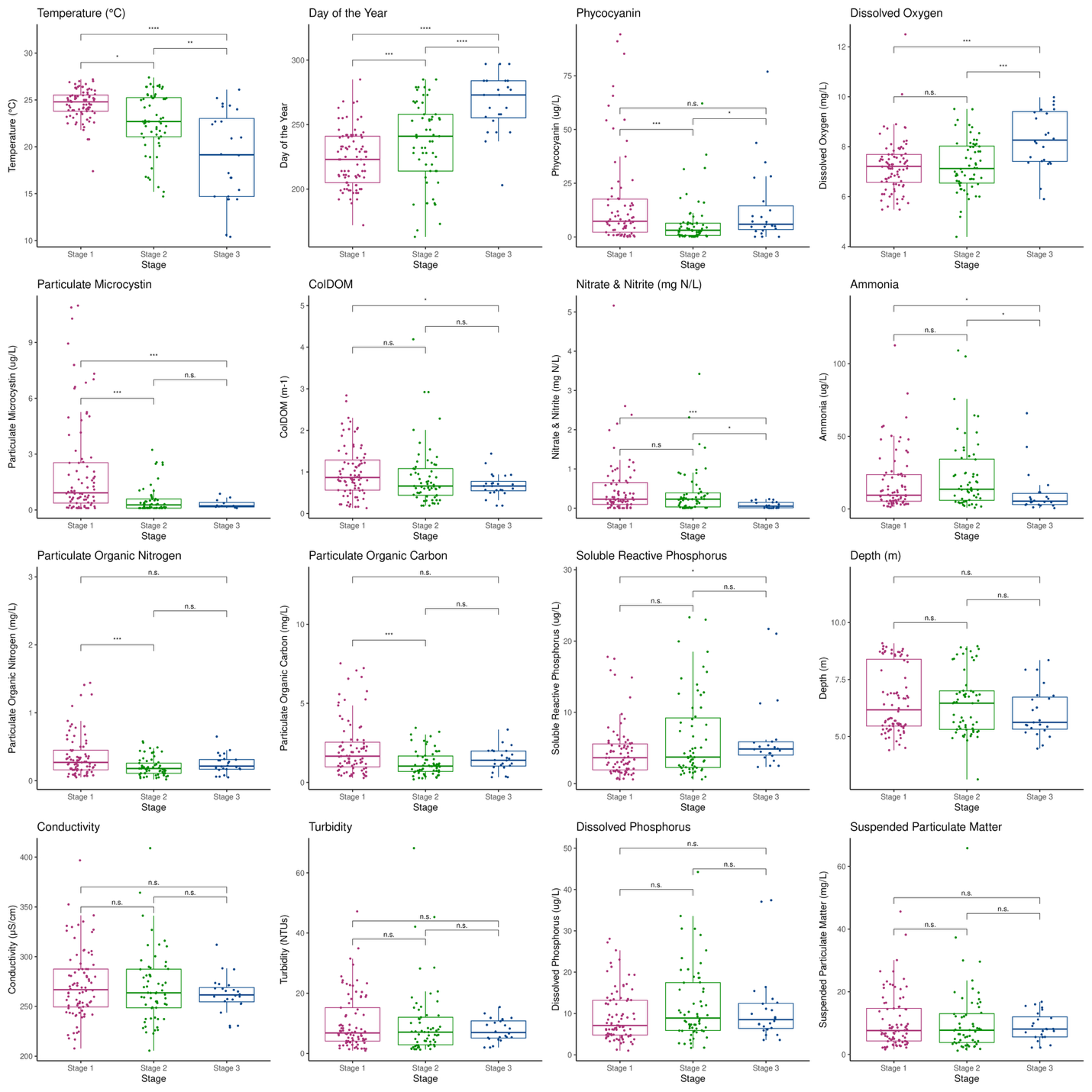


**SI Figure 7:** Boxplots showing the distribution of 18 physicochemical parameters across stages. Each point represents an individual sample, grouped by stage classification. Horizontal bars denote statistically significant pairwise differences between stages for each parameter, based on Kruskal–Wallis tests followed by Dunn’s post hoc comparisons. Significance levels are indicated as: **** *P*<0.0001; *** *P*<0.001; ** *P*<0.01; * *P*<0.5; n.s. = not significant).

**
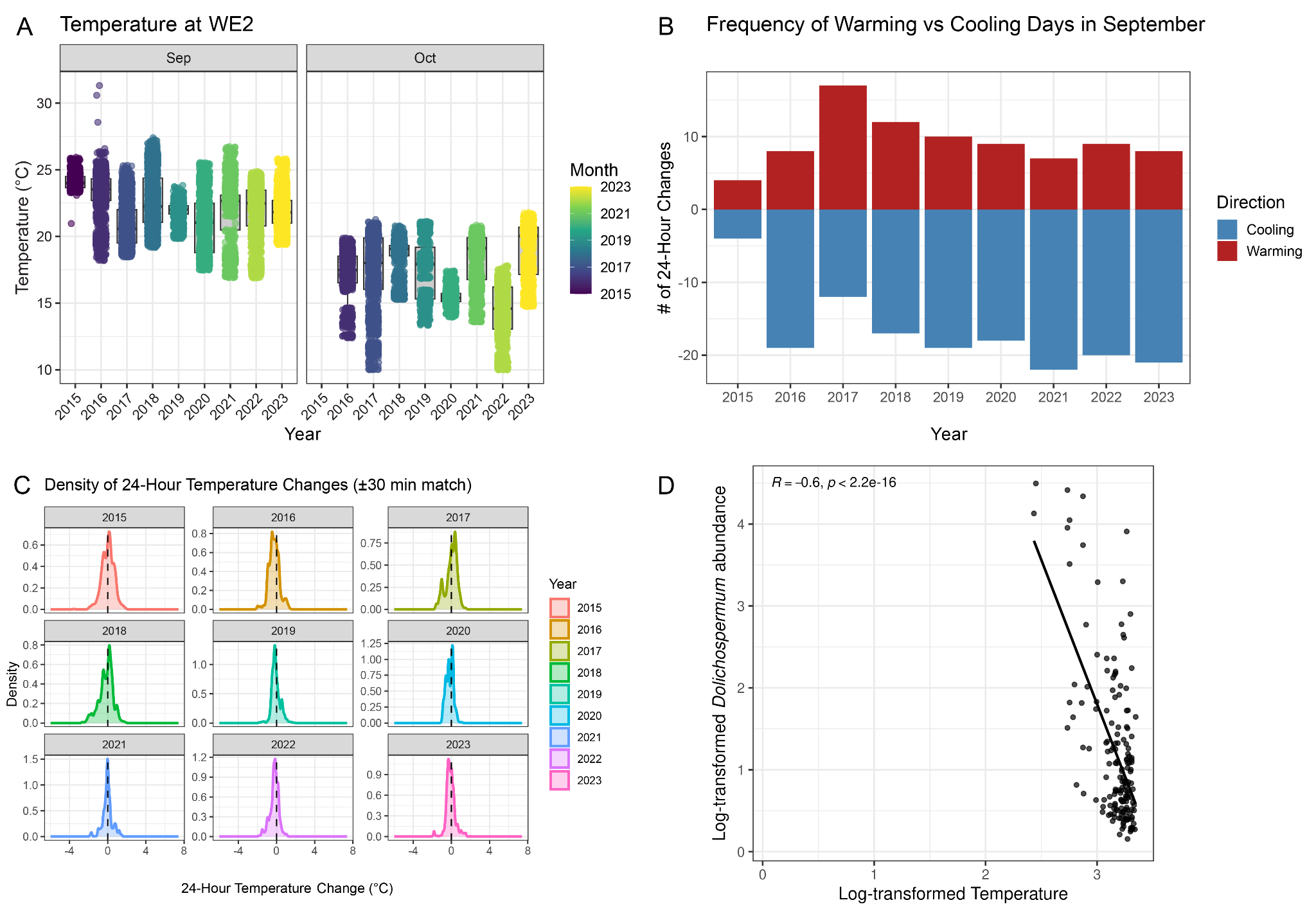
**

**SI Figure 8:** Patterns in surface water temperature from NOAA GLERL WE2 Nutrient Buoy from 2015 through 2023. Data were collected from Great Lakes Observing System (GLOS) archive (3). **(a)** Surface water temperatures (°C) in September and October colored by year. **(b)** Frequency of 24-hour temperature changes in September classified as warming (red) or cooling (blue) days. **(c)** Density distributions of 24-hour temperature changes (magnitude), with a 30-minute observation tolerance, for each year. Vertical dashed lines indicate a change of zero (no net warming or cooling). **(d)** Pairwise comparison between log-transformed *Dolichospermum* abundance and log-transformed temperature (°C). Pearson correlation between the two variables is -0.6 with a *P*-value less than 2.2e-16, indicative of a strong and significant correlation between increasing *Dolichospermum* abundance and decreasing temperature.

**
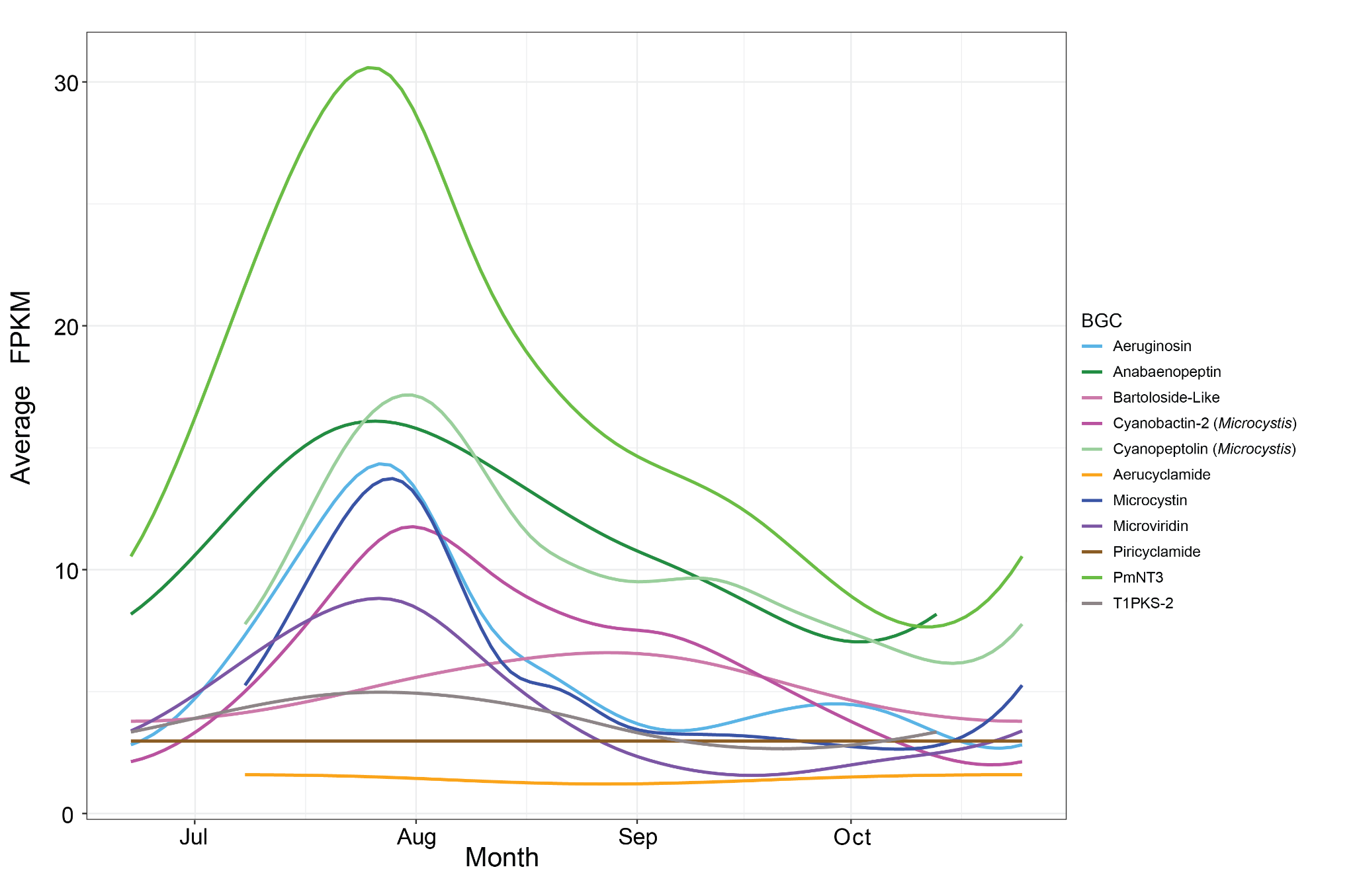
**

**SI Figure 9:** Temporal patterns of average FPKM of BGCs from *Microcystis*. Colored lines represent individual BGCs, smoothed using generalized additive models (GAMs) with a smooth on day of year with a cubic regression function.

**
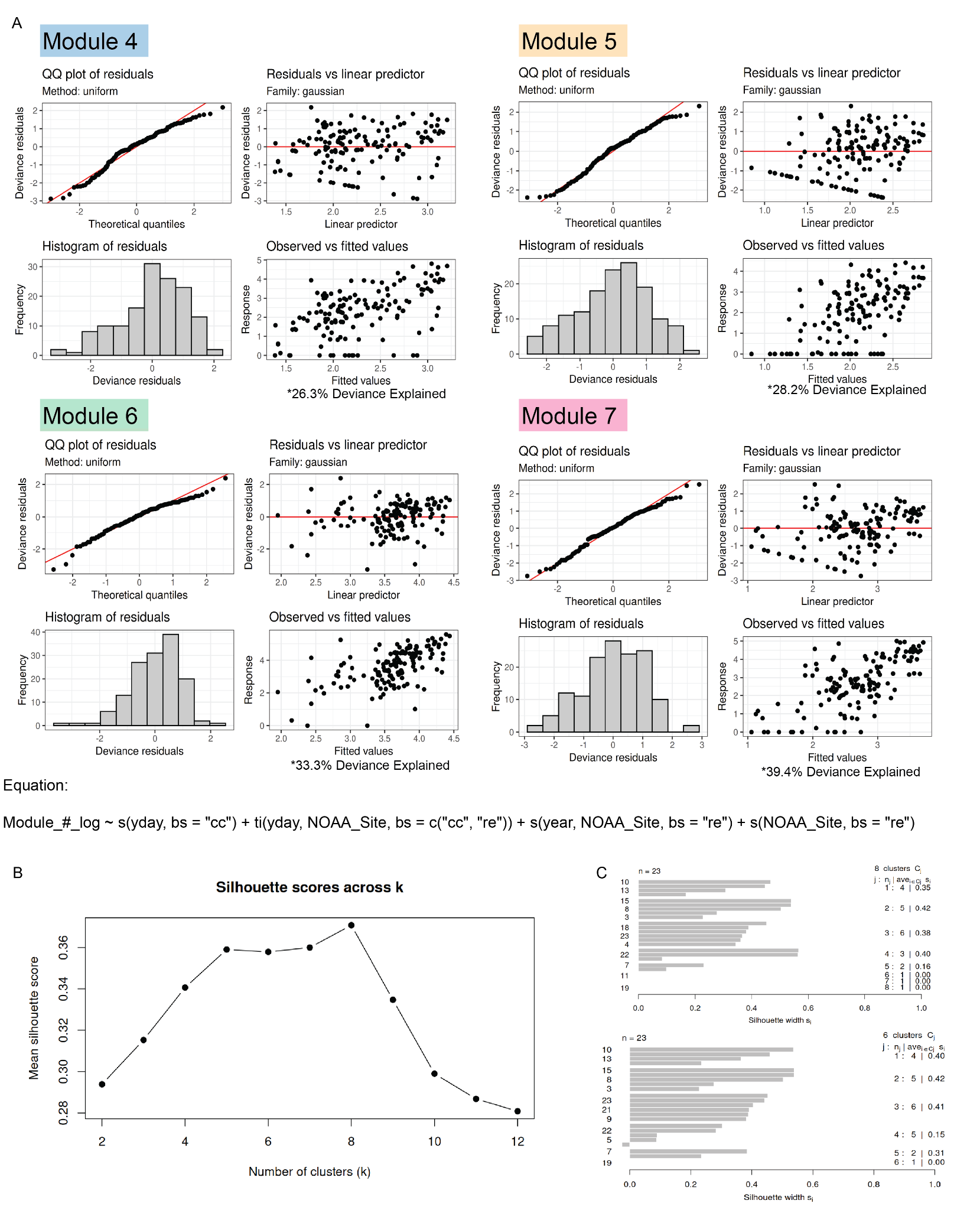
**

**SI Figure 10: (a)** Residual diagnostic plots are based on models using log-transformed biosynthesis module abundance (modules 4, 5, 6, and 7) as the response variable within the equation at the bottom of the figure. Residuals appear randomly scattered around zero (homoscedastic) across predictors (top right panel), and fitted values closely follow observed values (bottom right panel), supporting model adequacy for the modules. **(b)** Mean silhouette scores with changing k values for the creation of biosynthesis modules. **(c)** Silhouette scores for each cluster when k is equal to 8 and 6.

**
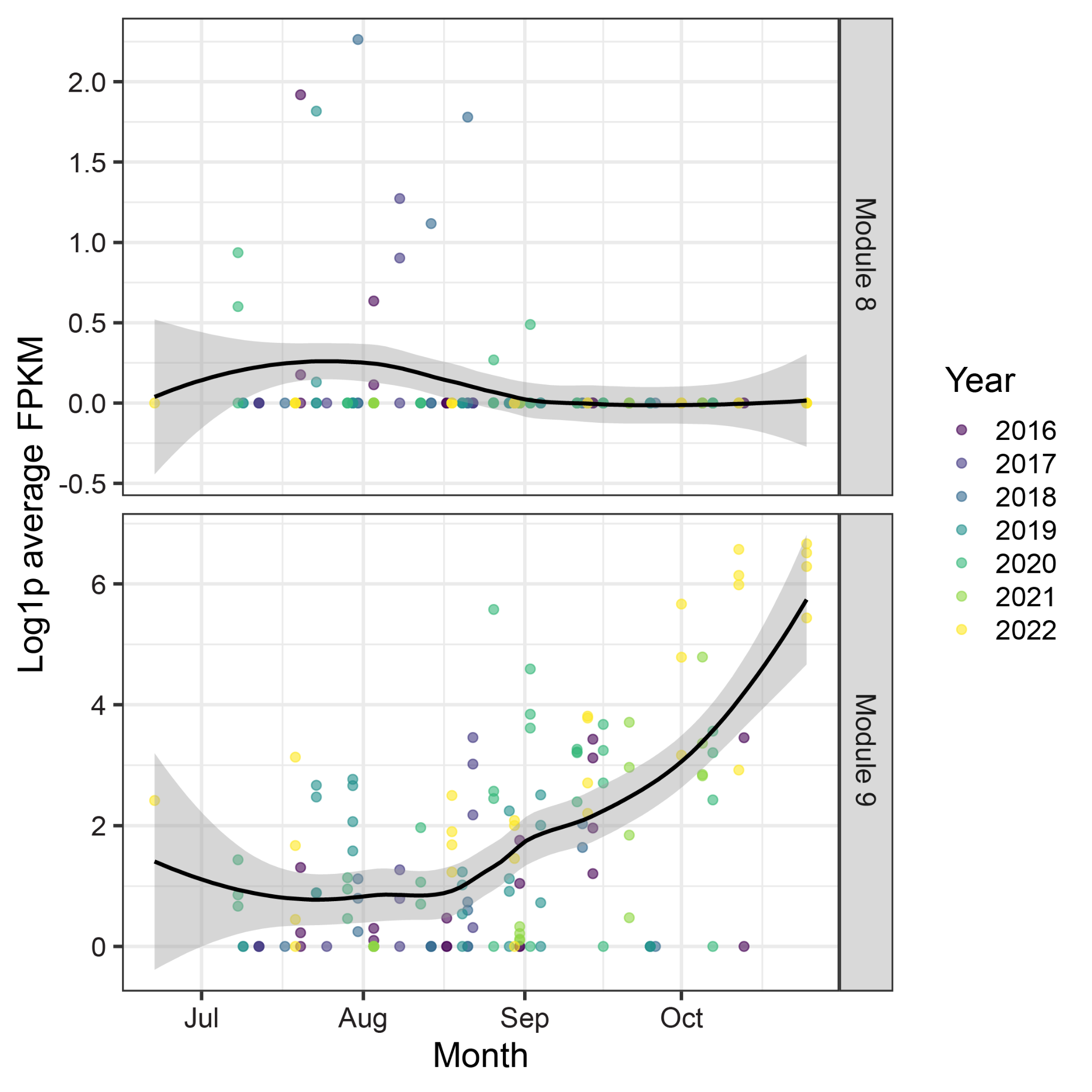
**

**SI Figure 11:** Log FPKM of BGCs from modules 8 and 9 belonging to the *Anabaena, Dolichospermum, Aphanizomenon (ADA)* clade. Individual observations are shown as points colored by year, and smoothed lines are fitted using a loess function. Shaded ribbons indicate standard errors around the fitted temporal patterns.


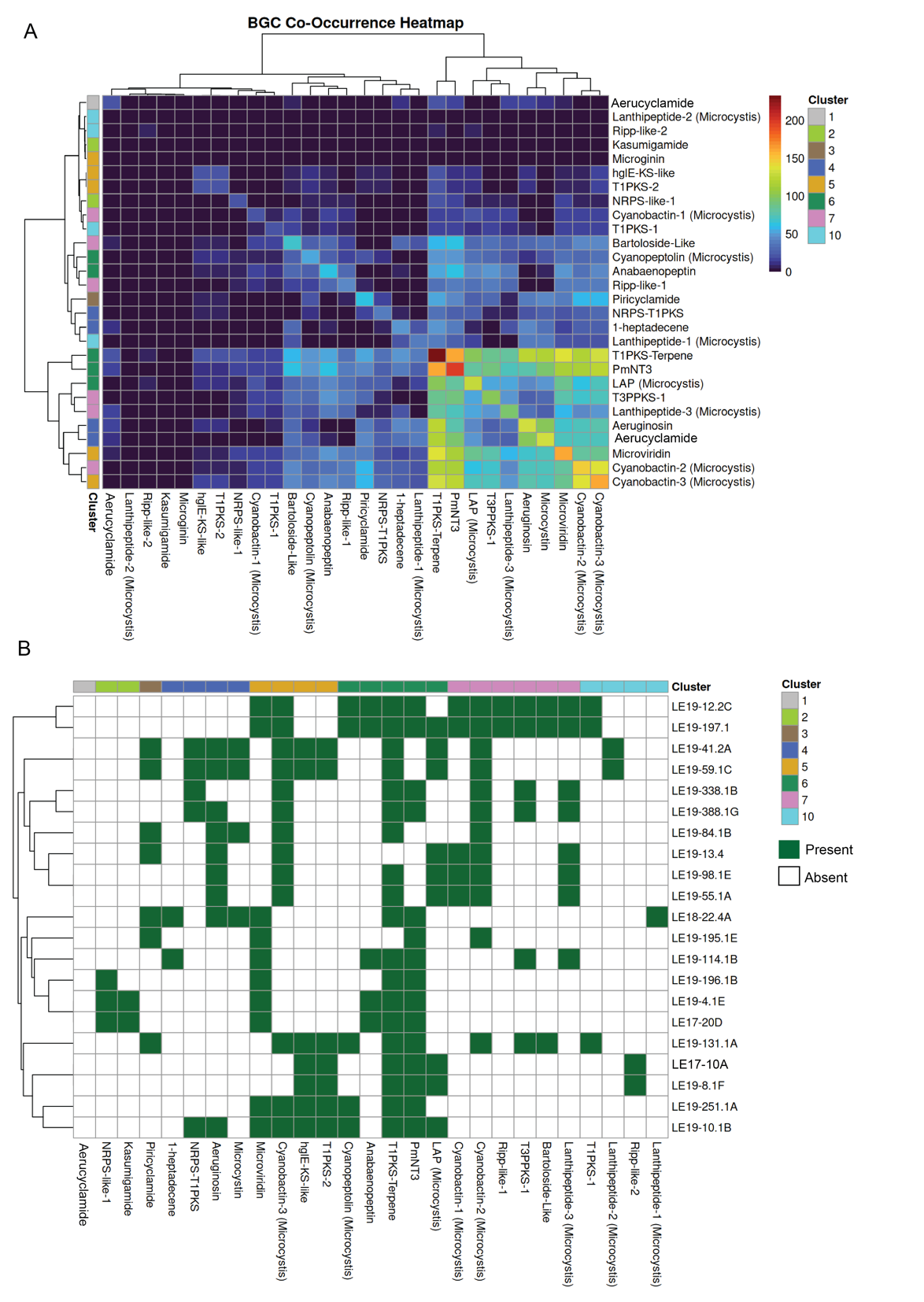


**SI Figure 12: (a)** Co-occurrence matrix of BGCs across publicly available genomes of *Microcystis* deposited in National Center for Biotechnology Information (NCBI). Co-occurrence of BGCs across genomes was calculated and hierarchically clustered using average-linkage. The cluster each BGC belongs to is indicated as the colored bar on the right. Tiles are brighter in the heatmap indicating more co-occurrences between BGCs. **(b)** Presence/absence matrix of BGCs in WLECC isolates, organized by which cluster the BGC is in (colored bar). Green tile indicates the presence of the BGC in the isolate, and a white tile indicates the absence of the BGC in the isolate.

**
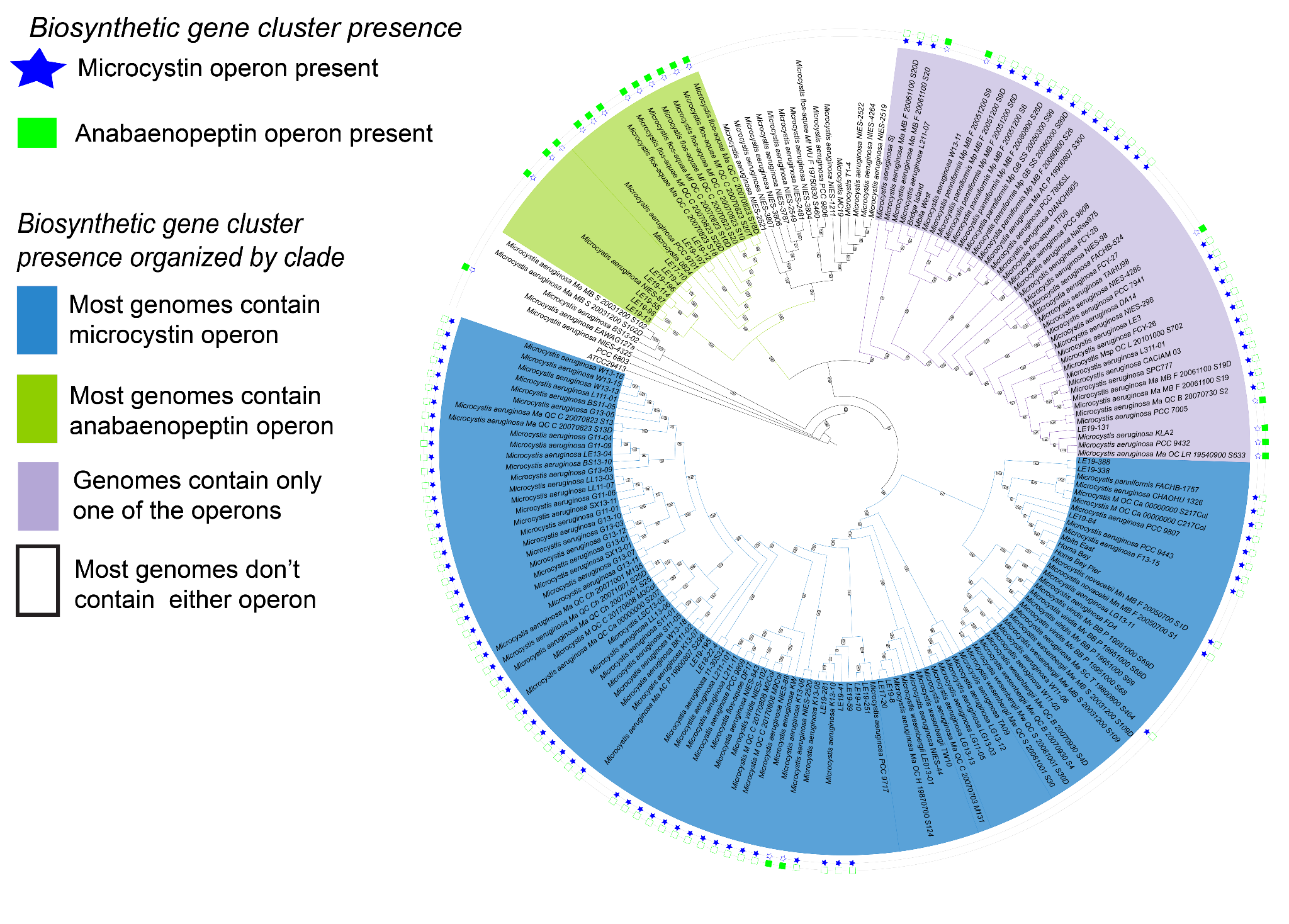
**

**SI Figure 13:** Phylogenomic analysis of publicly available *Microcystis* genomes with *Synechocystis* sp. PCC 6803 used as the outgroup. Genomes were used to build a maximum likelihood phylogenomic tree based on the prepackaged single-copy gene-set for cyanobacteria (251 target genes) in GToTree v1.7.05 (4). Lime green boxes indicate the detection of the anabaenopeptin BGC within a genome, and blue stars indicate the detection of the microcystin BGC within a genome. Clades shaded as blue indicate phylogenomically related genomes mostly containing the microcystin BGC, clades shaded as green indicate phylogenomically related genomes mostly containing the anabaenopeptin BGC, clades in purple indicate phylogenomically related genomes that have either the anabaenopeptin or microcystin BGCs, and clades in white contain phylogenomically related without either of the BGCs. Numbers at nodes indicate bootstrap support values from 1,000 replicates.

**
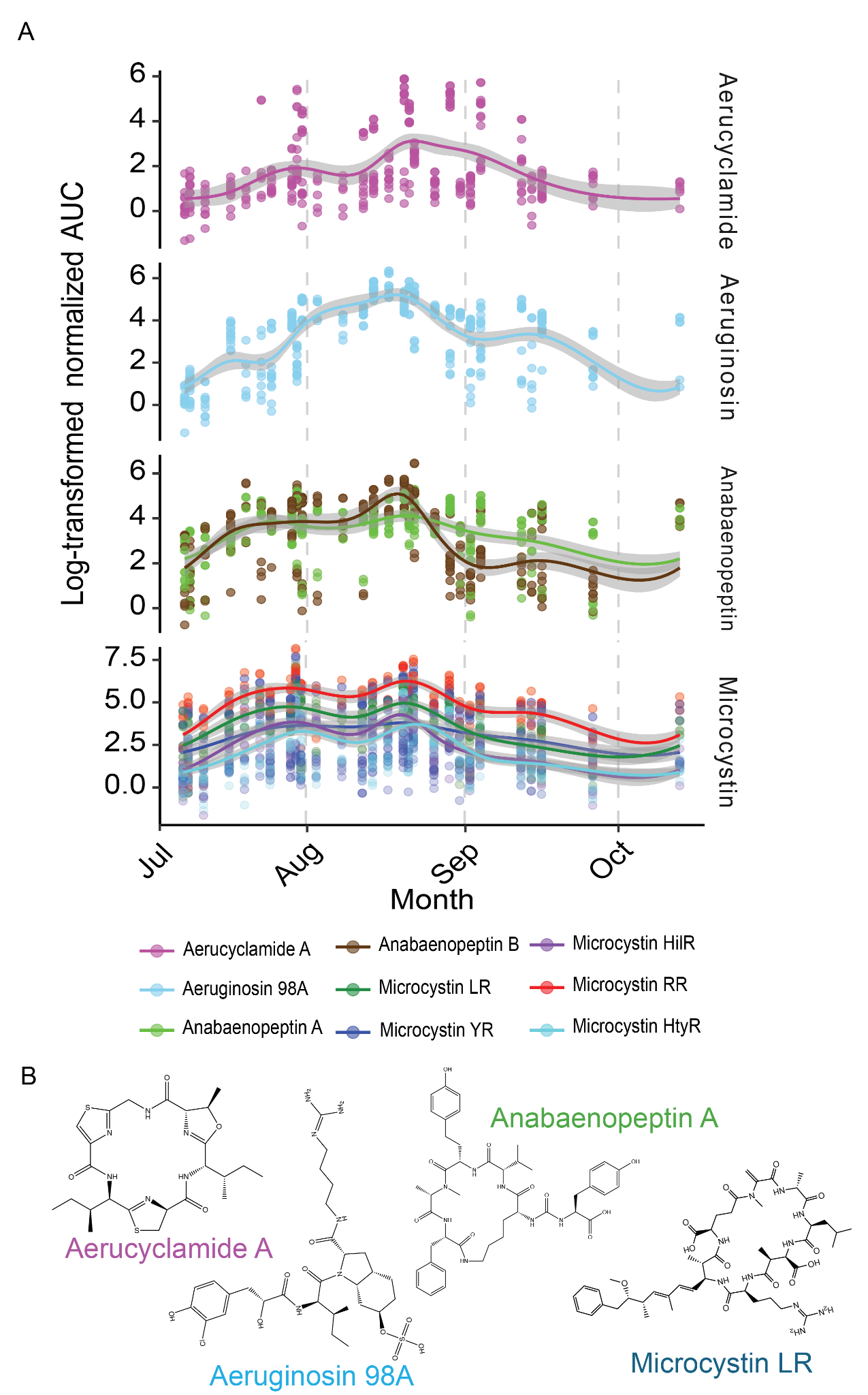
**

**SI Figure 14: (a)** Log-transformed normalized relative abundance (AUC) of metabolites putatively annotated as Aerucyclamide A, Aeruginosin 98A, Anabaenopeptin A and B, and Microcystin LR, YR, HilR, RR, and HtyR. All individual observations are shown, and smoothed lines for each metabolite were fitted using a loess function. **(b)** Representative metabolites from each metabolite class above.

**
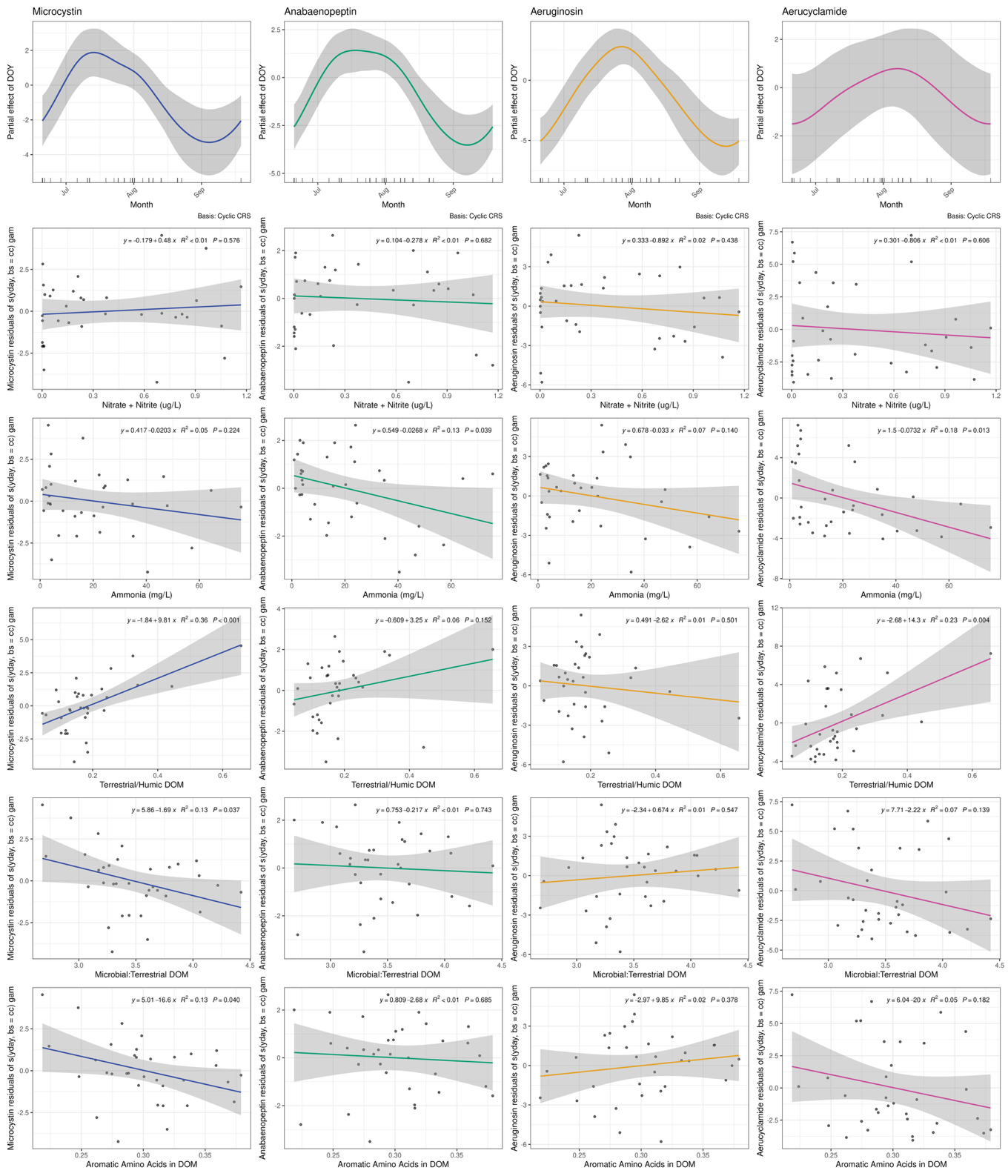
**

**SI Figure 15:** The top row displays partial effects of day of year on log-transformed metabolite abundance from GAMs using a cyclic cubic spline (±95% confidence interval), capturing seasonal patterns for each metabolite (Microcystins, Anabaenopeptins, Aeruginosins, and Aerucyclamides from left to right). The bottom five rows of panels display the relationship between each metabolites’ GAM residuals and an abiotic variable. Residuals represent variability unexplained by seasonal trends, and linear models (±95% confidence intervals) are shown with R² and *P-*values from the fitted linear regression. Each row represents an abiotic variable (Nitrate + Nitrite, Ammonia, Terrestrial/Humic fraction of DOM (Fmax3), Microbial:Terrestrial Ratio of DOM ((Fmax1+4+5)/Fmax3), and Aromatic Amino Acid Portion of DOM ((Fmax4+5)/Fmax3) from top to bottom).

**___________________________________________________________**

**SI Notes**

**SI Note 1:** Metabolomics Data Collection

**HPLC**

Analytes were separated on a Thermo Scientific Vanquish Bioinert high-performance liquid chromatography (HPLC) system (ThermoFisher Scientific, San Jose, CA) with a Waters Atlantis T3 column (3um, 3.0x150mm) (Waters Corp., Milford, MA) at 30°C. A stacked injection method was used to add 2μL 5% EDTA, 2μL 5μg/L simetone (PubChem ID: 12654), and 50μL of sample. All reagents were LC/MS grade except for THF, which was >99.5% and stabilized with butylated hydroxytoluene (BHT). The mobile phases were: (A) water with 0.1% formic acid and 2mM ammonium formate and (B) 50:50 ACN:MeOH with 2mM ammonium formate, 0.1% THF and 0.1% formic acid. Samples were maintained at 4°C in the autosampler and the flow rate was 0.6 ml/min throughout the gradient in **SI Table 2**, with the first two minutes sent to waste.

**Liquid Chromatography-High Resolution Mass Spectrometry (LC/HRMS)**

Mass spectrometric analysis was carried out on a Thermo Fisher Scientific Q Exactive Orbitrap mass spectrometer equipped with a heated electrospray ionization (HESI-II) source in positive ion mode. The spray voltage, capillary temperature and auxiliary gas heater temperature were set to 3.5 kV, 275°C and 450°C, respectively. The sheath gas, auxiliary gas, sweep gas and S-lens RF level were set to 55, 15, 3 (arbitrary units) and 50.0 V, respectively. Nitrogen was used for spray stabilization, for collision-induced dissociation experiments in the higher energy collision dissociation (HCD) cell, and as the damping gas in the C-trap. The instrument was calibrated in positive mode every 7 days using the manufacturer’s calibration solution. The Full MS/ddMS2 mode comprised a full MS scan followed by a data dependent MS2 scan with a fragmentation energy applied. The mass spectrometer acquired a full MS scan at a resolution of 70,000 (FWHM at 200 m/z). The automatic gain control (AGC) target was set at 3.0 × 10e^6^ with a maximum injection time (IT) of 100 ms. The full MS scan ranges were set as 150–2000 m/z. For the dd-MS2 scans, the mass resolution was set at 35,000 FWHM, AGC target at 5 × 10e^4^, maximum IT 50 ms, isolation window 5.0 m/z, intensity threshold 2 × 10e^3^, exclude isotopes “on”, and dynamic exclusion 2.0s. The fragmentation energy (NCE) was stepped with values of 15, 40, and 60. Instrument control and data processing were conducted with Xcalibur 4.2, Compound Discoverer 3.3, and Exactive Tune 2.11 software (Thermo Scientific).

The data generated from the quantitation runs was used to do a pre-emptive search of the CyanoMetDB using Compound Discoverer 3.3 (5). The compounds that were discovered from the database were added to the inclusion list. The inclusion list is a list of compounds that will be preferentially fragmented. A blank was also run. The m/z of analytes in the blank were placed in an exclusion list. These m/z will not be selected for fragmentation. If there was an analyte that was found in the exclusion list that may interfere with a desired analyte, either the compound was removed from the exclusion list or the time segment that it was part of the exclusion list was edited so that it would not interfere with the analytes of interest.

___________________________________________________________

**SI Note 2:** Metagenomic processing information

Raw metagenomic reads were processed using fastp v0.23.2 (6). Clumpify was used to sort reads and improve compression efficiency (<http://sourceforge.net/projects/bbmap/>, accessed June 2024). BBmap was used to remove human contaminant reads (GENCODE release 38). Metagenomic assemblies were produced using MEGAHIT v1.2.9 with the meta-sensitive preset (7). Multiple binning software were used to maximize metagenome assembled genome (MAG) recovery including CONCOCT v1.1.0, MetaDecoder v.1.0.13, VAMB v.3.08, MetaBAT2 v2.17, MaxBin 2.0 v2.2.7, and SemiBin v.1.03 (8–12). Only MAGs with CheckM completeness > 30% and <50% contamination were used prior/subsequent to dRep. All MAGs were grouped and dereplicated using dRep v3.2.0 at 95 percent (13). Only bacterial and archaeal MAGs were considered, in addition to the relative abundance of unmapped reads in the community assuming the average MAG size per the CoverM relative abundance method.

The relative abundance of WLECC *Microcystis* isolates (NCBI BioProject: PRJNA903891) was calculated as described in Yancey et al. 2023A (14). Briefly, sourmash gather v4.5.0 was used to compare k-mer sketches (k=31) of WLECC *Microcystis* MAGs to sampled metagenomes using the GTDB release 202 database and 159 *Microcystis* reference genomes (15). Relative abundance of isolates was calculated as described in Irber et al. 2022 (16).

**__________________________________________________________________**

**SI Note 3**: BGC Database Creation

To analyze the presence and abundance of BGCs across metagenomics samples in this study, a comprehensive, non-redundant, gene-based BGC database was created (Key to database in **SI Table 3**). All files and code to produce the final BGC database used in this study can be found on GitHub (<https://github.com/Geo-omics/2016-2022-Western-Lake-Erie-Time-Series-Metagenomics-and-Metabolomics>, accessed January 2026). First, all genes from BGCs belonging to the cyanobacterial genera *Planktothrix, Sphaerospermopsis, Dolichospermum, Anabena, Aphanizomenon, Microcystis, Cylindrospermopsis,* and *Cyanobium* were pulled into a fasta file from the MIBiG 3.0 database (17). All genes from BGCs identified from 21 isolates in the Western Lake Erie Culture Collection, described in Yancey and Hart et al. 2024 (18), were pulled into the same fasta file, resulting in Seed 1 of the database. Seed 2 of the database was produced by removing any BGCs that putatively synthesize the same product and were derived from a genome from the same genera. This database was clustered using MMseqs2 at 95 percent minimum sequence id and 90 percent coverage to remove similar genes from the database (19). To ensure non-redundancy, different BGCs that shared genes 95 percent or more similar were marked to be removed from the database, with one of the BGCs being kept in the database. This non-redundant gene database resulted in seed 3. To ensure there was fair representation of geographically relevant BGCs from the *Anabaena, Aphanizomenon, Dolichospermum* (ADA) clade, BGCs were mined from nine highly curated ADA MAGs from WLE described in Den Uyl et al. 2024 (20). BGCs were detected using antiSMASH v7, run within the multismash tool (https://github.com/zreitz/multismash) (--minimal, --genefinding-tool prodigal, --cb-known, --cc-mibig, --tigrfam) (1,21). Identified BGCs smaller than 5,000 base pairs were excluded, and remaining BGCs were put into a fasta file and clustered at 95 percent identity and 90 percent coverage using MMseqs2 to reduce redundancy (19). Non-redundant ADA genes from the BGCs were merged with seed 3 into seed 4 of the database. Lastly, seed 4 was clustered using MMseqs2 at 95 percent identity and 90 percent coverage to ensure an entirely non-redundant database of genes within BGCs. Any BGCs putatively encoding the synthesis of terpenes were excluded in this study. This final database is seed 5 and was used for identifying BGCs across metagenomic samples in this study. Each BGC in seed 5 is visualized in **SI Figure 1**. A key to the genes within the seed 5 database are also provided in GitHub, where the type of gene (core, additional, transporter, regulatory, other) is annotated for each gene (as provided by antiSMASH v7) (1).

**_________________________________________________________________**

**References**

1. Blin K, Shaw S, Augustijn HE, Reitz ZL, Biermann F, Alanjary M, et al. antiSMASH 7.0: new and improved predictions for detection, regulation, chemical structures and visualisation. Nucleic Acids Research. 2023 Jul 5;51(W1):W46–50.

2. Schymanski EL, Jeon J, Gulde R, Fenner K, Ruff M, Singer HP, et al. Identifying Small Molecules via High Resolution Mass Spectrometry: Communicating Confidence. Environ Sci Technol. 2014 Feb 18;48(4):2097–8.

3. NOAA. NOAA WE2 Nutrient Buoy Data from 2015-2023 [Internet]. Great Lakes Environmental Research Laboratory; [cited 2025 Jul 1]. Available from: https://seagull-erddap.glos.org/erddap/tabledap/GLERLWE2_archive.html

4. Lee MD. GToTree: a user-friendly workflow for phylogenomics. Bioinformatics. 2019 Oct 15;35(20):4162–4.

5. Jones MR, Pinto E, Torres MA, Dörr F, Mazur-Marzec H, Szubert K, et al. CyanoMetDB, a comprehensive public database of secondary metabolites from cyanobacteria. Water Res. 20210308th ed. 2021 May 15;196:117017.

6. Chen S, Zhou Y, Chen Y, Gu J. fastp: an ultra-fast all-in-one FASTQ preprocessor. Bioinformatics. 2018 Sep 1;34(17):i884–90.

7. Li D, Liu CM, Luo R, Sadakane K, Lam TW. MEGAHIT: an ultra-fast single-node solution for large and complex metagenomics assembly via succinct de Bruijn graph. Bioinformatics. 20150120th ed. 2015 May 15;31(10):1674–6.

8. Alneberg J, Bjarnason BS, de Bruijn I, Schirmer M, Quick J, Ijaz UZ, et al. Binning metagenomic contigs by coverage and composition. Nat Methods. 20140914th ed. 2014 Nov;11(11):1144–6.

9. Liu CC, Dong SS, Chen JB, Wang C, Ning P, Guo Y, et al. MetaDecoder: a novel method for clustering metagenomic contigs. Microbiome. 2022 Mar 10;10(1):46.

10. Kang DD, Li F, Kirton E, Thomas A, Egan R, An H, et al. MetaBAT 2: an adaptive binning algorithm for robust and efficient genome reconstruction from metagenome assemblies. PeerJ. 2019 Jul 26;7:e7359.

11. Wu YW, Tang YH, Tringe SG, Simmons BA, Singer SW. MaxBin: an automated binning method to recover individual genomes from metagenomes using an expectation-maximization algorithm. Microbiome. 2014 Dec;2(1):26.

12. Pan S, Zhu C, Zhao XM, Coelho LP. A deep siamese neural network improves metagenome-assembled genomes in microbiome datasets across different environments. Nat Commun. 2022 Apr 28;13(1):2326.

13. Olm MR, Brown CT, Brooks B, Banfield JF. dRep: a tool for fast and accurate genomic comparisons that enables improved genome recovery from metagenomes through de-replication. The ISME Journal. 2017 Dec 1;11(12):2864–8.

14. Yancey CE, Kiledal EA, Chaganti SR, Denef VJ, Errera RM, Evans JT, et al. The Western Lake Erie culture collection: A promising resource for evaluating the physiological and genetic diversity of *Microcystis* and its associated microbiome. Harmful Algae. 2023 Jul;126:102440.

15. Brown CT, Irber L. sourmash: a library for MinHash sketching of DNA. Journal of Open Source Software. 2016 Sep 14;1(5):27.

16. Irber L, Brooks PT, Reiter T, Pierce-Ward NT, Hera MR, Koslicki D, et al. Lightweight compositional analysis of metagenomes with FracMinHash and minimum metagenome covers. bioRxiv; 2022. p. 2022.01.11.475838.

17. Terlouw BR, Blin K, Navarro-Muñoz JC, Avalon NE, Chevrette MG, Egbert S, et al. MIBiG 3.0: a community-driven effort to annotate experimentally validated biosynthetic gene clusters. Nucleic Acids Research. 2023 Jan 6;51(D1):D603–10.

18. Yancey CE, Hart L, Hefferan S, Mohamed OG, Newmister SA, Tripathi A, et al. Metabologenomics reveals strain-level genetic and chemical diversity of *Microcystis* secondary metabolism. mSystems. 2024 Jun 25;0(0):e00334-24.

19. Steinegger M, Söding J. MMseqs2 enables sensitive protein sequence searching for the analysis of massive data sets. Nat Biotechnol. 2017 Nov;35(11):1026–8.

20. Den Uyl P. Genetic characterization of saxitoxin-producing cyanobacteria associated with western Lake Erie harmful algal blooms [Internet]. University of Michigan; 2024. Available from: https://deepblue.lib.umich.edu/bitstream/handle/2027.42/193158/MS_Thesis_DenUyl_DeepBlue_FinalSignatures.pdf?sequence=1&isAllowed=y

21. Hyatt D, Chen GL, Locascio PF, Land ML, Larimer FW, Hauser LJ. Prodigal: prokaryotic gene recognition and translation initiation site identification. BMC Bioinformatics. 2010 Mar 8;11:119.
